# Ultramacrocyclization in water *via* external templation†

**DOI:** 10.1039/d1sc06236k

**Published:** 2022-01-05

**Authors:** Qiong Chen, Ye Lei, Guangcheng Wu, Qing Li, Yuanjiang Pan, Hao Li

**Affiliations:** Department of Chemistry Institution, Zhejiang University Hangzhou 310027 China lihao2015@zju.edu.cn; Key Laboratory of Macrocyclic and Supramolecular Chemistry, Guizhou University Guiyang 550025 China nyliqing2012@163.com; ZJU-Hangzhou Global Scientific and Technological Innovation Center Hangzhou 310027 China

## Abstract

Condensing a dihydrazide and each of a series of cationic bisaldehyde compounds bearing polymethylene chains in weakly acidic water produces either a macrocycle in a [1 + 1] manner or its dimer namely a [2]catenane, or their mixture. The product distribution is determined by the length of the bisaldehydes. Addition of cucurbit[8]uril (CB[8]) drives the catenane/macrocycle equilibria to the side of macrocycles, by forming ring-in-ring complexes with the latter. When the polymethylene unit of the bisaldehyde is replaced with a more rigid *p*-xylene linker, its self-assembly with the dihydrazide leads to quantitative formation of a [2]catenane. Upon addition of CB[8], the [2]catenane is transformed into an ultra-large macrocycle condensed in a [2 + 2] manner, which is encircled by two CB[8] rings. The framework of this macrocycle contains one hundred and two atoms, whose synthesis would be a formidable task without the external template CB[8]. Removal of CB[8] with a competitive guest leads to recovery of the [2]catenane.

## Abstract 2

Condensing a bisaldehyde and a bisacylhydrazide in water in the presence of cucurbit[8]uril, produced an ultra-large ring whose framework contains more than one hundred atoms.

## Graphical abstract

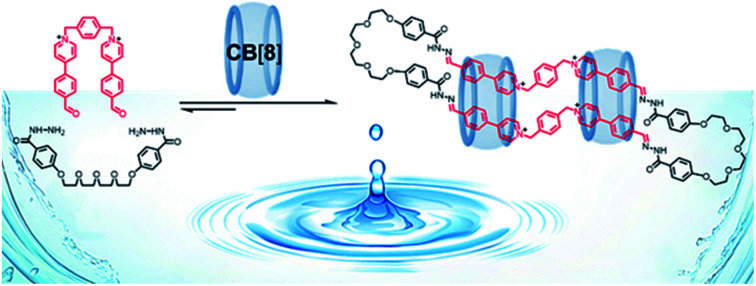

## Introduction

Synthesis of macrocyclic compounds represents one of the major focuses in the field of supramolecular chemistry.^1^ When the sizes of the rings become larger, their production becomes more challenging, given that macrocyclization of rings containing more atoms implies more entropy loss. High dilution conditions^2^ and/or addition of feasible guest templates^3^ have proven to be efficient approaches to suppress or avoid oligomeric or polymeric byproducts. However, these approaches become less useful in the case that the sizes of macrocyclic products are ultra-large, especially for those rings containing more than one hundred atoms. In addition, finding a feasible internal guest template to fit within the cavity of an ultra-large macrocycle becomes more difficult, despite a few exceptions.^4^

Our group^5^ and others^6^ discovered that hydrazone condensation represents an ideal dynamic covalent reaction that is amenable to use in water. A variety of [2]catenanes,^5*a*,*d*–*f*^ rings,^5*c*,*d*,6*d*,*e*^ and cages^5*b*,*g*^ were self-assembled in aqueous media, thanks to the reversible nature of hydrazone that allows the systems to perform error checking. In addition, cucurbit[8]uril (CB[8])^7*c*,*e*,*g*,*i*,*k*^ was observed as a host that is able to accommodate a two π-electron guest, driven by the hydrophobic effect and dipole–cation interactions in the case of cationic guests such as pyridinium derivatives. We thus envision that by taking advantage of the marriage of dynamic covalent chemistry^5,6,8^ and external templation based on CB[8] rings,^7^ we might be able to self-assemble an ultra-large macrocycle. Here, by condensing a dihydrazide and a dicationic bisaldehyde containing a poly-methylene chain in water, rings with medium sizes containing fifty to sixty atoms in their framework were self-assembled in a [1 + 1] manner as the major products. When the polymethylene chain becomes longer, the macrocycles start to undergo equilibration with their dimerized form namely [2]catenanes, driven by the hydrophobic effect. In the presence of CB[8], the macrocycle-[2]catenane equilibria shifted to the side of macrocycles, each of which was accommodated within the cavity of a CB[8] ring, forming a set of [2]pseudorotaxanes. The formation of these ring-in-ring complexes^9^ is favored by the CB[8]–pyridinium interactions between the host and the guest. When the poly-methylene chain in the bisaldehyde was changed to a more rigid *p*-xylene unit, its self-assembly with the dihydrazide linker resulted in the exclusive formation of a [2]catenane in close to a quantitative yield, thanks to the dynamic nature of hydrazone bonds that allows error checking. Addition of CB[8] led to decomposition of this [2]catenane. However, the putative [2]pseudorotaxane was not obtained as occurred in the case of the aforementioned polymethylene counterparts. This is probably on account of the *p*-xylene linker, which renders the [1 + 1] macrocycle too large to fit within the CB[8] cavity. Instead, the bisaldehyde and bishydrazide underwent condensation in a [2 + 2] manner, forming an ultra-large macrocycle that was encircled by two CB[8] rings. This macrocycle contains more than one hundred atoms in its framework, whose formation would be rather challenging without the external template CB[8] rings. Upon removal of the CB[8] by adding a competitive guest, the ultra-large macrocycle underwent decomposition, regaining the [2]catenane.

## Results and discussion

Each of the biscationic dialdehyde compounds, including 1x^2+^·2Br^−^ (x = a, b, c, d and e), contains two cationic pyridinium-benzaldehydes that are bridged by a polymethylene chain containing three to seven CH_2_ units. A dihydrazine linker 2, contains two phenylacylhydrazide units that are connected by a glycol chain. The detailed synthetic procedures are described in the ESI.†

**Fig. 1:**
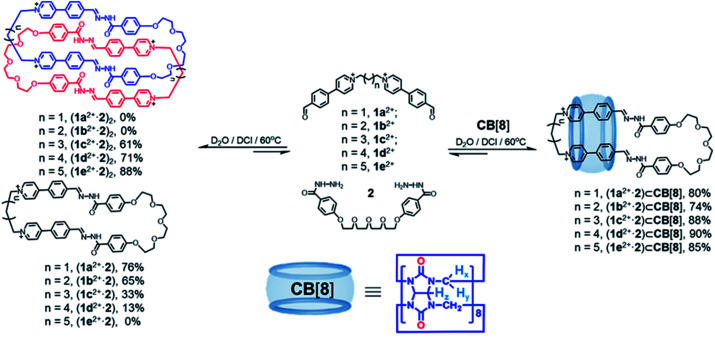
Structural formulae of a series of dicationic bisaldehydes 1x^2+^ (x = a, b, c, d and e) and a bishydrazide 2. A series of dicationic macrocycles (1x^2+^·2) (x = a, b, c and d), as well as a set of tetracationic [2]catenanes (1x^2+^·2)_2_ (x = c, d and e) were self-assembled by combining each of the bisaldehydes and the bishydrazide in water. Addition of CB[8] into each of these self-assembly products led to formation of a series of [2]pseudorotaxanes (1x^2+^·2)⊂CB[8] (x = a, b, c, d and e). Charges are balanced by Cl^−^ or Br^−^ counteranions, which are omitted here for the sake of clarity. The yields shown are determined by ^1^H NMR spectroscopy. It seems that the counterions have little impact on the self-assembly.

Self-assembly was performed by combining the dihydrazine linker 2 (2.5 mM) with each of biscationic dialdehydes 1x^2+^·2Br^−^ (x = a, b, c, d and e; 2.5 mM) in D_2_O. A catalytic amount of DCl was used to catalyze hydrazone condensation (Fig. 1 and Schemes S5–S7†). After heating the corresponding solutions at 60 °C for no less than 8 h, the corresponding ^1^H NMR spectra remained unchanged, showing that all the reactants were almost completely consumed. In the case of 1a^2+^ and 1b^2+^, two macrocycles (1a^2+^·2) and (1b^2+^·2), whose counteranions could be either Cl^−^ or Br^−^, were self-assembled in a [1 + 1] manner as the major products, as indicated by both ^1^H NMR spectroscopy (Fig. S2 and S11†) and mass spectrometry (Fig. S1 and S10†). The yields of (1a^2+^·2) and (1b^2+^·2) were determined to be 76% and 65%, respectively, by using DMSO as an internal standard in the ^1^H NMR samples (Fig. S62B and S63B†). In the case of 1c^2+^ and 1d^2+^, the self-assembly products included both the macrocycles namely (1c^2+^·2) and (1d^2+^·2), as well as their corresponding dimerized forms namely the [2]catenanes (1c^2+^·2)_2_ and (1d^2+^·2)_2_, whose counteranions could be either Cl^−^ or Br^−^. The NMR yields of the [2]catenanes (1c^2+^·2)_2_ and (1d^2+^·2)_2_, as well as the rings (1c^2+^·2) and (1d^2+^·2) were calculated to be 61%, 71%, 33% and 13%, respectively (Fig. S64B and S65B†). In the case of the biscationic dialdehyde 1e^2+^ containing the longest polymethylene namely a (CH_2_)_7_ chain, a [2]catenane (1e^2+^·2)_2_ (counteranions could be either Cl^−^ or Br^−^) was produced as the only observable product in the ^1^H NMR spectrum, whose yield is 88% (Fig. S66B†). Obviously, a dialdehyde with a longer polymethylene chain favors the formation of a [2]catenane to a more significant extent compared to its shorter counterparts.

**Fig. 2:**
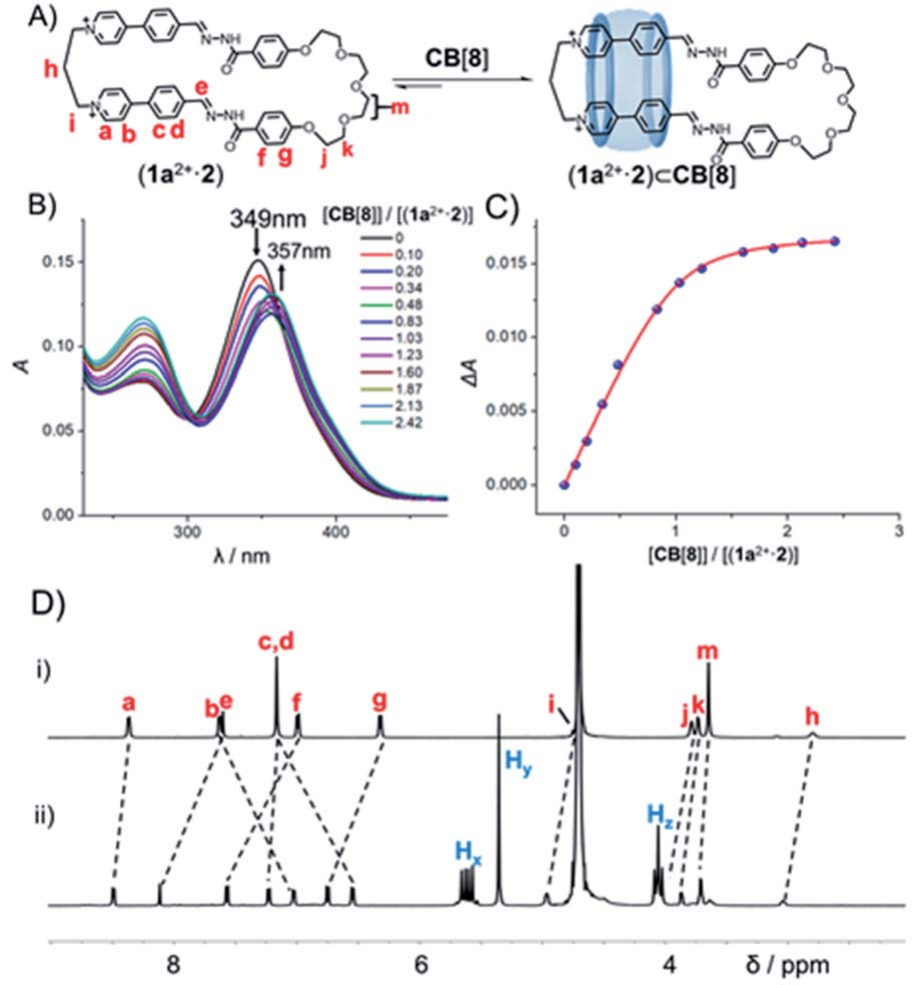
(A) The formation of (1a^2+^·2)⊂CB[8] by combining (1a^2+^·2) and CB[8] in water. (B) Partial UV/Vis absorption spectra of (1a^2+^·2)·2Cl^−^ after adding different amounts of CB[8] in H_2_O at 298 K. [(1a^2+^·2)] = 3.0 × 10^−6^ M for all spectra. (C) Plot of the changes of the absorbance intensity at *λ* = 400 nm in (B) *versus* the host/guest ratio namely [CB[8]]/[(1a^2+^·2)]. (D) Partial ^1^H NMR spectra (500 MHz, D_2_O, 298 K) of (i) (1a^2+^·2) and (ii) (1a^2+^·2)⊂CB[8], counteranions could be either Cl^−^ or Br^−^.

Upon addition of an equimolar amount of CB[8] to a D_2_O solution of a 1 : 1 mixture of 2 (2.5 mM) and each of 1x^2+^·2Br^−^ (x = a, b, c, d and e; 2.5 mM) in the presence of DCl (Fig. 1 and Scheme S8†), a set of [2]pseudorotaxanes (1x^2+^·2)⊂CB[8] (x = a, b, c, d and e) were observed to form as the predominant products. The yields of (1x^2+^·2)⊂CB[8] (x = a, b, c, d and e) (counteranions could be either Cl^−^ or Br^−^) were determined to be 80%, 74%, 88%, 90% and 85% (Fig. S62A, S63A, S64A, S65A and S66A†), respectively, calculated by using an internal standard in the corresponding ^1^H NMR samples. The structures of these [2]pseudorotaxanes were fully characterized by both ^1^H NMR spectroscopy and mass spectrometry (Fig. S6, S7, S15, S16, S25, S26, S33, S34, S42 and S43†). The ^1^H NMR spectra of both (1a^2+^·2) and (1a^2+^·2)⊂CB[8] are shown in Fig. 2D. The resonances corresponding to the protons b and c in the guest undergo remarkable upfield shifts, indicating that the CB[8] host encircles the phenylene units in the 1a^2+^ residue, driven by the hydrophobic effect. As a comparison, the phenylene units in the 2 residue are located outside the pocket of CB[8], as indicated by the downfield shifts of the corresponding protons. CB[8] chooses to reside on the phenylene units in the 1a^2+^ residue instead of those in 2, because of the cation–dipole interactions between the CB[8] host and the pyridinium cations in the 1a^2+^ residue of the macrocyclic guest. The host/guest association and dissociation occurred at a relatively slow rate on the timescale of ^1^H NMR spectroscopy, as indicated by the observation that the two protons of CB[8] become chemically inequivalent in the ^1^H NMR spectrum of (1a^2+^·2)⊂CB[8] (Fig. 2D). Addition of an equimolar amount of CB[8] to the corresponding pre-self-assembled [2]catenanes also produced the same [2]pseudorotaxane products, indicating that the corresponding [2]pseudorotaxanes (1x^2+^·2)⊂CB[8] (x = a, b, c, d and e) are thermodynamic products, instead of kinetically trapped ones. Apparently, the formation of [2]pseudorotaxanes is more favored by dipole–cation interactions resulting from CB[8], compared to the homo [2]catenanes.

In order to shed more light on the formation mechanism of the ring-in-ring pseudorotaxane, we measured the binding constants (*K*_a_) of (1a^2+^·2)⊂CB[8] by using UV–Vis titration spectroscopic experiments (Fig. 2B). A pure sample of (1a^2+^·2)·2Cl^−^ was isolated *via* counterion exchange without chromatographic purification, which is water soluble. Addition of CB[8] led to a decrease of the absorption peak of (1a^2+^·2) centered at 349 nm, while an increase of the absorption peak centered at 357 nm that corresponds to (1a^2+^·2)⊂CB[8]. *K*_a_ of (1a^2+^·2)⊂CB[8] was determined (Fig. 2C) to be 5.6(±1.1) × 10^6^ M^−1^, by fitting a 1 : 1 bind curve. Due to the fact that CB[8] is barely soluble in water, titration experiments were performed at ultra-low concentrations namely [(1a^2+^·2)] = 3.0 × 10^−3^ mM. Under such conditions, the concentrations of the compounds namely either (1a^2+^·2) or CB[8] used in titration might be pseudo-accurate, which might be responsible for the absence of a clear isosbestic point in Fig. 2B. We thus performed competitive binding experiments at higher concentrations. That is, when (1a^2+^·2), paraquat and CB[8], the concentration of each of which is 2.5 mM, were combined in D_2_O, the ^1^H NMR spectrum (Fig. S67†) indicated that the (1a^2+^·2)⊂CB[8] was formed exclusively. Such an experiment implies that (1a^2+^·2) has a stronger binding affinity, compared to paraquat whose *K*_a_ is 1.1 × 10^5^ M^−1^.^10^ A similar competitive binding experiment (Fig. S68†) also clearly demonstrated that (1a^2+^·2)⊂CB[8] is a stronger complex compared to dodecane-1,12-diammonium⊂CB[8], whose *K*_a_ is 1.1 × 10^6^ M^−1^.^11^ We thus propose that the value of *K*_a_ of (1a^2+^·2)⊂CB[8] namely 5.6(±1.1) × 10^6^ M^−1^ might still be reliable, even though not accurate.

1f^2+^·2Cl^−^, an analogue of 1x^2+^·2Br^−^ (x = a, b, c, d and e) whose polymethylene chains were replaced by a *p*-xylene unit, was synthesized. 1f^2+^·2Cl^−^ (2.5 mM) and 2 (2.5 mM) were combined in D_2_O in the presence of a catalytic amount of DCl (Fig. 4 and Scheme S9†). The solution was heated at 60 °C for 8 h. Both ^1^H NMR spectroscopy and mass spectrometry (Fig. 4D, S46 and S47†) demonstrated that a [2]catenane (1f^2+^·2)_2_·4Cl^−^ was produced as the predominant product. The NMR yield was calculated to be 94% (Fig. S60†). This yield is higher than those of the catenane containing polymethylene chains including (1c^2+^·2)_2_ and (1d^2+^·2)_2_, probably because the ring (1f^2+^·2) contains a more rigid *p*-xylene unit. This linker affords the ring a more preorganized cavity that favours catenation to a more significant extent.

Upon the addition of 2 equiv. of CB[8] to a D_2_O solution of (1f^2+^·2)_2_ (Fig. 4 and Scheme S10†), the resonances corresponding to the catenane completely disappeared, while a new set of resonances was observed after heating the solution at 60 °C for 24 h (Fig. S57D†). Surprisingly, both the ^1^H NMR spectrum (Fig. 4B) and mass spectrum (Fig. S52†) indicated that a [3]pseudorotaxane namely (1f^2+^·2·1f^2+^·2)⊂2CB[8] was self-assembled, containing two CB[8] rings that encircle an ultra-large macrocycle (1f^2+^·2·1f^2+^·2), which condensed in a [2 + 2] manner. The yield of (1f^2+^·2·1f^2+^·2)⊂2CB[8] was determined to be 68%, by using an internal standard in the ^1^H NMR sample (Fig. S61†). It is noteworthy that a putative [2]pseudorotaxane (1f^2+^·2·1f^2+^·2)⊂CB[8] containing only one CB[8] was not observed during the self-assembly. The binding of two CB[8] rings on the (1f^2+^·2·1f^2+^·2) ring is positively cooperative. This proposition was supported by the observation that, addition of one equiv. of CB[8] to a D_2_O solution of (1f^2+^·2)_2_ led to a 1 : 1 mixture of the unreacted [2]catenane and the [3]pseudorotaxane (Fig. S57C†). It is likely that upon recognition of the first CB[8] ring, the (1f^2+^·2·1f^2+^·2) guest became more preorganized to accommodate the second ring.

Adamantan-1-ol,^12^ a competitive guest binding with CB[8], was added into the aqueous solution of (1f^2+^·2·1f^2+^·2)⊂2CB[8] (Fig. 4 and S58†). After heating the corresponding solution at 60 °C for 48 h, the ^1^H NMR spectrum (Fig. S58F†) demonstrated that (1f^2+^·2·1f^2+^·2)⊂2CB[8] was almost completely transformed into adamantan-1-ol⊂CB[8], leading to recovery of the [2]catenane (1f^2+^·2)_2_. This observation indicated that (i) adamantan-1-ol represents a better guest compared to (1f^2+^·2·1f^2+^·2), with respect to the CB[8] host; (ii) without the external templation of the CB[8] ring, the ultra-large ring (1f^2+^·2·1f^2+^·2) is no longer a thermodynamically stable compound in water compared to its [2]catenane isomer namely (1f^2+^·2)_2_.

Because (1f^2+^·2·1f^2+^·2) can not be isolated, accurate determination of *K*_a1_ and *K*_a2_ of (1f^2+^·2·1f^2+^·2)⊂2CB[8] was difficult or impossible. However, we could qualitatively evaluate *K*_a2_ by using competitive experiments. Upon heating a 2 : 1 : 2 mixture of CB[8], the [2]catenane (1f^2+^·2)_2_ and the ring (1a^2+^·2) (Fig. S69†) for 24 h at 60 °C, the [2]pseudorotaxane (1a^2+^·2)⊂CB[8] was self-assembled selectively, leaving (1f^2+^·2)_2_ unreacted in water. As a comparison, heating a 1 : 2 : 2 mixture of the [2]catenane (1f^2+^·2)_2_, paraquat, and CB[8] yielded (1f^2+^·2·1f^2+^·2)⊂2CB[8] as the major product, accompanied by a small amount of paraquat⊂CB[8] as the minor product (Fig. S70†). Such an experiment indicated that *K*_a2_ might be comparable to or a little larger than the *K*_a_ of paraquat⊂CB[8], namely 1.1 × 10^5^ M^−1^, while smaller than the *K*_a_ of (1a^2+^·2)⊂CB[8].

**Fig. 3:**
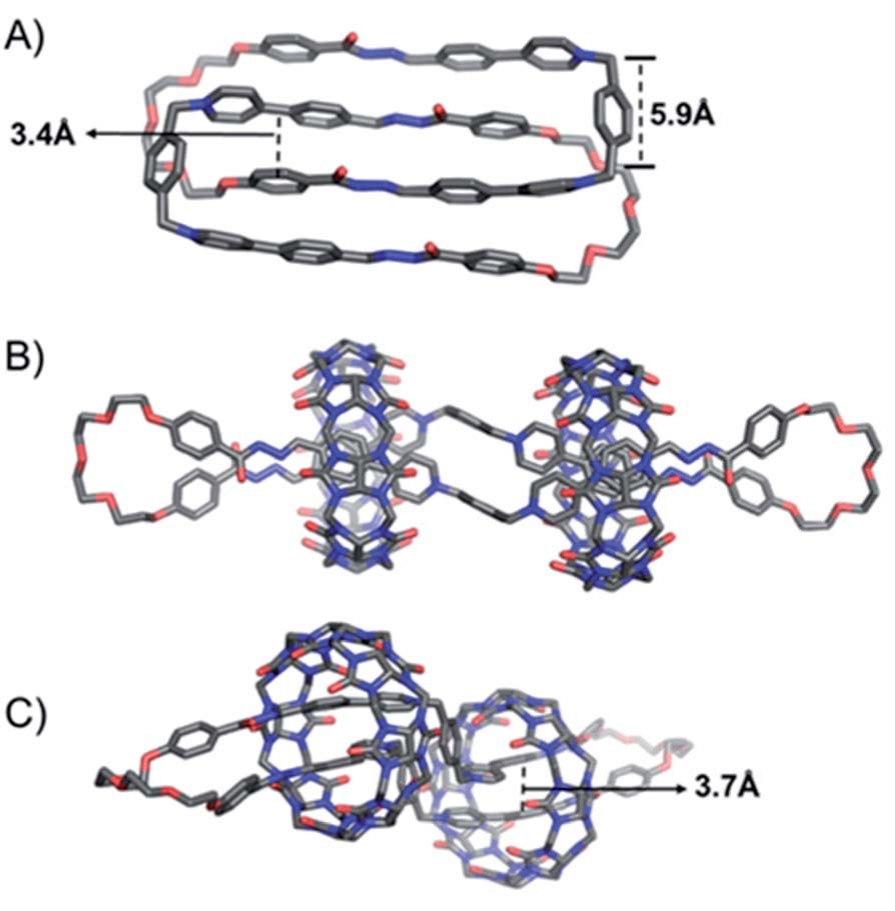
Solid-state structures of (A) (1f^2+^·2)_2_, (B) (1f^2+^·2·1f^2+^·2)⊂2CB[8] (side view), and (C) (1f^2+^·2·1f^2+^·2)⊂2CB[8] (rear view), obtained from single-crystal X-ray diffraction analysis. Oxygen atoms, red; nitrogen, blue; carbon, gray. Hydrogen atoms, counteranions, and disordered solvent molecules are omitted for clarity. Some of the close contacts are labeled with dashed lines, indicating the occurrence of intramolecular interactions such as π–π interactions.

Single crystals of the [2]catenane (1f^2+^·2)_2_ and the [3]pseudorotaxane (1f^2+^·2·1f^2+^·2)⊂2CB[8] were obtained by either slow vapor diffusion of THF into the aqueous solution of (1f^2+^·2)_2_ (Fig. 3A), or slow evaporation of the aqueous solution of (1f^2+^·2·1f^2+^·2)⊂2CB[8] (Fig. 3B and C), which provided unambiguous evidence to convince their architectures. In the case of [2]catenane (1f^2+^·2)_2_, the cavity of each of the two mutually mechanically interlocked rings is almost fully occupied by another ring, leading to a variety of intercomponent close contacts. For example, each of the pyridinium building blocks in one ring undergoes π–π interactions with an adjacent phenylene unit in another ring, as inferred from their short interplane distances, *i.e.*, around 3.4 Å (Fig. 3A). Close contacts also indicate the occurrence of CH⋯O hydrogen bonding and CH–π interactions. In the case of the ring-in-rings complex namely the [3]pseudorotaxane (1f^2+^·2·1f^2+^·2)⊂2CB[8] (Fig. 3B and C), each of the 1f^2+^ residues adopts a different conformation compared to that in the [2]catenane (1f^2+^·2)_2_, *i.e.*, in the former system, the two 4-phenylpyridinium units orient in different directions with respect to the central *p*-xylene unit. The two methylene units in the 1f^2+^ residue are separated by 5.9 Å (Fig. 3A). This distance implies that the putative [1 + 1] ring, namely (1f^2+^·2), would be too large to fit within the cavity of CB[8]. As a consequence, the putative [2]pseudorotaxane (1f^2+^·2)⊂CB[8] can not form. Each of the CB[8] rings encircles two 4-phenylpyridinium units belonging to two different 1f^2+^ residues. Such behavior favors the formation of the ultra-large macrocycle in a [2 + 2] manner. Within the cavity of the CB[8] ring, two phenylene units undergo π–π interactions, as inferred from the close contact namely 3.7 Å (Fig. 3C). The two pyridinium units, as a comparison, exhibit a displaced parallel orientation, which helps to avoid pyridinium–pyridinium electrostatic repulsion while strengthening ion–dipole and CH–O interactions between the cationic pyridinium units and the carbonyl groups in CB[8].

**Fig. 4:**
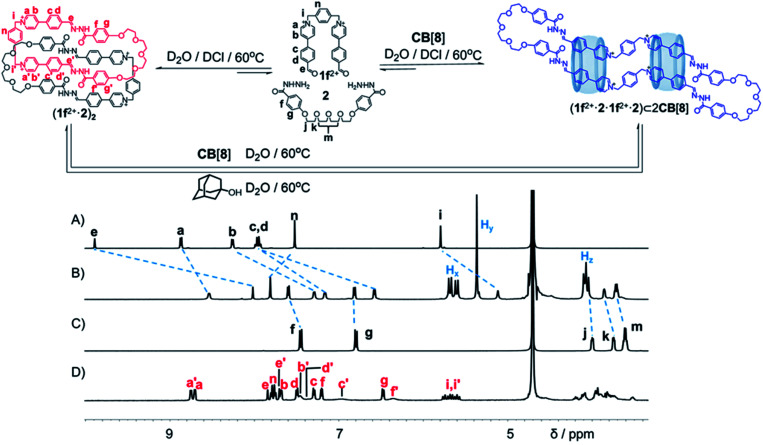
Structural formulae of a [2]catenane (1f^2+^·2)_2_ and a [3]pseudorotaxane (1f^2+^·2·1f^2+^·2)⊂2CB[8], by condensing a dicationic bisaldehyde 1f^2+^ and a bishydrazide 2 in water, in the absence and presence of CB[8] respectively. Partial ^1^H NMR spectra (500 MHz, D_2_O, 298 K) of (A) 1f^2+^, (B) (1f^2+^·2·1f^2+^·2)⊂2CB[8], (C) 2 and (D) (1f^2+^·2)_2_. The assignment of each resonance was made using the corresponding two-dimensional NMR spectra shown in the ESI.† Charges are balanced by Cl^−^ counteranions, which are omitted here for the sake of clarity.

## Conclusion

In summary, by combining the corresponding bishydrazide and a set of cationic bisaldehydes bearing polymethylene chains in aqueous media, a series of [1 + 1] macrocycles were self-assembled, accompanied with the corresponding dimers namely [2]catenanes. The product distribution was determined by the length of the polymethylene chains in the bisaldehyde precursors. That is, the longer chains favor the formation of [2]catenanes, while the shorter ones favor the macrocycles more. Upon addition of CB[8], the macrocycles form a set of [2]pseudorotaxanes, driving the catenane/macrocycle equilibria to the side of macrocycles. When the polymethylene units in the bisaldehyde compounds are replaced by a *p*-xylene unit, the putative [1 + 1] condensed ring is too large to fit within the ring of CB[8]. Instead, an ultra-large macrocycle was self-assembled in a [2 + 2] manner, which was encircled by two CB[8] rings that act as the external templates. The framework of the ultra-large ring contains more than one hundred atoms, whose synthesis would be thermodynamically disfavored in the absence of the external template, namely CB[8]. We envision that the success in formation of a ring-in-rings complex would be taken advantage of in the synthesis of more complex architectures, such as Borromean rings. Such trials are ongoing in our laboratory.

## Data availability

Data for this paper, including the synthesis, structural characterization are available at ESI.†

## Author contributions

**Conceptualization**: Hao Li, Qiong Chen. **Data curation**: Qiong Chen, Ye Lei. **Formal analysis**: Qiong Chen, Hao Li. **Investigation**: Qiong Chen, Ye Lei. **Methodology**: Qiong Chen, Guangcheng Wu, Yuanjiang Pan. **Project administration**: Qiong Chen, Qing Li. **Resources**: Hao Li. **Software**: Qiong Chen. **Supervision**: Qiong Chen, Hao Li. **Validation**: Qiong Chen, Ye Lei. **Roles/Writing – original draft**: Qiong Chen. **Writing – review & editing**: Hao Li.

## Conflicts of interest

There are no conflicts to declare.

## Supplementary Material

SC-013-D1SC06236K-s001

SC-013-D1SC06236K-s002
