# Supplementary material for: Ultramacrocyclization in water via external templation
Source: Chem Sci. 2022 Jan 5;13(3):798–803. doi: 10.1039/d1sc06236k (PMC8768864; doi:10.1039/d1sc06236k)
Supplement: SC-013-D1SC06236K-s001 [file SC-013-D1SC06236K-s001.pdf]

Electronic Supplementary Material (ESI) for Chemical Science.
This journal is © The Royal Society of Chemistry 2021

# Ultramacrocyclization in water via External Templation

# Supporting Information

### Table of Contents

1. General Methods ........................................................................................2

2. Synthetic Procedures.....................................................................................3

3. Characterization of the macrocycles, catenanes and pseudorotaxanes ....13

4. Interconversion between (**1f**$^{2+}$·**2**)$_2$ and (**1f**$^{2+}$·**2**·**1f**$^{2+}$·**2**)⊂2**CB**[**8**] ...................68

5. The process of generating (**1f**$^{2+}$·**2**·**1f**$^{2+}$·**2**)⊂2**CB**[**8**] by means of one-pot procedure..............................................................................................71

6. Yields of the condensation of **2** and a series of biscationic dialdehydes ....72

7. Competitive experiments ..............................................................................79

8. UV-Vis Spectroscopic Analysis.......................................................................83

9. X-ray Crystallography ....................................................................................86

10. References....................................................................................................93

## 1. General Methods

All reagents and solvents were purchased from commercial sources and used without further purification. Compounds **3**[1] were prepared according to a reported procedure. Manipulations were performed under a normal laboratory atmosphere unless otherwise noted. Nuclear magnetic resonance (NMR) spectra were recorded at ambient temperature using Bruker AVANCE III 400, Bruker AVANCE III 500, or Agilent DD2 600 spectrometers, with working frequencies of 400/500/600 and 100/125/150 MHz for $^{1}H$ and $^{13}C$, respectively. Chemical shifts are reported in ppm relative to the residual internal non deuterated solvent signals ($D_2O$: $\delta$ = 4.79 ppm, DMSO-$d_6$: $\delta$ = 2.50 ppm, $CD_3CN$: $\delta$ = 1.94 ppm). High-resolution mass spectra (HRMS) were measured by using a SHIMADZU liquid chromatograph mass spectrometry ion trap time of flight (LCMS-IT-TOF) instrument. X-ray crystallographic data were collected on a Bruker D8 Venture. UV/Vis spectra were recorded on a Agilent Cary 5000 instrument.

## 2. Synthetic Procedures

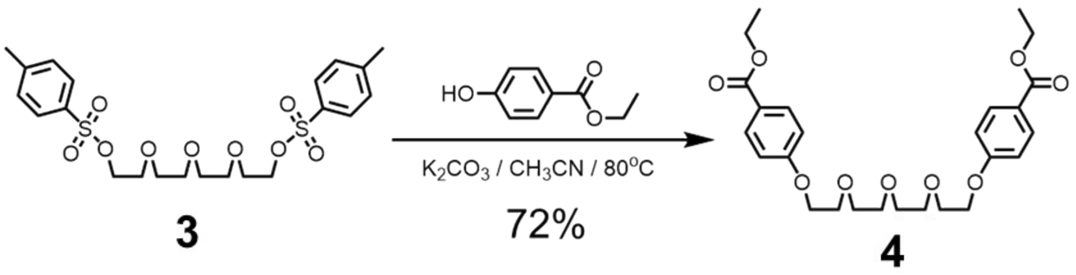

**Scheme S1.** Synthesis of **4**.

**4**: **3** (2mmol, 1.0g) and ethylparaben (4.4mmol, 731.1mg) were dissolved in 40ml anhydrous acetonitrile placed in a 100 ml round bottom flask. In a nitrogen atmosphere, $K_2CO_3$ (8.8mmol, 1.2g) was then added into the flask. The mixture was heated at 80 °C for 12 h. After completion of reaction, the solution was cooled to room temperature. The precipitates were removed by filtration. The filtrate was collected and most of the solvent was removed under vacuum. The residue was poured into water, which was then extracted with ethyl acetate. The resulting organic layer was combined, and washed with water (3 x 50 mL), dried over anhydrous $Na_2SO_4$, and then concentrated to give the crude product. Purification by flash column chromatography (petroleum ether/ethyl acetate (4:1); silica gel, 200-300 mesh) yielded the white solid-state product **4** (686.2 mg, 70 %). **$^1$H NMR** (400 MHz, $CDCl_3$): δ = 7.99 (d, J=8.0Hz, 4H), 6.93 (d, J=8Hz, 4H), 4.35 (q, J=8.0Hz, 4H), 4.17 (t, J=4.0Hz, 4H ), 3.88 (t, J=4.0Hz, 4H), 3.72(m, 8H), 1.39 (t, J=8.0Hz, 6H). **$^{13}$C NMR** (400 MHz, $CDCl_3$): δ = 166.3, 162.5, 131.5, 123.1, 114.1, 70.9, 70.7, 69.6, 67.5, 60.6, 14.4. **HRMS**: *m/z* calculated for $C_{24}H_{30}O_8Na^+$ ($[M + Na]^+$): 469.1817; found: 469.1833.

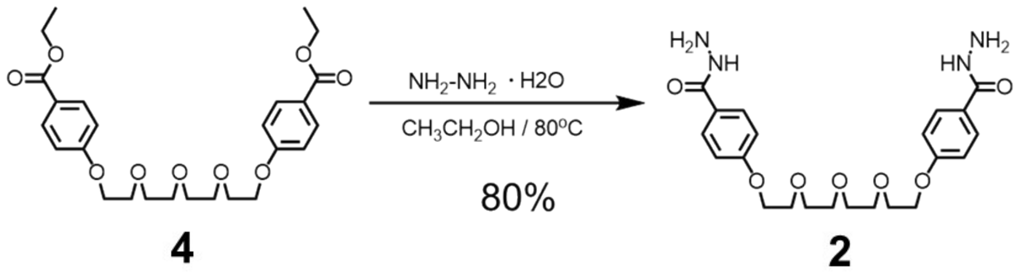


**Scheme S2.** Synthesis of **2**.

**2**: In a 50mL round bottom flask, **4** (490.2mg, 1mmol) was dissolved in 15ml ethyl alcohol. Hydrazine hydrate (1.0g, 200mmol) was then added into the flask. The mixture was refluxed for 12 h at 80 °C. After completion of reaction, the solution was cooled to room temperature. The solvent was removed under vacuum to give the crude product. In order to remove residual hydrazine hydrate, the crude product was washed with ethanol for several times, yielding the orange-red solid-state product **2c** (369.6 mg, 80 %). **$^1$H NMR** (400 MHz, $D_2O$): δ = 7.45 (d, J=8.0Hz, 4H), 6.80 (d, J=8.0Hz, 4H), 4.00 (t, J=4.0Hz, 4H), 3.74 (t, J=4.0Hz, 4H), 3.61(m, 8H), 3.25 (s, 6H). **$^{13}$C NMR** (400 MHz, $D_2O$): δ = 169.0, 160.9, 128.8, 124.3, 114.3, 69.7, 69.6, 68.8, 67.1. **HRMS**: *m/z* calculated for $C_{22}H_{30}N_4O_7Na^+$ ([M + Na]$^+$): 485.2012; found: 485.2000.

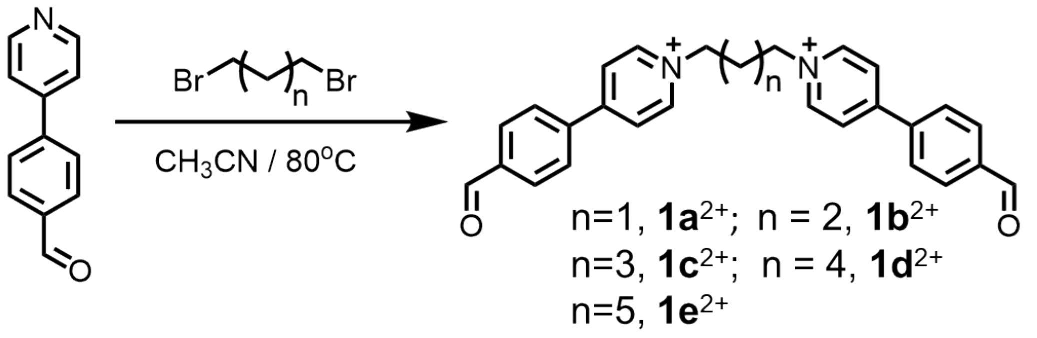


**Scheme S3.** Synthesis of **1a**$^{2+}$, **1b**$^{2+}$, **1c**$^{2+}$, **1d**$^{2+}$and **1e**$^{2+}$. Charges are balanced by bromide counteranions, which are omitted here for the sake of clarity.

**1a**$^{2+}$·2Br$^-$: 1,3-Dibromopropane (202.0 mg, 1.0 mmol) and 4-(4-formylphenyl)pyridine (457.5 mg, 2.5 mmol) were dissolved in 15 ml dry MeCN placed in a 50ml round bottom flask. The reaction mixture was stirred at 80°C for 24 h, during which a white precipitate was formed. After cooling to room temperature, the precipitate was collected via filtration, which was washed with ethyl acetate to give a white solid **1a**$^{2+}$**·**2Br$^-$ (537.7 mg, 95%). **$^1$H NMR** (600 MHz, $D_2O$): δ = 9.97 (s, 2H), 8.90 (d, J=6.0Hz, 4H), 8.32 (d, J=6.0Hz, 4H), 8.00 (d, J=6.0Hz, 4H), 7.95 (d, J=6.0Hz, 4H), 4.87 (t, J=6.0Hz, 4H), 2.90 (m, 2H). **$^{13}$C NMR** (400 MHz, $D_2O$): δ = 195.3, 155.7, 144.5, 139.1, 137.6, 130.7, 128.7, 126.1, 58.1, 31.0. **HRMS**: *m/z* calculated for $C_{27}H_{24}N_2O_2^{2+}$ ([M – 2Br]$^{2+}$): 204.0913; found: 204.0925.

**1b**$^{2+}$·2Br$^-$,**1c**$^{2+}$·2Br$^-$,**1d**$^{2+}$·2Br$^-$, and **1e**$^{2+}$·2Br$^-$ were synthesized via similar procedures, using different dibromo precursors.

**1b**$^{2+}$·2Br$^-$: The yield of a white solid **1b**$^{2+}$·2Br$^-$ is 98 %. **$^1$H NMR** (500 MHz, $D_2O$): δ = 9.95 (s, 2H), 8.80 (d, J=10.0Hz, 4H), 8.27 (d, J=10.0Hz, 4H), 8.01 (d, J=8.0Hz, 4H), 7.96 (d, J=8.0Hz, 4H), 4.61 (m, 4H), 2.10 (m, 4H). **$^{13}$C NMR** (400 MHz, $D_2O$): δ = 195.4, 155.5, 144.3, 139.5, 137.5, 130.7, 128.7, 125.9, 60.2, 27.2. **HRMS**: *m/z* calculated for $C_{28}H_{26}N_2O_2^{2+}$ ([M – 2Br]$^{2+}$): 211.0992; found: 211.0994.

**1c**$^{2+}$·2Br$^-$: The yield of a light yellow solid **1c**$^{2+}$·2Br$^-$ is 85 %. **$^1$H NMR** (500 MHz, $D_2O$): δ = 9.88 (s, 2H), 8.74 (d, J=10.0Hz, 4H), 8.19 (d, J=10.0Hz, 4H), 7.85 (d, J=8.0Hz, 4H),

7.81 (d, J=8.0Hz, 4H), 4.57 (t, J=6.5Hz, 4H), 2.03 (m, 4H), 1.01 (m, 2H). **$^{13}$C NMR** (400 MHz, $D_2O$): δ = 195.3, 154.8, 144.4, 138.9, 137.3, 130.6, 128.4, 125.4, 60.6, 29.0, 20.9. **HRMS**: *m/z* calculated for $C_{29}H_{28}N_2O_2^{2+}$ ([M – 2Br]$^{2+}$): 218.1070; found: 218.1077.

**1d**$^{2+}$·2Br$^-$: The yield of a white solid is **1d**$^{2+}$·2Br$^-$ is 88 %. **$^1$H NMR** (400 MHz, $D_2O$): δ = 9.88 (s, 2H), 8.73 (d, J=8.0Hz, 4H), 8.16 (d, J=8.0Hz, 4H), 7.92 (d, J=8.0Hz, 4H), 7.86 (d, J=8.0Hz, 4H), 4.48 (t, J=6.8Hz, 4H), 1.92 (m, 4H), 1.21 (m, 4H). **$^{13}$C NMR** (400 MHz, $D_2O$): δ = 195.4, 155.1, 144.3, 139.4, 137.5, 130.7, 128.6, 125.6, 61.3, 29.8, 25.0. **HRMS**: *m/z* calculated for $C_{30}H_{30}N_2O_2^{2+}$ ([M – 2Br]$^{2+}$): 225.1148; found: 225.1159.

**1e**$^{2+}$·2Br$^-$: The yield of a white solid **1e**$^{2+}$·2Br$^-$ is 86 %. **$^1$H NMR** (400 MHz, $D_2O$): δ = 9.88 (s, 2H), 8.74 (d, J=8.0Hz, 4H), 8.17 (d, 4H), 7.88 (d, J=8.0Hz, 8H ), 7.84 (d, J=8.0Hz, 8H ), 4.48 (t, J=7.2Hz, 4H), 1.86 (m, 4H), 1.30 (m, 2H), 1.23 (m, 4H). **$^{13}$C NMR** (400 MHz, $D_2O$): δ = 195.1, 154.6, 144.3, 139.2, 137.3, 130.6, 128.5, 125.5, 61.2, 30.0, 27.2, 24.6. **HRMS**: *m/z* calculated for $C_{31}H_{32}N_2O_2^{2+}$ ([M – 2Br]$^{2+}$): 232.1226; found: 232.1226.

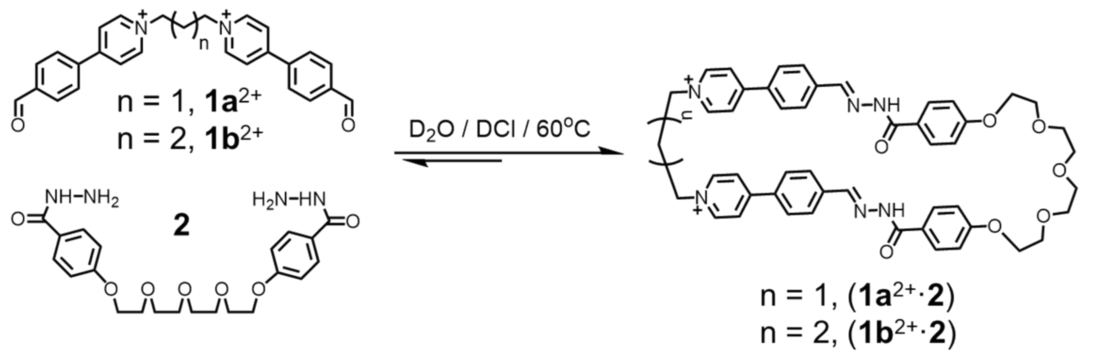


**Scheme S5.** Self-assembly of (**1a**$^{2+}$·**2**) and (**1b**$^{2+}$·**2**) (counteranions could be either $Cl^-$ or $Br^-$).

We combined **2** (4.62 mg, 0.01 mmol) with each of the dialdehyde compounds either **1a**$^{2+}$·$2Br^-$ or **1b**$^{2+}$·$2Br^-$ (0.01 mmol), in $D_2O$ (4 mL) in the presence of catalytic amount of DCl (10 μL). After heating the corresponding solutions at 60 $^{o}$C for 8 h, the $^1$H NMR and mass spectra of the corresponding solutions were recorded. It was demonstrated that the macrocycles (**1a**$^{2+}$·**2**) and (**1b**$^{2+}$·**2**), whose counteranions could be either $Cl^-$ or $Br^-$, were produced as the major products, which were fully characterized in $D_2O$ by $^1$H NMR spectroscopy, 2D NMR spectroscopy , as well as mass spectrometry (see Figure S1-5, S10-14).

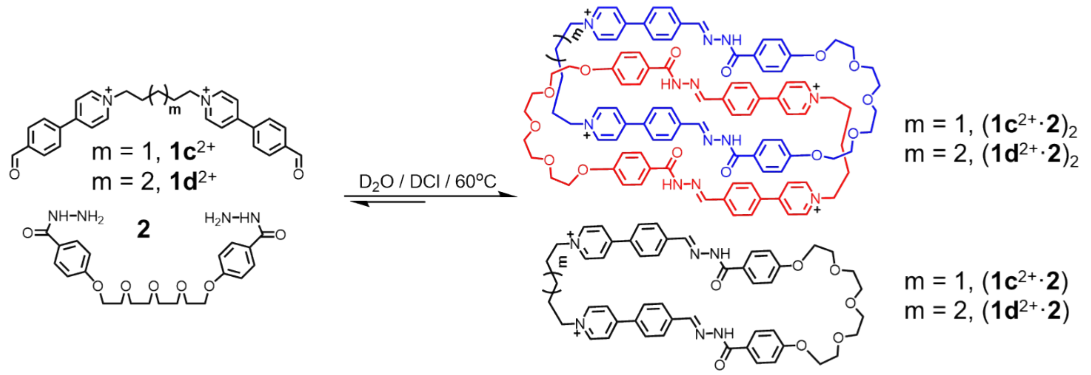

**Scheme S6.** Self-assembly of ($\mathbf{1c}^{2+}\cdot\mathbf{2}$), ($\mathbf{1c}^{2+}\cdot\mathbf{2}$)$_2$, ($\mathbf{1d}^{2+}\cdot\mathbf{2}$) and ($\mathbf{1d}^{2+}\cdot\mathbf{2}$)$_2$ (counteranions could be either $Cl^-$ or $Br^-$).

We combined **2** (4.62 mg, 0.01 mmol) with each of the dialdehyde compounds either $\mathbf{1c}^{2+}\cdot 2Br^-$ or $\mathbf{1d}^{2+}\cdot 2Br^-$ (0.01 mmol) in a 1:1 ratio, in $D_2O$ (4 mL) in the presence of catalytic amount of DCl (10 μL). After heating the corresponding solutions at 60 °C for 8 h, the $^1H$ NMR and mass spectra of the corresponding solutions were recorded. It was demonstrated that [2]catenanes ($\mathbf{1c}^{2+}\cdot\mathbf{2}$)$_2$, ($\mathbf{1d}^{2+}\cdot\mathbf{2}$)$_2$ and macrocycles ($\mathbf{1c}^{2+}\cdot\mathbf{2}$), ($\mathbf{1d}^{2+}\cdot\mathbf{2}$) (counteranions could be either $Cl^-$ or $Br^-$) were produced at the same time, which were fully characterized in $D_2O$ by $^1H$ NMR spectroscopy, 2D NMR spectroscopy , as well as mass spectrometry (see Figure S19-24, S29-32).

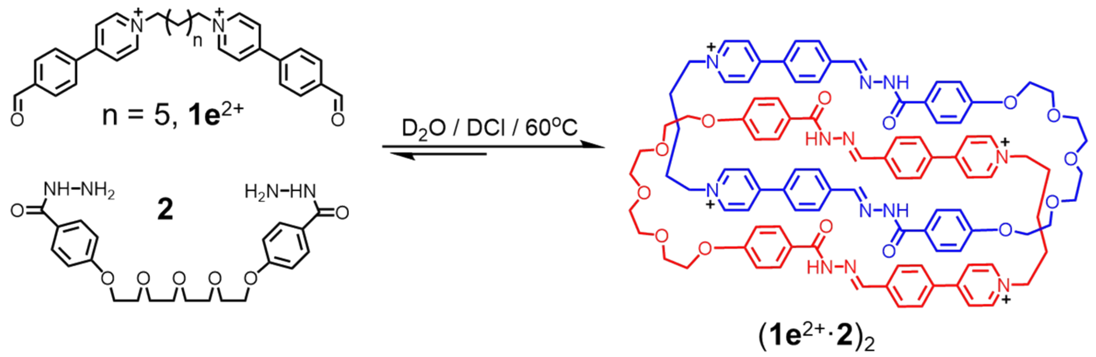


**Scheme S7.** Self-assembly of (**1e**$^{2+}$·**2**)$_2$ (counteranions could be either Cl⁻ or Br⁻).

We combined **2** (4.62 mg, 0.01 mmol) with the dialdehyde compound **1e**$^{2+}$·2Br⁻ (0.01 mmol) in a 1:1 ratio, in $D_2O$ (4 mL) in the presence of catalytic amount of DCl (10 μL). After heating the corresponding solution at 60 °C for 8 h, the $^1$H NMR and mass spectra of the corresponding solution was recorded. It was demonstrated that the [2]catenane (**1e**$^{2+}$·**2**)$_2$ (counteranions could be either Cl⁻ or Br⁻) were produced as the major product, which were fully characterized in $D_2O$ by $^1$H NMR spectroscopy, 2D NMR spectroscopy , as well as mass spectrometry (see Figure S37-41).

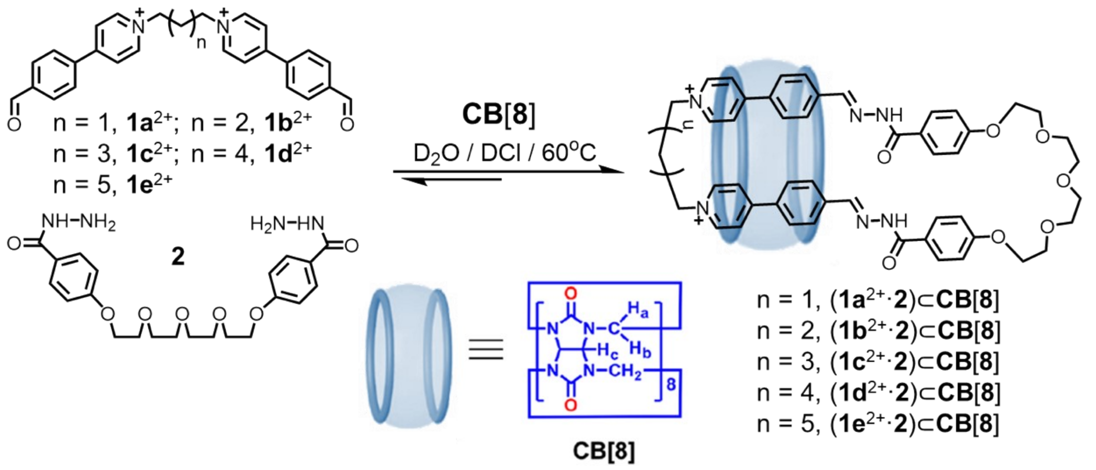


**Scheme S8.** Self-assembly of (**1x**$^{2+}$·**2c**)⊂**CB**[8] (**x** = **a**, **b**, **c**, **d** and **e**) (counteranions could be either $Cl^-$ or $Br^-$).

A 1:1:1 mixture of **2** (4.62 mg, 0.01 mmol), each of the dialdehyde compounds **1x**$^{2+}$·2$Br^-$ (**x** = **a**, **b**, **c**, **d** and **e**) (0.01 mmol) and **CB**[8] (0.01 mmol) were combined and dissolved in $D_2O$ (4 mL) in the presence of catalytic amount of DCl (10 μL). After heating the corresponding solutions at 60 °C for 8 h, the $^1$H NMR and mass spectra of the corresponding solutions were recorded. It was demonstrated that the [2]pseudorotaxanes (**1x**$^{2+}$·**2c**)⊂**CB**[8] (counteranions could be either $Cl^-$ or $Br^-$) were produced as the major products, which were fully characterized in $D_2O$ by $^1$H NMR spectroscopy, 2D NMR spectroscopy , as well as mass spectrometry (see Figure S6-9, S15-18, S25-28, S33-36, S42-45).

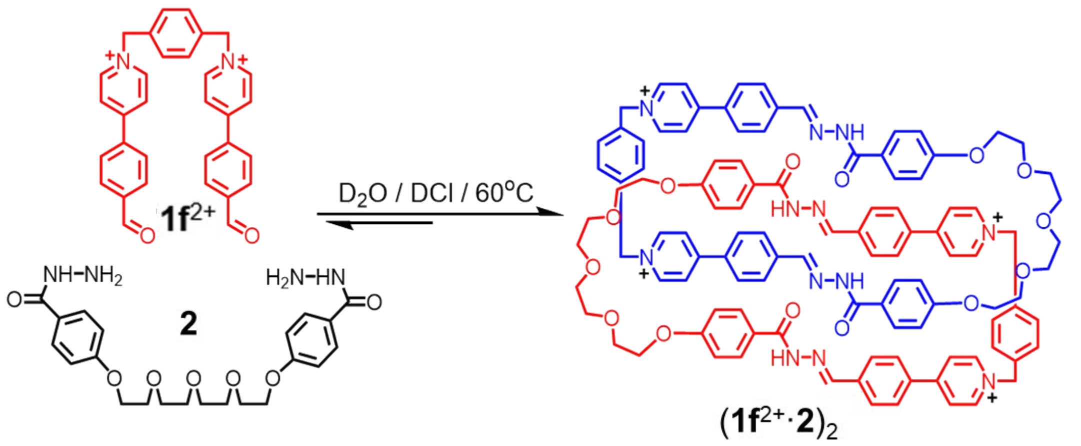


**Scheme S9.** Self-assembly of (**1f**$^{2+}$·**2**)$_2$. Charges are balanced by chloride counteranions, which are omitted here for the sake of clarity.

A 1:1 mixture of the dialdehyde compound **1f**$^{2+}$·2Cl$^{–}$ (5.40 mg, 0.01 mmol) and the dihydrazine linker **2** (0.01 mmol) were combined in $D_2O$ (4 mL) in the presence of catalytic amount of DCl (10 μL). After heating the corresponding solutions at 60 $^{o}$C for 8 h, the $^{1}$H NMR (Figure S47) and mass spectra (Figure S46) of the corresponding solution were recorded. To our delight, the corresponding $^{1}$H NMR spectrum indicated the formation of a thermodynamically stable product, a homo [2]catenanes (**1f**$^{2+}$·**2**)$_2$·4Cl$^{–}$. The structure of (**1f**$^{2+}$·**2**)$_2$·4Cl$^{–}$ was further convinced by $^{1}$H−$^{1}$H COSY spectrum (Figure S49), $^{1}$H−$^{1}$H NOESY spectrum (Figure S50) and DOSY spectrum (Figure S51).

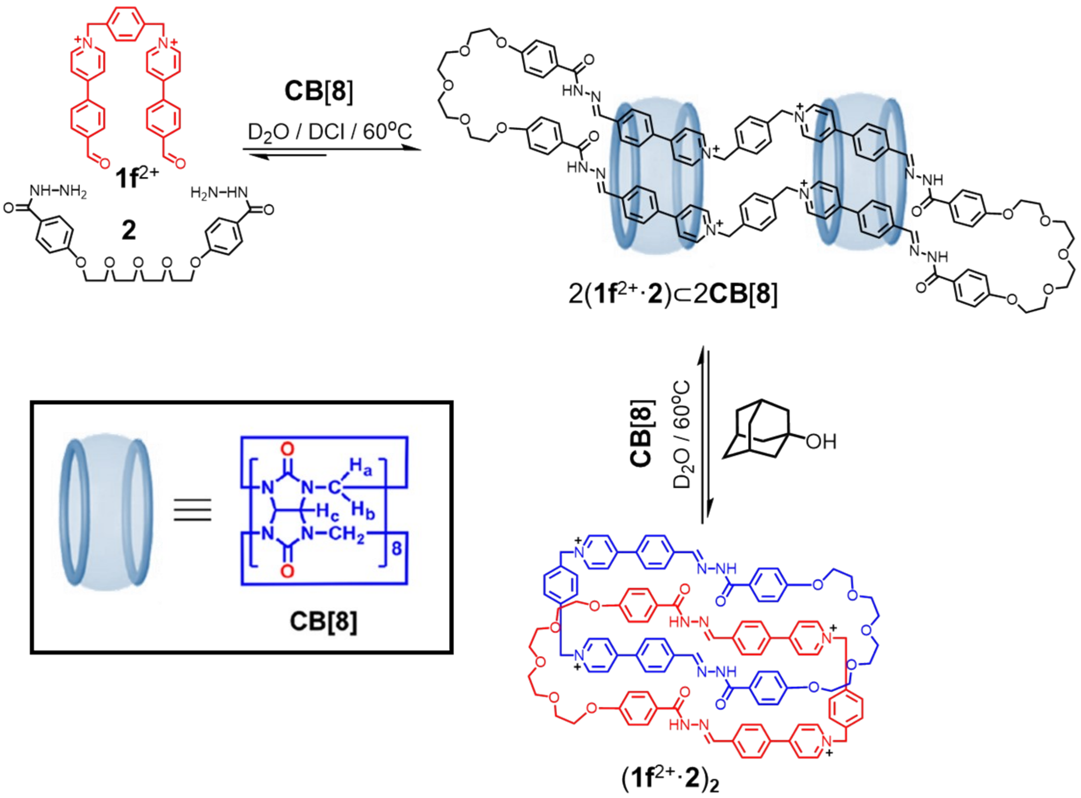


**Scheme S10.** Self-assembly of (**1f**$^{2+}$·**2**·**1f**$^{2+}$·**2**) ⊂2**CB**[8]. Charges are balanced by chloride counteranions.

A 1:1:1 mixture of the dihydrazine **2** (4.62 mg, 0.01 mmol), the dialdehyde compound **1f**$^{2+}$·2Cl$^{-}$ (5.40 mg, 0.01 mmol) and **CB**[8] (0.01 mmol) were combined and dissolved in water (4 mL) in the presence of catalytic amount of DCl (10 μL). After heating the corresponding solutions at 60 °C for 8 h, the $^{1}$H NMR and mass spectra of the corresponding solutions were recorded. It was demonstrated that the [3]pseudorotaxane (**1f**$^{2+}$·**2**·**1f**$^{2+}$·**2**) ⊂2**CB**[8] was produced as the major product, which were fully characterized in $D_2O$ by $^{1}$H NMR spectroscopy, 2D NMR spectroscopy , as well as mass spectrometry (see Figure S52-55).

Upon removal of **CB**[8] by adding excess amount of adamantan-1-ol, the [3]pseudorotaxane (**1f**$^{2+}$·**2**·**1f**$^{2+}$·**2**) ⊂2**CB**[8] could be transformed back to the [2]catenane (**1f**$^{2+}$·**2**)$_2$, accompanied with a complex namely adamantan-1-ol ⊂**CB**[8].

# 3. Characterization of the macrocycles, catenanes and pseudorotaxanes

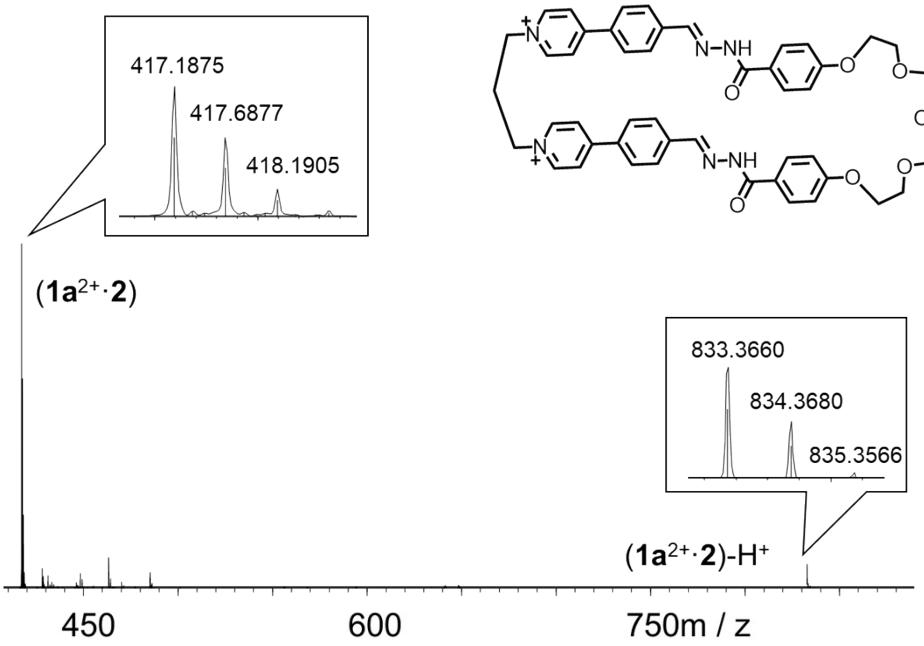


**Figure S1.** High-resolution LCMS-IT-TOF of ($\mathbf{1a}^{2+}\cdot\mathbf{2}$). Counteranions could be either $Cl^-$ or $Br^-$. The signals labeled in the spectrum correspond to molecular cations that contain two and one positive charges, respectively. *m/z* $[(\mathbf{1a}^{2+}\cdot\mathbf{2})]^{2+}$ calculated for $C_{49}H_{50}N_6O_7^{2+}$: 417.1865; found: 417.1875. $[(\mathbf{1a}^{2+}\cdot\mathbf{2}) - H^+]^+$ calculated for $C_{49}H_{49}N_6O_7^{+}$: 833.3657; found: 833.3660.

;

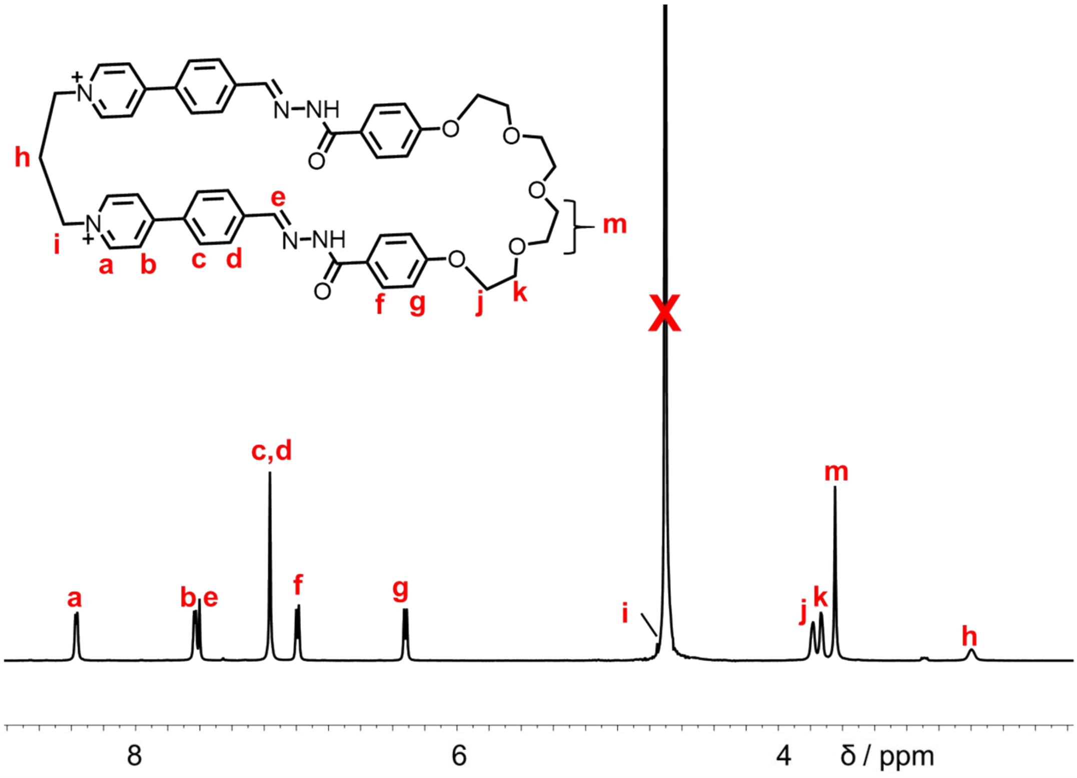


**Figure S2.** Partial $^1$H NMR spectrum of (**1a**$^{2+}$·**2**) (500 MHz, $D_2O$, 298 K). Counteranions could be either $Cl^-$ or $Br^-$.

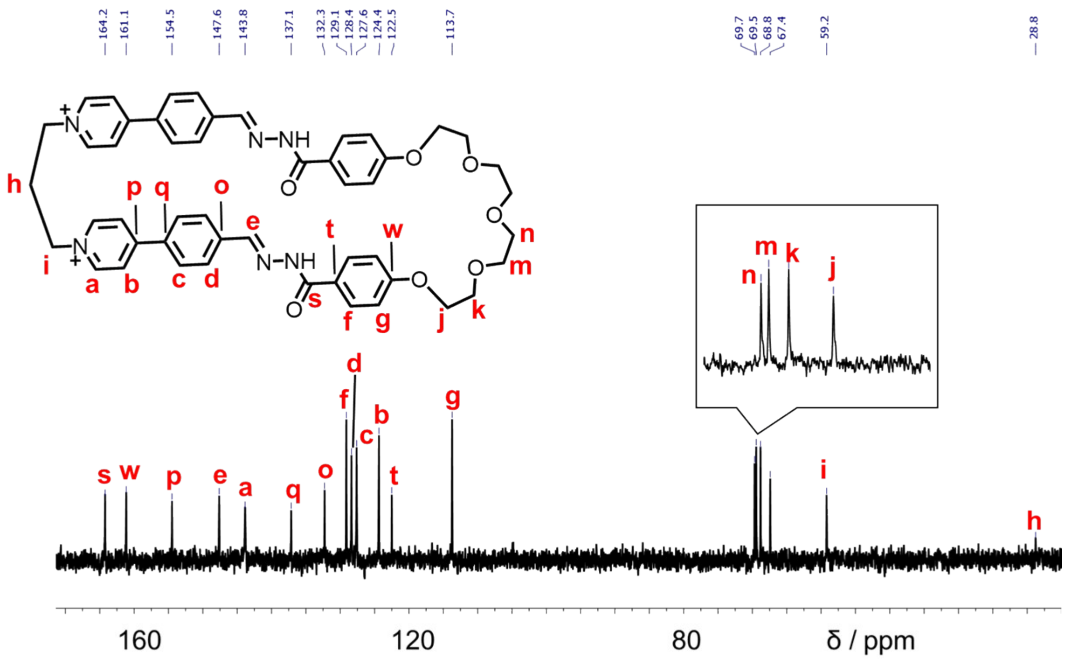


**Figure S3.** $^{13}C$ NMR spectrum (100 M Hz, 298 K, $D_2O$) of (**1a**$^{2+}$·**2**). Counteranions could be either $Cl^–$ or $Br^–$.

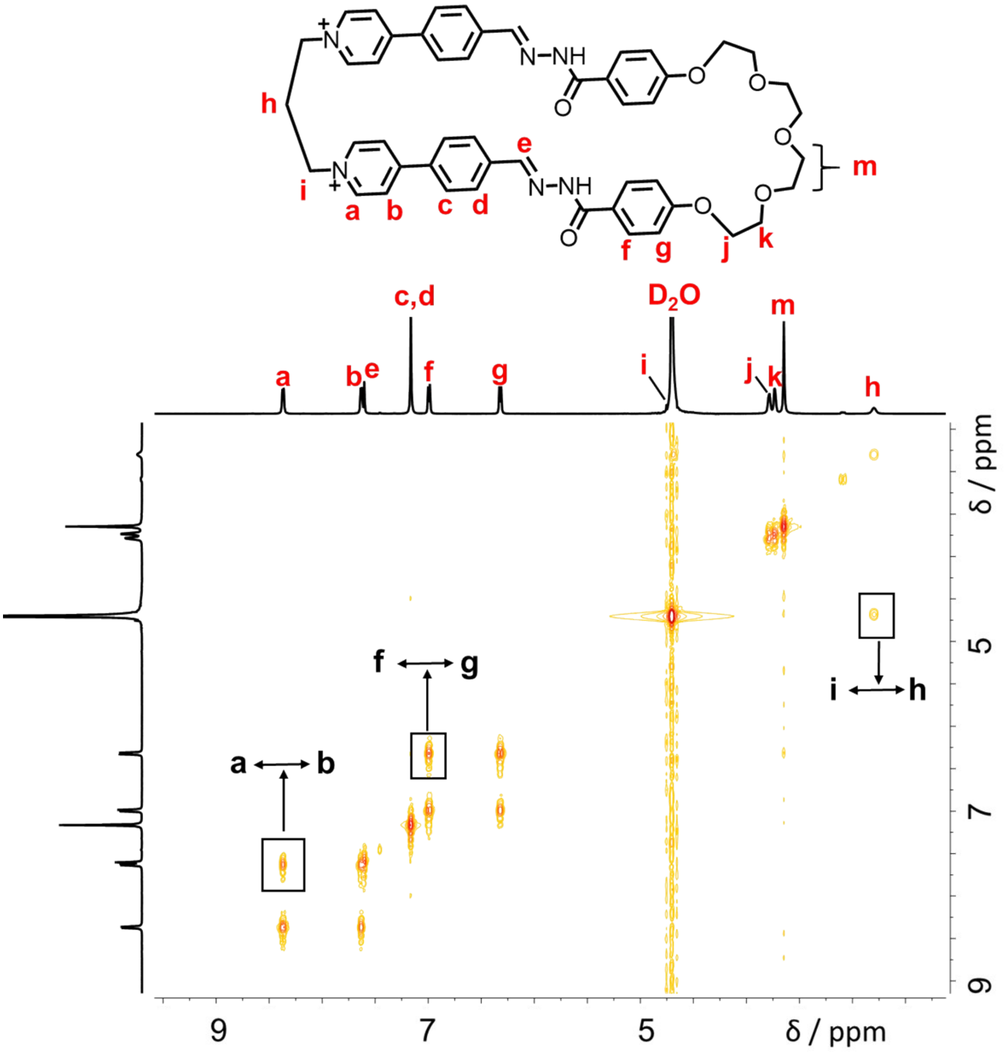


**Figure S4.** $^1H$–$^1H$ COSY spectrum (500 MHz, $D_2O$, 298 K) of (**1a**$^{2+}$·**2**). Counteranions could be either $Cl^-$ or $Br^-$. Key correlation peaks are labeled in the spectrum.

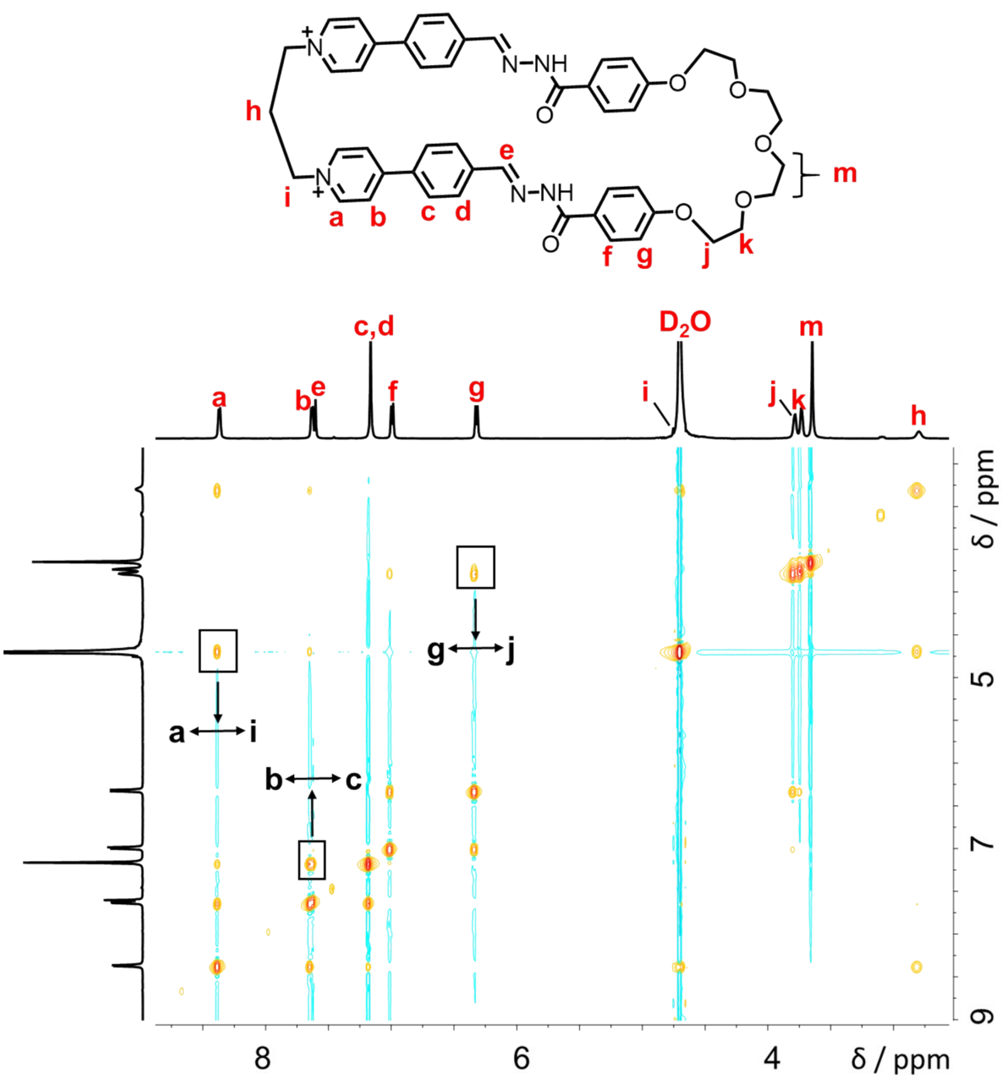


**Figure S5.** $^{1}H-^{1}H$ NOESY spectrum (500 MHz, $D_2O$, 298 K) of (**1a**$^{2+}$·**2**). Counteranions could be either $Cl^-$ or $Br^-$. Key correlation peaks are labeled in the spectrum.

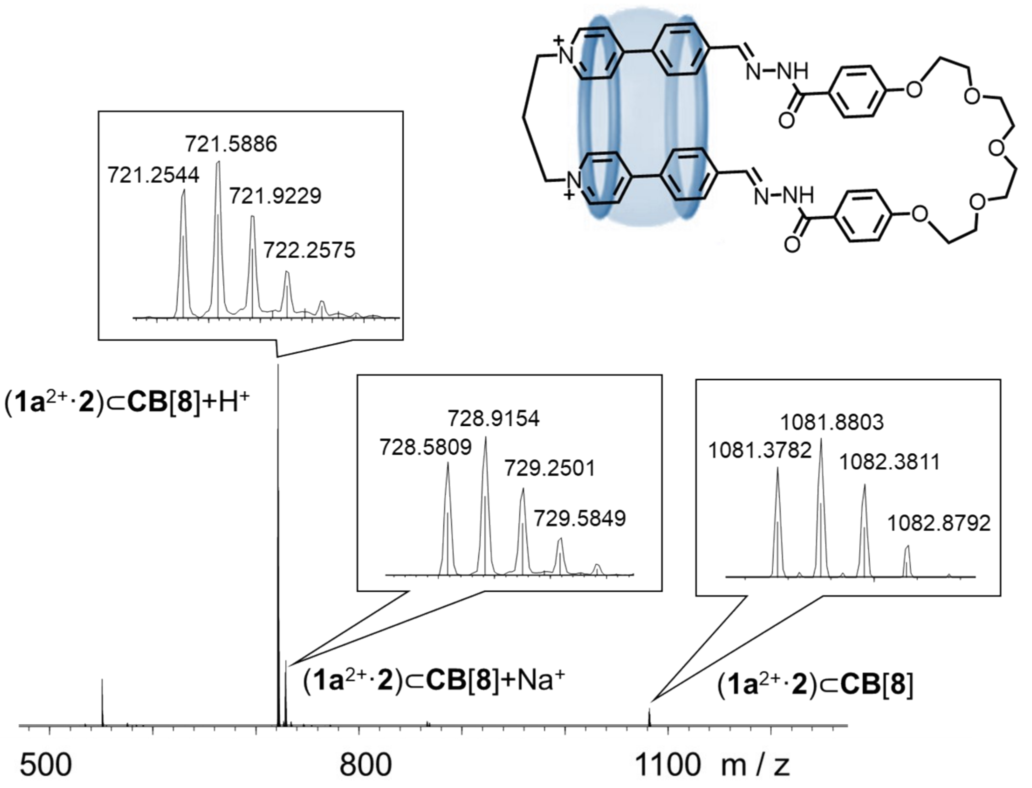


**Figure S6.** High-resolution LCMS-IT-TOF of (**1a**$^{2+}$·**2**)⊂**CB[8]**. Counteranions could be either Cl⁻ or Br⁻.The signals labeled in the spectrum correspond to molecular cations that contain three and two positive charges, respectively. *m/z* [(**1a**$^{2+}$·**2**)⊂**CB[8]** + Na$^{+}$]$^{3+}$ calculated for $C_{97}H_{98}N_{38}NaO_{23}^{3+}$: 728.9194; found: 728.9154. [(**1a**$^{2+}$·**2**)⊂**CB[8]** + H$^{+}$]$^{3+}$ calculated for $C_{97}H_{99}N_{38}O_{23}^{3+}$: 721.5921; found: 721.5886. [(**1a**$^{2+}$·**2**)⊂**CB[8]**]$^{2+}$ calculated for $C_{97}H_{98}N_{38}O_{23}^{2+}$: 1081.8845; found: 1081.8803.

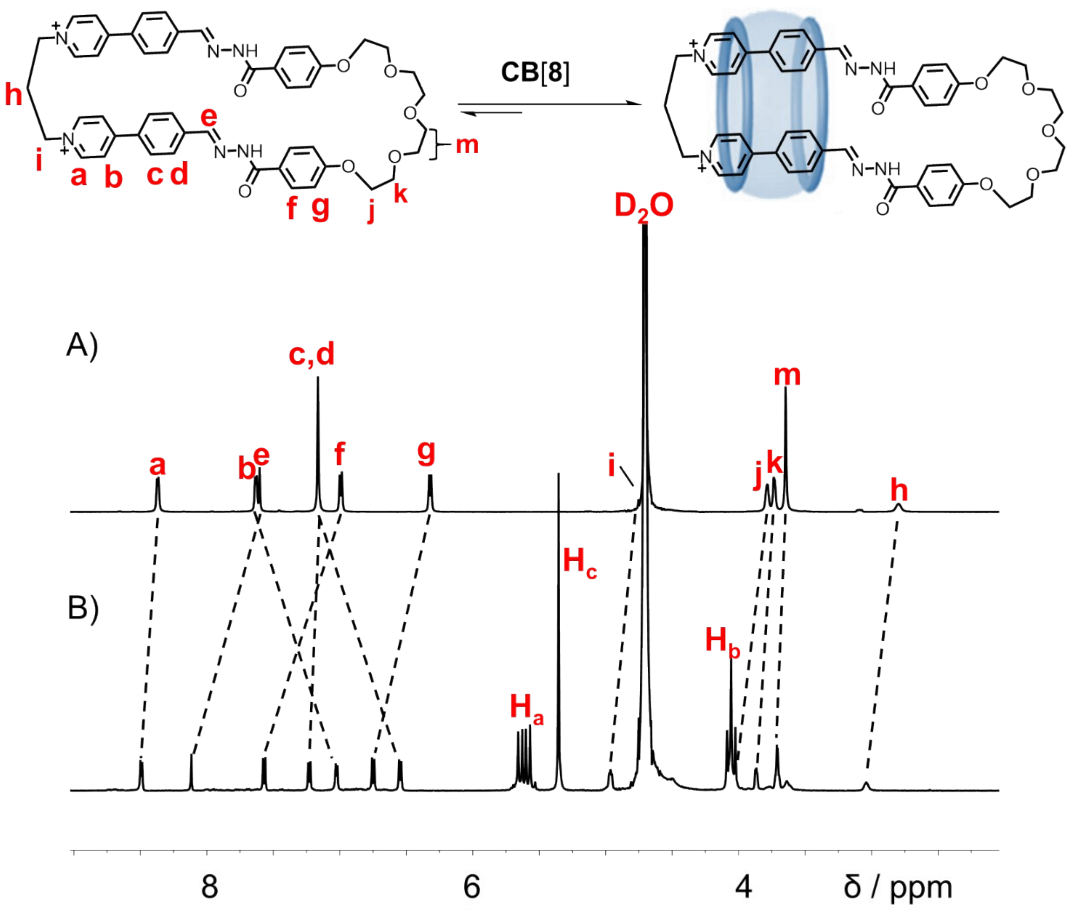


**Figure S7.** Partial $^{1}$H NMR spectrum (500 MHz, $D_2O$, 298 K) of A) (**1a**$^{2+}$·**2**) and B) (**1a**$^{2+}$·**2**)⊂**CB[8]**. Counteranions could be either $Cl^-$ or $Br^-$.

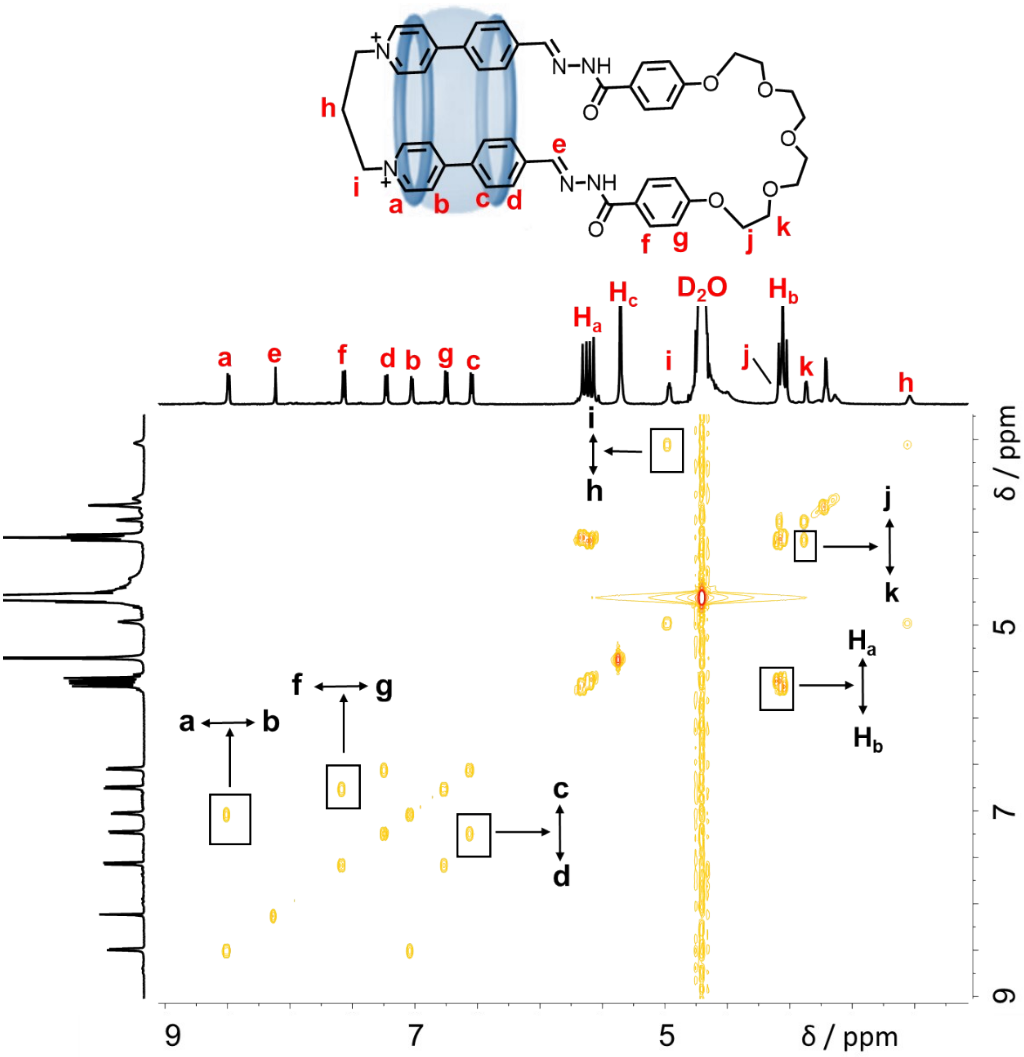


**Figure S8.** $^1H-^1H$ COSY spectrum (500 MHz, $D_2O$, 298 K) of (**1a**$^{2+}$·**2**)⊂**CB[8]**. Counteranions could be either $Cl^-$ or $Br^-$. Key correlation peaks are labeled in the spectrum.

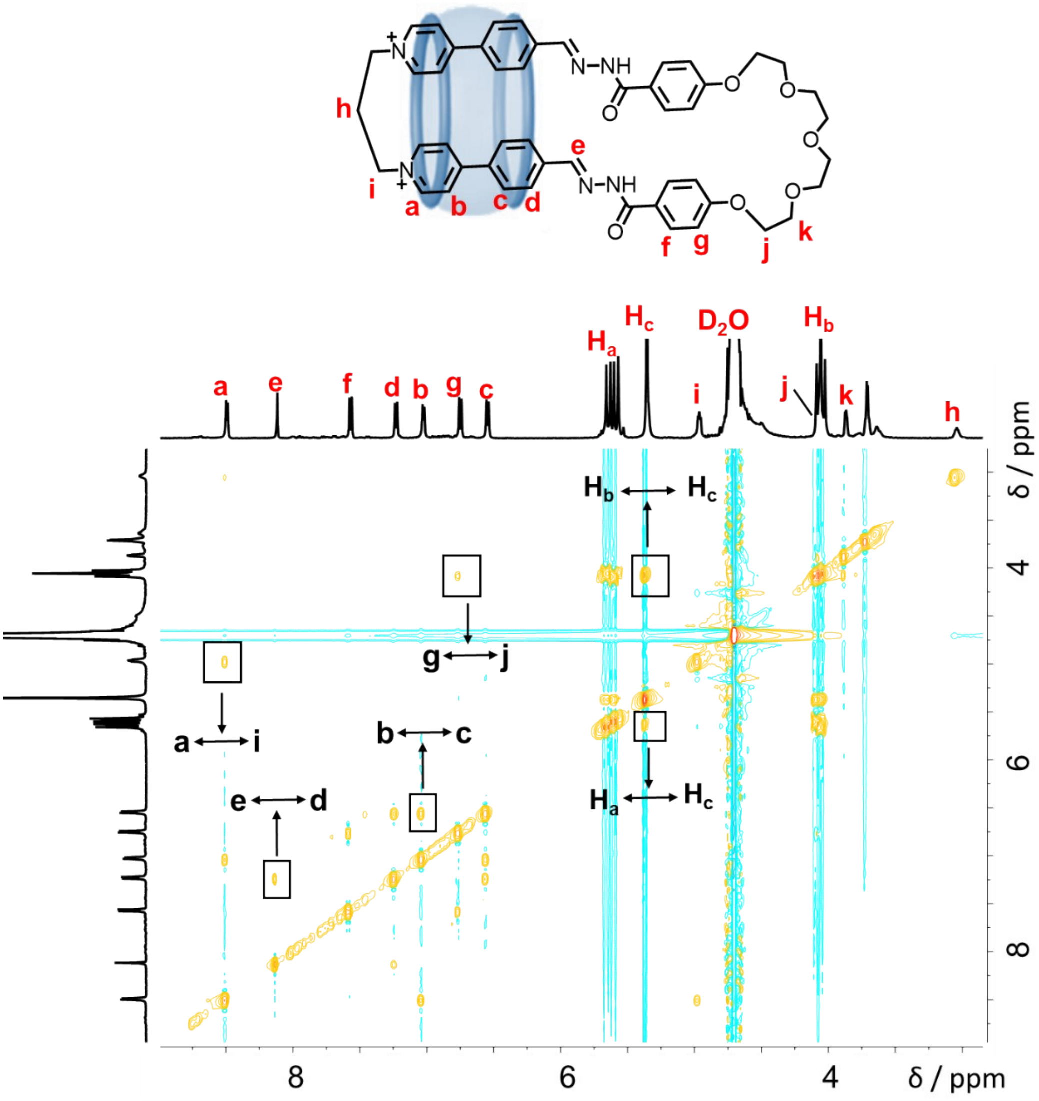


**Figure S9.** $^1$H−$^1$H NOESY spectrum (500 MHz, $D_2O$, 298 K) of (**1a**$^{2+}$·**2**)⊂**CB[8]**. Counteranions could be either $Cl^-$ or $Br^-$. Key correlation peaks are labeled in the spectrum.

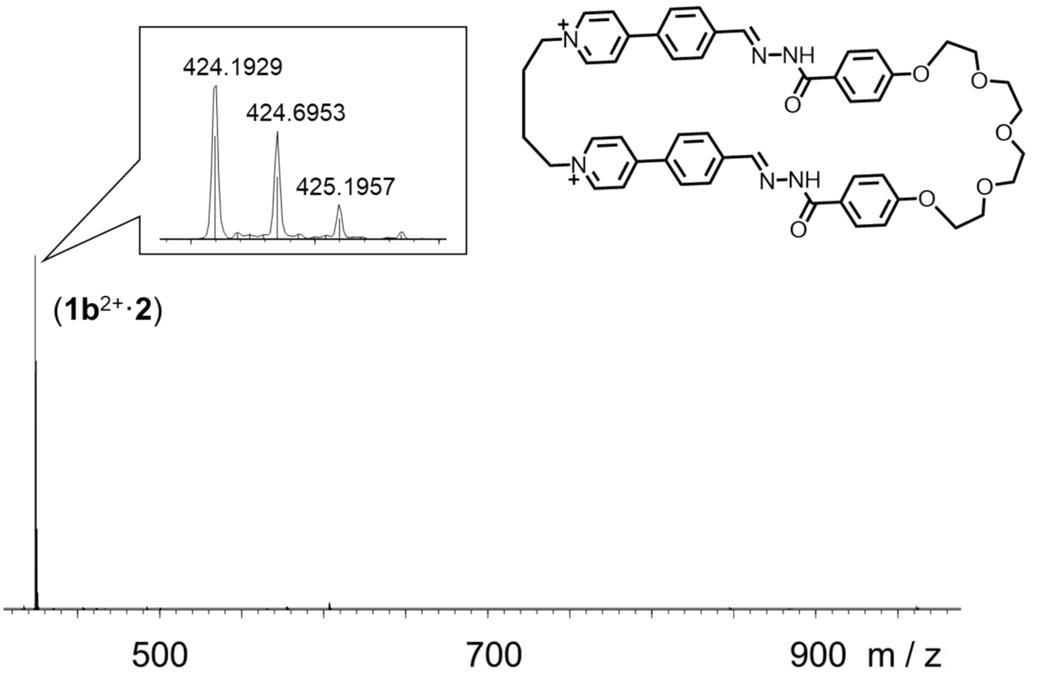


**Figure S10.** High-resolution LCMS-IT-TOF of (**1b**$^{2+}$·**2**). Counteranions could be either $Cl^{-}$ or $Br^{-}$. The signal labeled in the spectrum correspond to molecular cation that contains two positive charges. *m/z* [(**1b**$^{2+}$·**2**)]$^{2+}$ calculated for $C_{50}H_{52}N_6O_7^{2+}$: 424.1943; found: 424.1929.

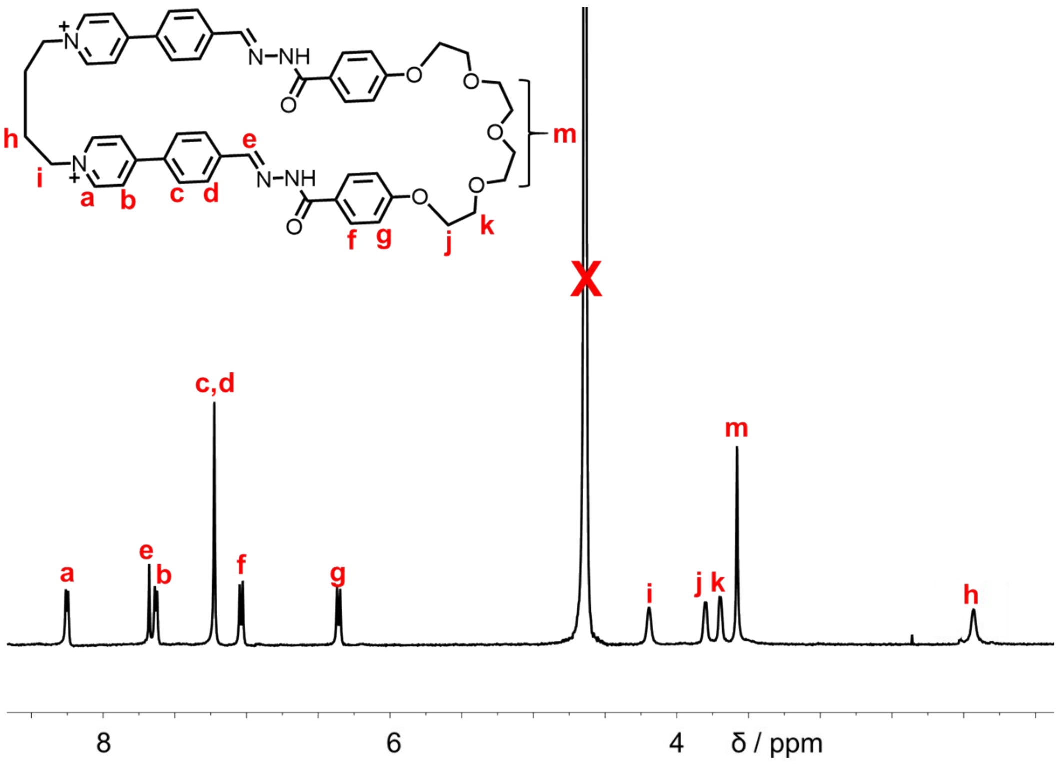


**Figure S11.** Partial $^{1}$H NMR spectrum of (**1b**$^{2+}$·**2**) (500 MHz, $D_2O$, 298 K). Counteranions could be either $Cl^{-}$ or $Br^{-}$.

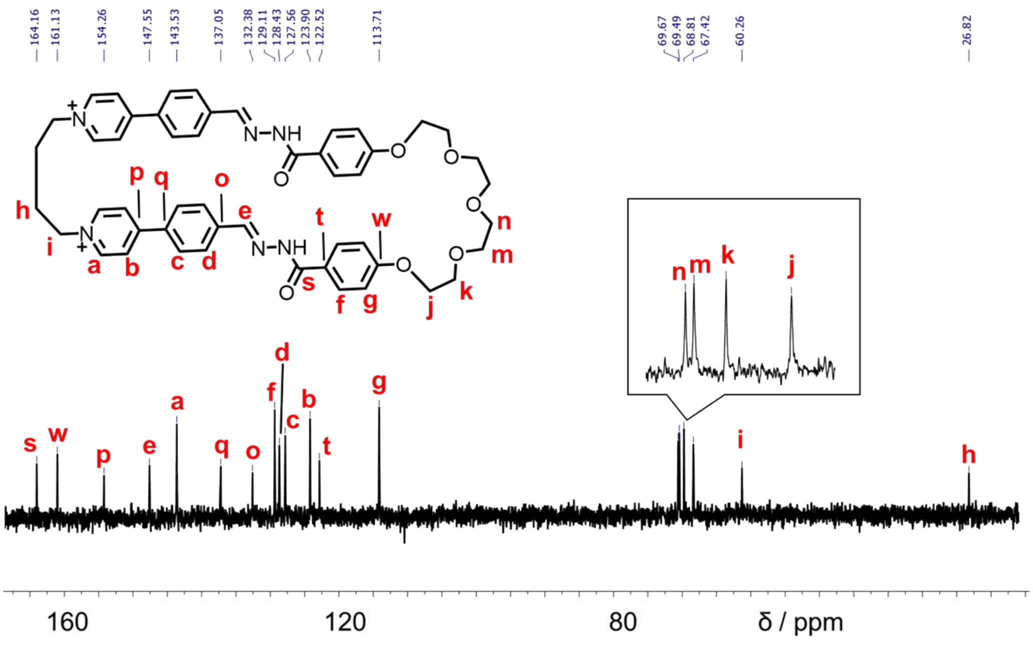

**Figure S12.** $^{13}C$ NMR spectrum (100 M Hz, 298 K, $D_2O$) of (**1b**$^{2+}$·**2**). Counteranions could be either $Cl^-$ or $Br^-$.

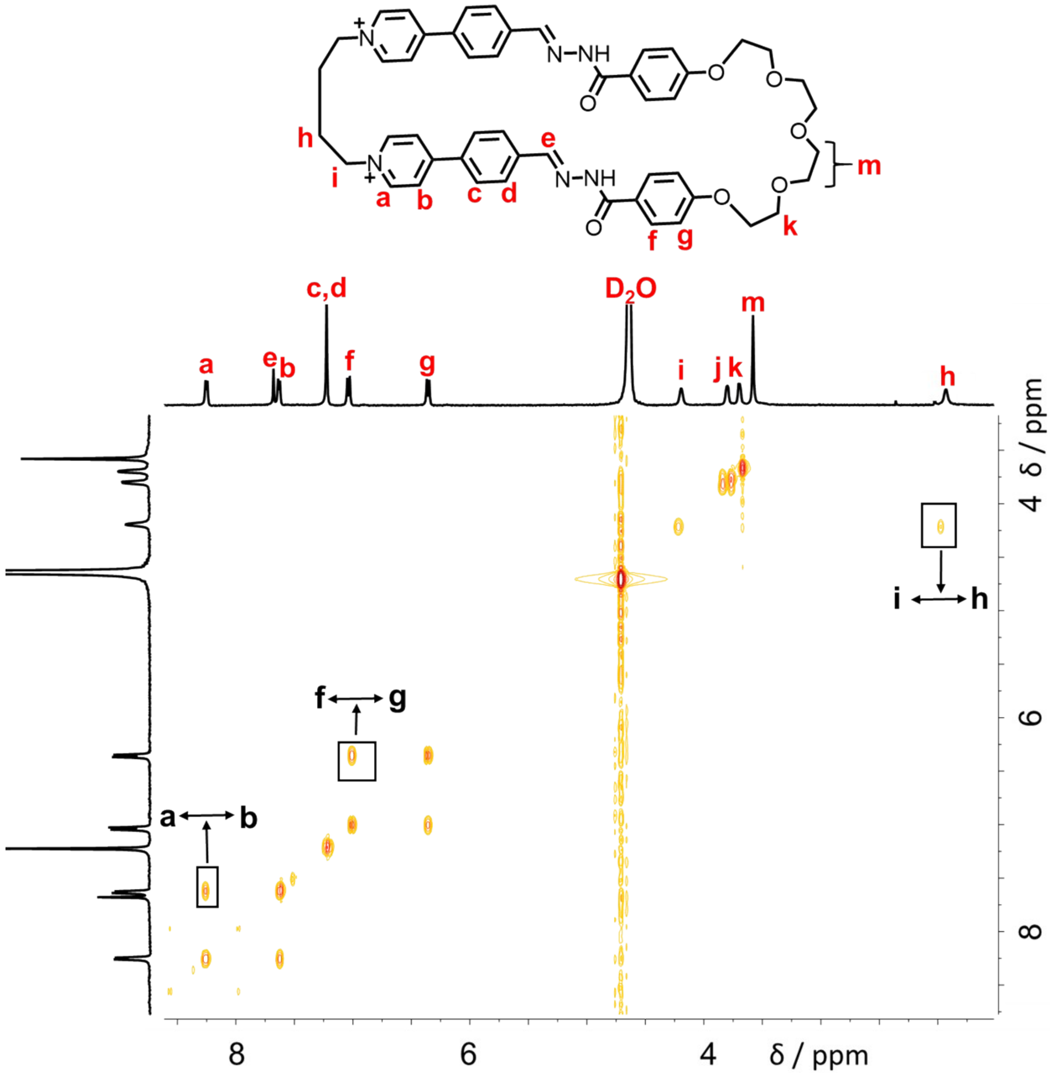


**Figure S13.** $^1H-^1H$ COSY spectrum (500 MHz, $D_2O$, 298 K) of (**1b**$^{2+}$·**2**). Counteranions could be either $Cl^-$ or $Br^-$. Key correlation peaks are labeled in the spectrum.

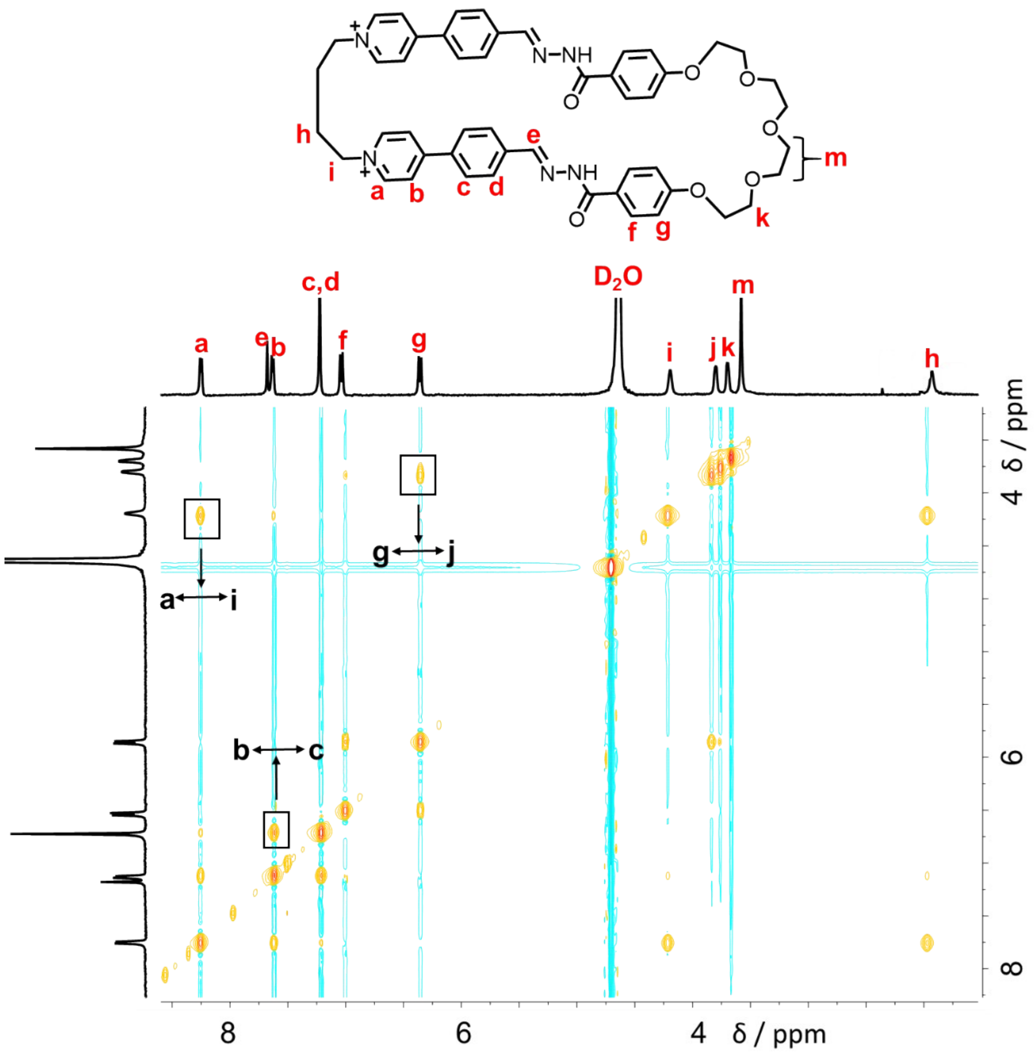


**Figure S14.** $^1H-^1H$ NOESY spectrum (500 MHz, $D_2O$, 298 K) of (**1b**$^{2+}$·**2**). Counteranions could be either $Cl^-$ or $Br^-$. Key correlation peaks are labeled in the spectrum.

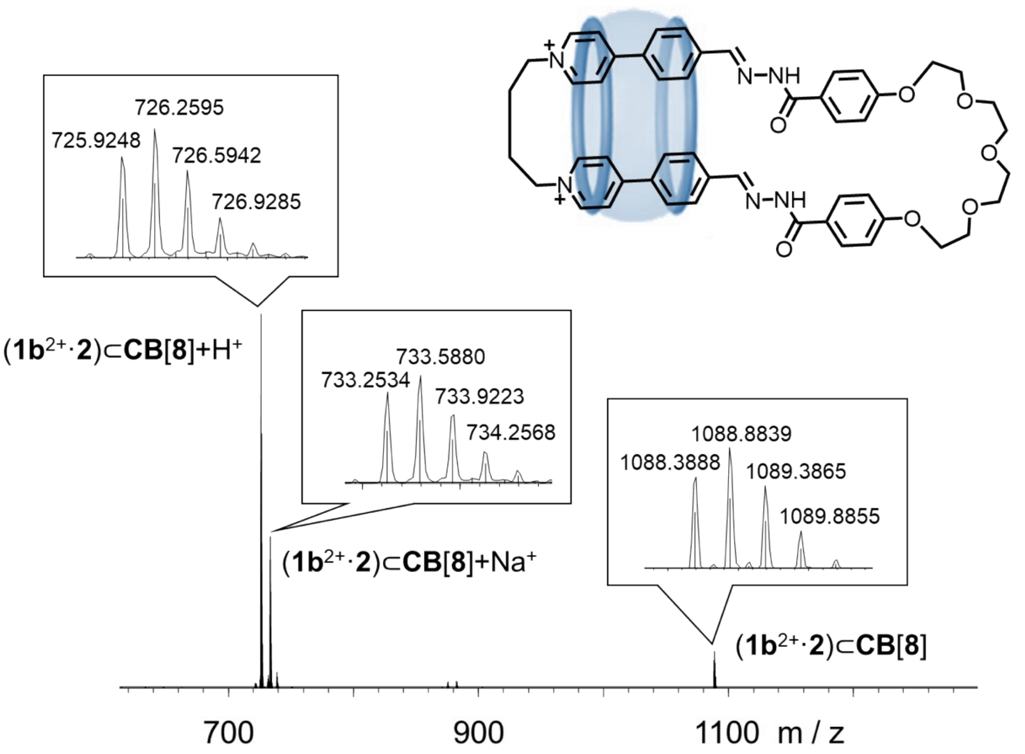


**Figure S15.** High-resolution LCMS-IT-TOF of (**1b**$^{2+}$·**2**)⊂**CB[8]**. Counteranions could be either $Cl^-$ or $Br^-$.The signals labeled in the spectrum correspond to molecular cations that contain three and two positive charges, respectively. *m/z* [(**1b**$^{2+}$·**2**)⊂**CB[8]** + $Na^+$]$^{3+}$ calculated for $C_{98}H_{100}N_{38}NaO_{23}{}^{3+}$: 733.5913; found: 733.5880. [(**1b**$^{2+}$·**2**)⊂**CB[8]** + $H^+$]$^{3+}$ calculated for $C_{98}H_{101}N_{38}O_{23}{}^{3+}$: 726.2640; found: 726.2595. [(**1b**$^{2+}$·**2**)⊂**CB[8]**]$^{2+}$ calculated for $C_{98}H_{100}N_{38}O_{23}{}^{2+}$: 1088.8923; found: 1088.8839.

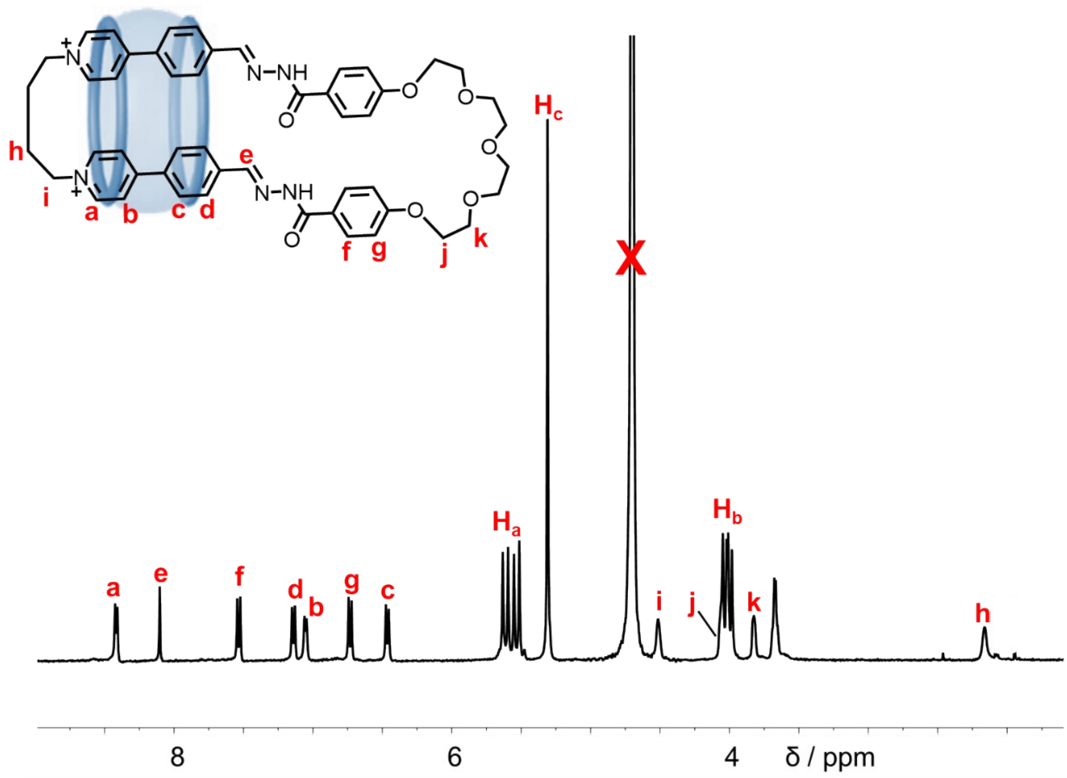


**Figure S16.** Partial $^1$H NMR spectrum of (**1b**$^{2+}$·**2**)⊂**CB[8]** (500 MHz, $D_2O$, 298 K). Counteranions could be either $Cl^-$ or $Br^-$.

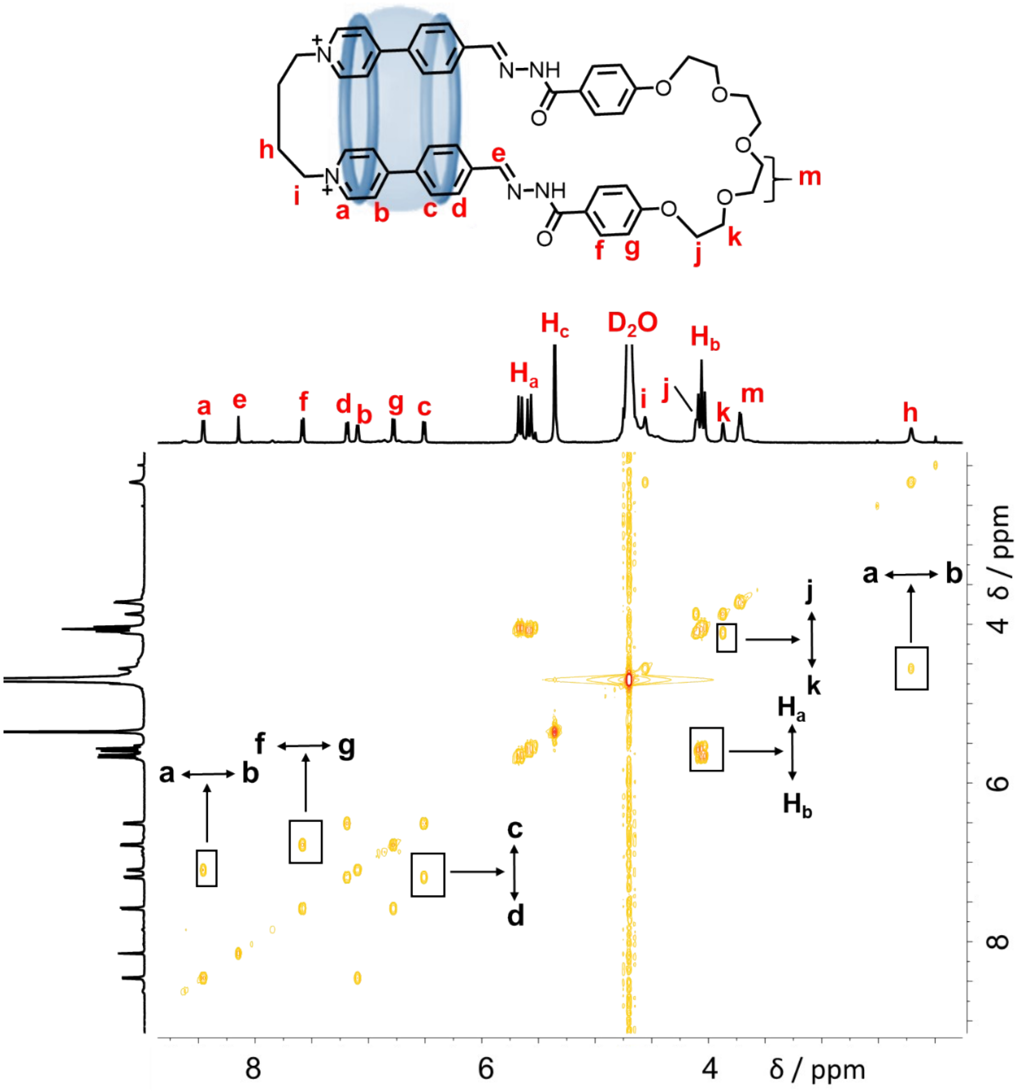


**Figure S17.** $^{1}H-^{1}H$ COSY spectrum (500 MHz, $D_2O$, 298 K) of (**1b**$^{2+}$·**2**)⊂**CB[8]**. Counteranions could be either $Cl^-$ or $Br^-$. Key correlation peaks are labeled in the spectrum.

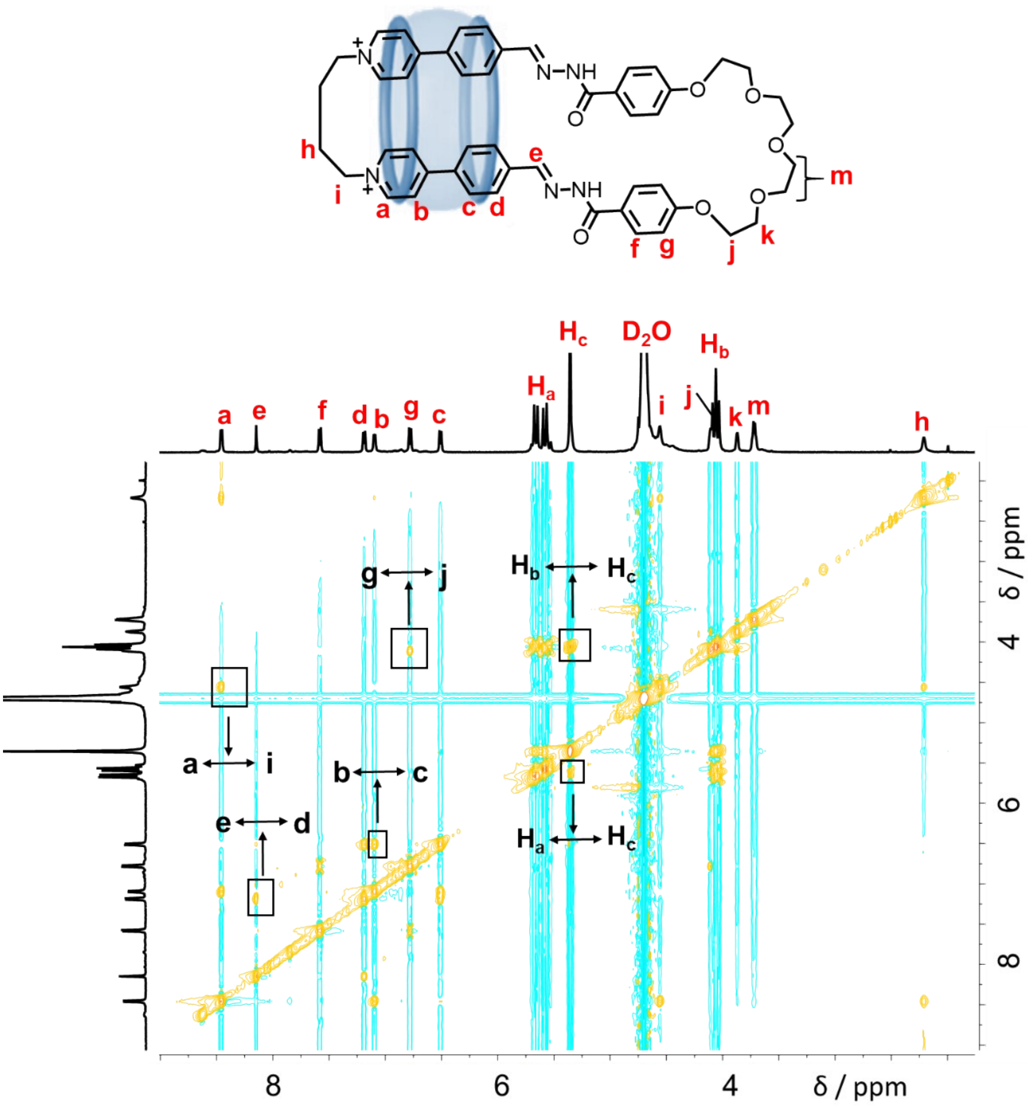


**Figure S18.** $^1$H−$^1$H NOESY spectrum (500 MHz, $D_2O$, 298 K) of (**1b**$^{2+}$·**2**)⊂**CB[8]**. Counteranions could be either $Cl^-$ or $Br^-$. Key correlation peaks are labeled in the spectrum.

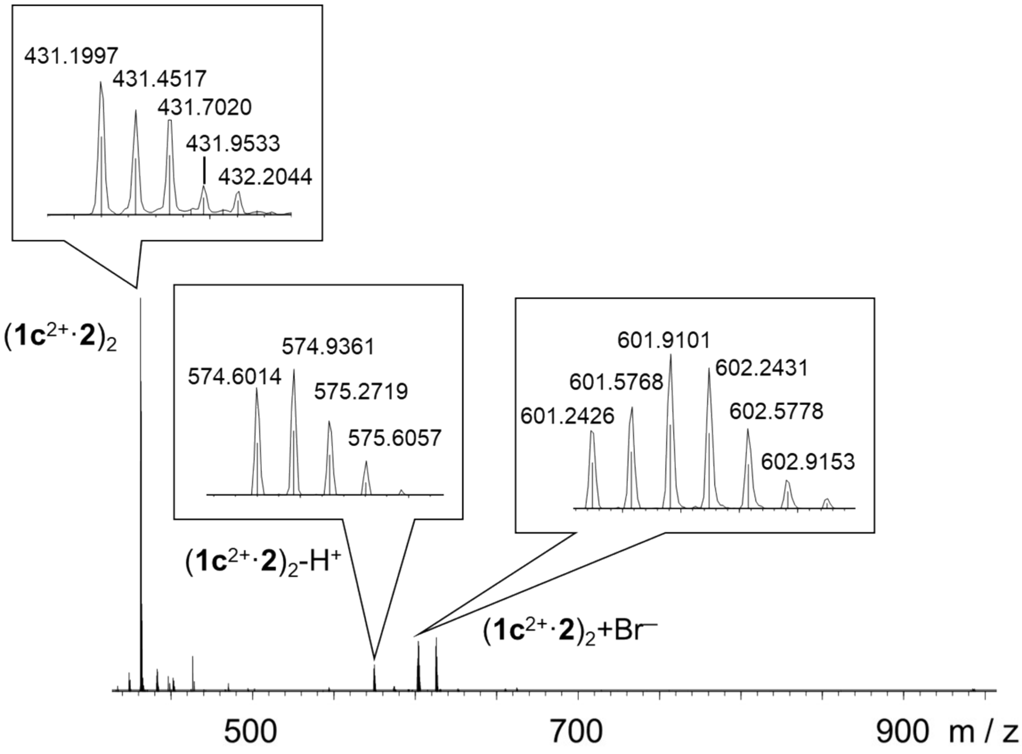


**Figure S19.** High-resolution LCMS-IT-TOF of the reaction mixture by condensing a 1:1 mixture of **1c**$^{2+}$2Br$^{-}$ (4.5 mM) and **2** (4.5 mM). Counteranions could be either Cl$^{-}$ or Br$^{-}$.The signals labeled in the spectrum correspond to molecular cations that contain four and three positive charges, respectively. *m/z* [(**1c**$^{2+}$·**2**)$_2$]$^{4+}$ calculated for $C_{102}H_{108}N_{12}O_{14}{}^{4+}$: 431.4530; found: 431.4517. [(**1c**$^{2+}$·**2**)$_2$ − H$^{+}$]$^{3+}$ calculated for $C_{102}H_{107}N_{12}O_{14}{}^{3+}$: 574.9349; found: 574.9361. [(**1c**$^{2+}$·**2**)$_2$ + Br$^{-}$]$^{3+}$ calculated for $C_{102}H_{108}BrN_{12}O_{14}{}^{4+}$: 602.2429; found: 602.2431.

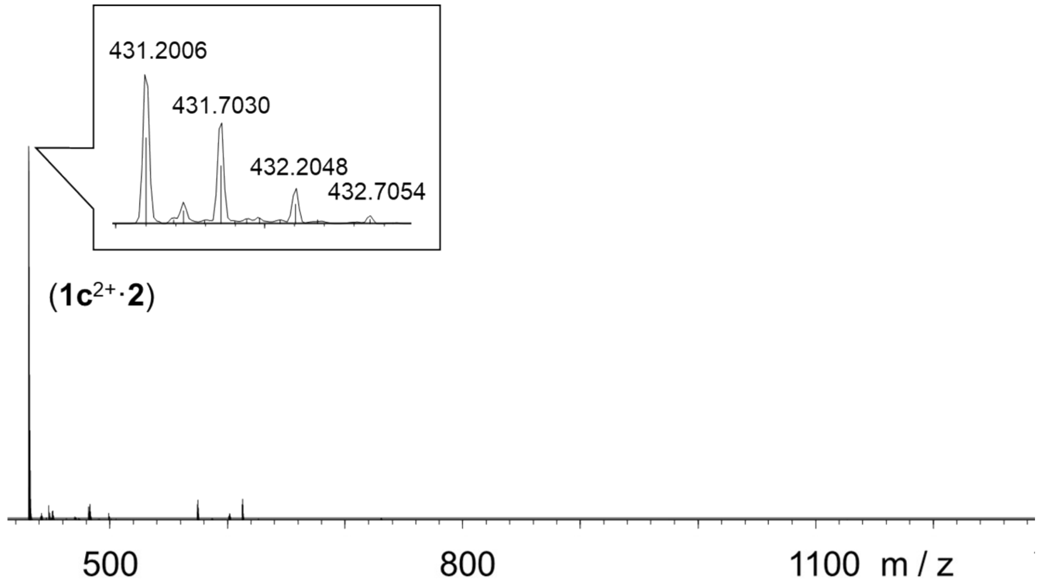


**Figure S20.** High-resolution LCMS-IT-TOF of the reaction mixture by condensing a 1:1 mixture of **1c**$^{2+}$2Br$^{-}$ (0.6 mM) and **2** (0.6 mM). Counteranions could be either Cl$^{-}$ or Br$^{-}$. The signal labeled in the spectrum correspond to molecular cation that contain two positive charges. *m/z* [(**1c**$^{2+}$·**2**)]$^{2+}$ calculated for $C_{51}H_{54}N_6O_7{}^{2+}$: 431.2022; found: 431.2006.

Figures S19 and S20 indicated that an equilibrium was established between the [2]catenane (**1c**$^{2+}$·**2**)$_2$ and the ring (**1c**$^{2+}$·**2**). At higher concentration (Figure S19), the [2]catenane (**1c**$^{2+}$·**2**)$_2$ was observed as the major product; while at lower concentration, equilibrium shifted to the side of the ring (**1c**$^{2+}$·**2**).

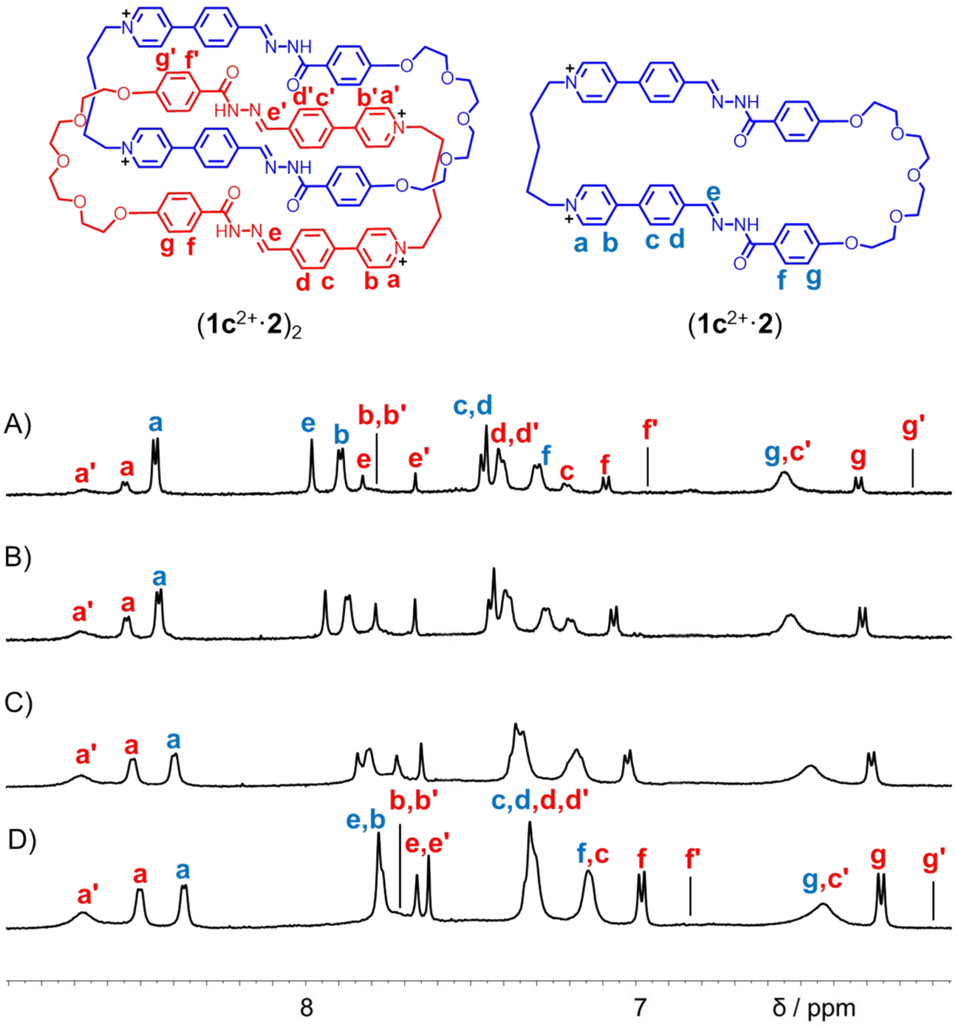


**Figure S21.** Partial $^{1}$H NMR spectrum of the reaction mixture by condensing a 1:1 mixture of **1c**$^{2+}$2Br$^{-}$ and **2** at different concentrations (500 MHz, $D_2O$, 298 K): A) 0.6 mM, B) 1.2 mM, C) 2.5 mM, D) 4.5 mM . Counteranions could be either $Cl^{-}$ or $Br^{-}$. All the spectra were recorded after the systems reached their equilibria.

The $^{1}$H NMR spectra indicated that the products of condensing **1c**$^{2+}$2Br$^{-}$ and **2** were concentration dependent in $D_2O$. Generally, low reaction concentrations were observed to favor the ring production (**1c**$^{2+}$·**2**) over its corresponding catenane counterpart (**1c**$^{2+}$·**2**)$_2$ (Figure S21 A and S20). While at higher concentration, the [2]catenane (**1c**$^{2+}$·**2**)$_2$ became more favored (Figure S21 and S19).

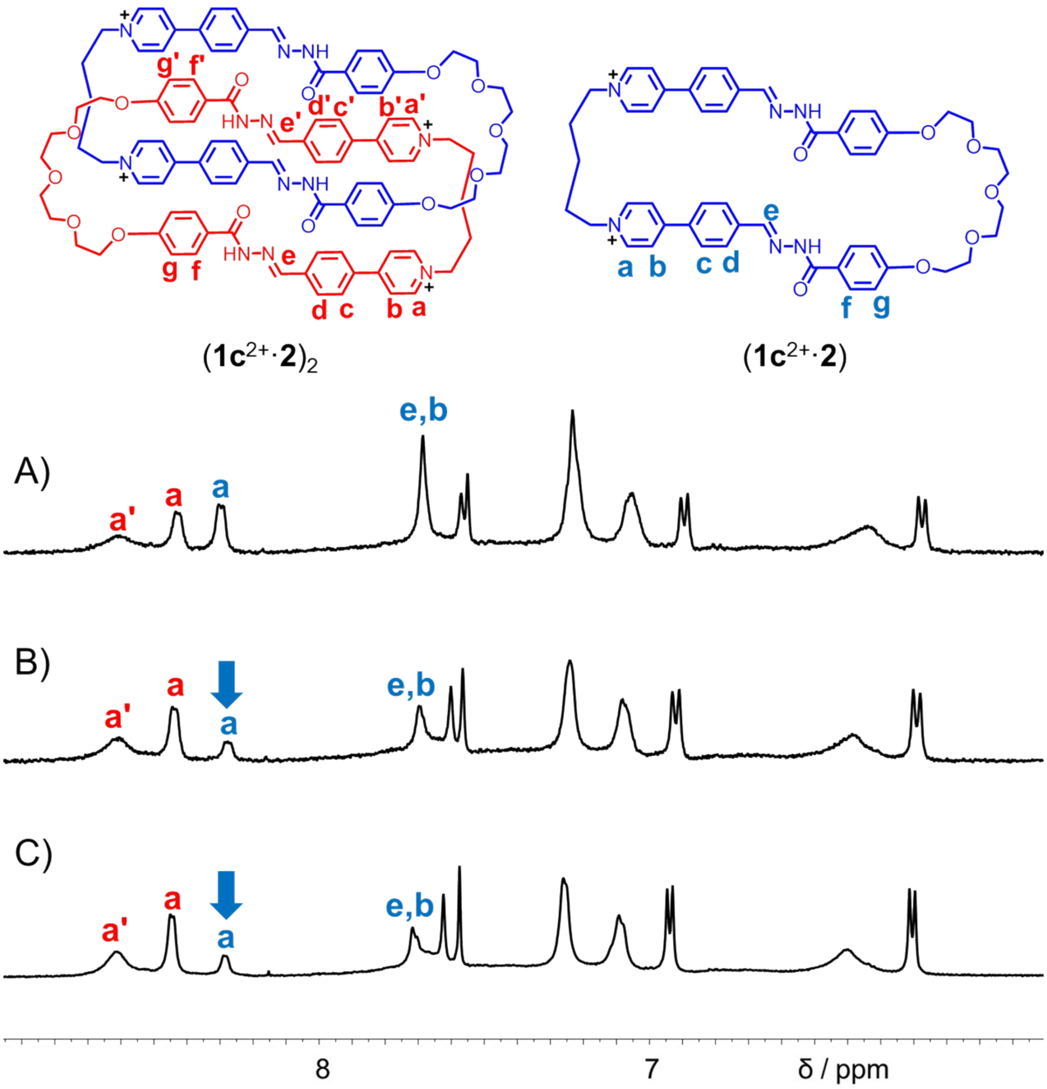


**Figure S22.** Partial $^{1}H$ NMR spectrum of the reaction mixture by condensing a 1:1 mixture of $\mathbf{1c}^{2+}2Br^{-}$ and **2** (4.5 mM) (500 MHz, $D_2O$, 298 K), which were recorded after the reactants were mixed for A) 12 h, B) 4 d and C) 6 d. Counterions could be either $Cl^{-}$ or $Br^{-}$.

$^{1}H$ NMR spectra indicate that in the early stage of reaction (Figure S22 A), the resonances corresponding to the macrocycle ($\mathbf{1c}^{2+}\cdot\mathbf{2}$) are much stronger than those after the system reached the equilibrium (Figure S22 C). Such observation indicated that the ring is a kinetic product, while the [2]catenane is the thermodynamic favored product at higher concentration namely 4.5 mM.

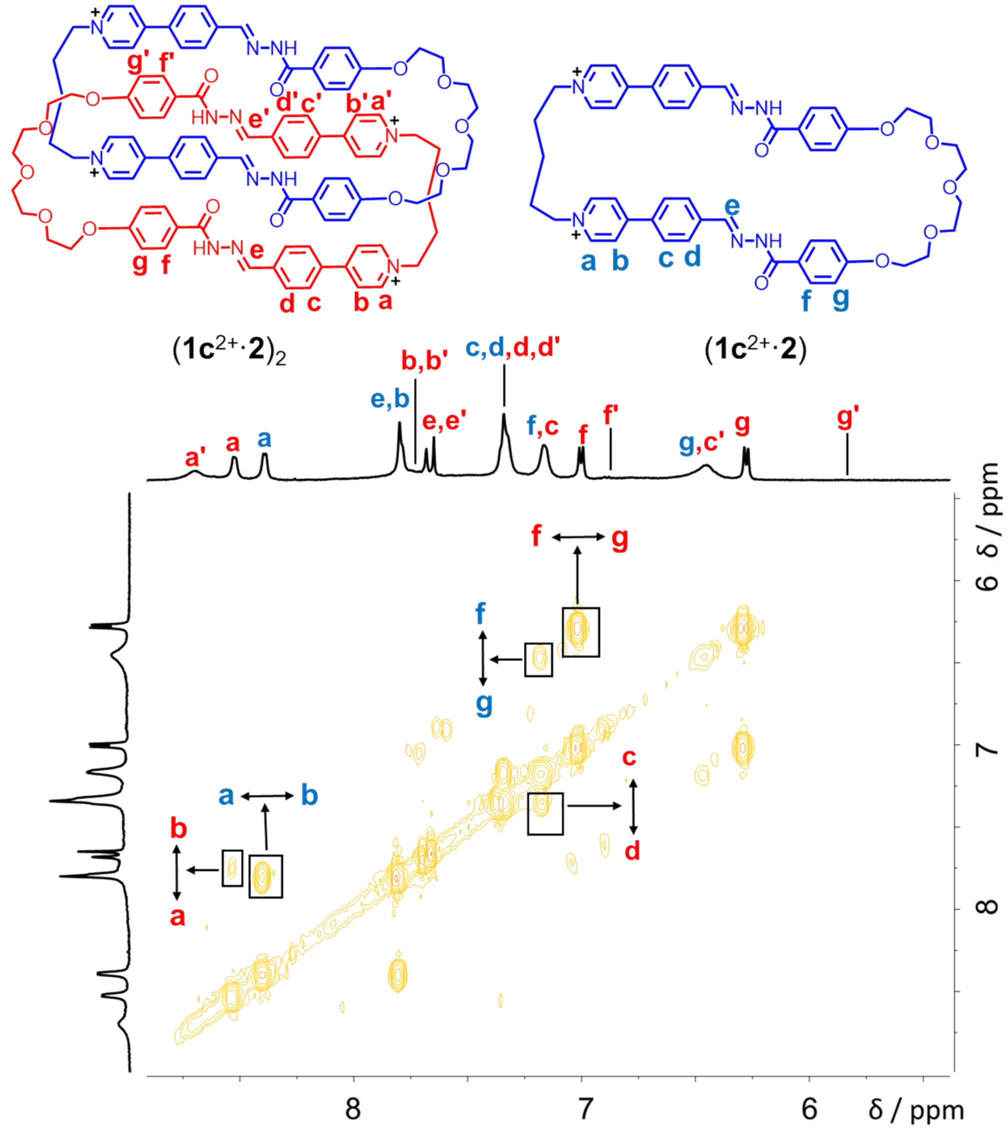


**Figure S23.** $^1H-^1H$ COSY spectrum (500 MHz, $D_2O$, 298 K) of the reaction mixture by condensing a 1:1 mixture of **1c**$^{2+}$2Br$^-$ (4.5 mM) and **2** (4.5 mM). Counteranions could be either Cl$^-$ or Br$^-$. Key correlation peaks are labeled in the spectrum.

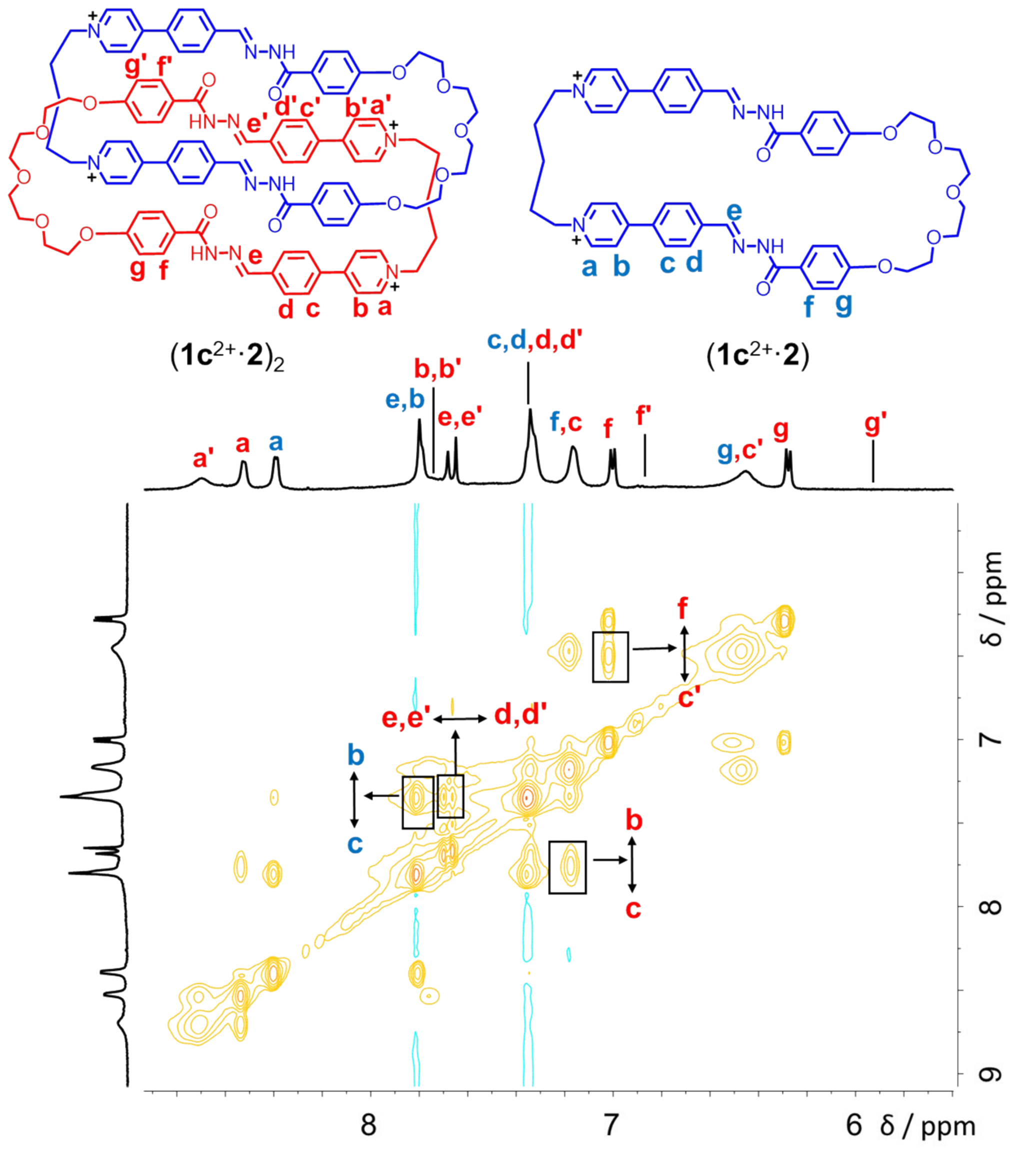


**Figure S24.** $^{1}H-^{1}H$ NOESY spectrum (500 MHz, $D_2O$, 298 K) of the reaction mixture by condensing a 1:1 mixture of **1c**$^{2+}$2Br$^{-}$ (4.5 mM) and **2** (4.5 mM). Counteranions could be either $Cl^{-}$ or $Br^{-}$. Key correlation peaks are labeled in the spectrum.

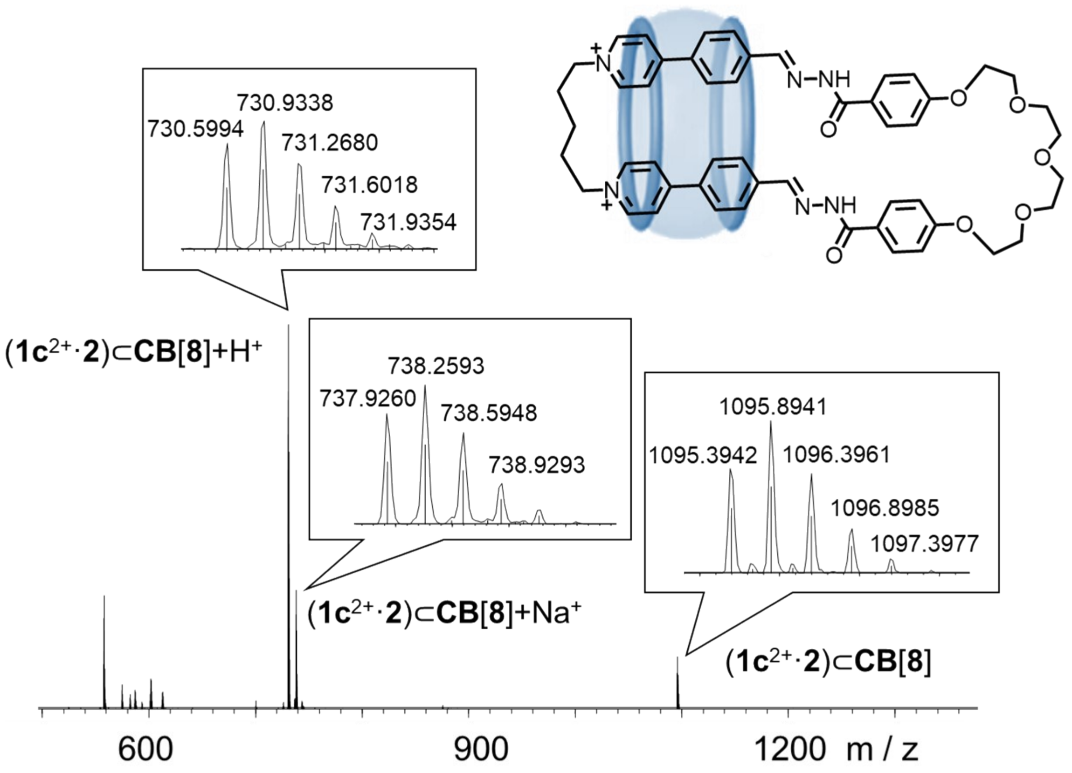


**Figure S25.** High-resolution LCMS-IT-TOF of (**1c**$^{2+}$·**2**)⊂**CB[8]**. Counteranions could be either $Cl^-$ or $Br^-$.The signals labeled in the spectrum correspond to molecular cations that contain three and two positive charges, respectively. *m/z* [(**1c**$^{2+}$·**2**)⊂**CB[8]** + $Na^+$]$^{3+}$ calculated for $C_{99}H_{102}N_{38}NaO_{23}^{3+}$: 738.2632; found: 738.2593. [(**1c**$^{2+}$·**2**)⊂**CB[8]** + $H^+$]$^{3+}$ calculated for $C_{99}H_{103}N_{38}O_{23}^{3+}$: 730.9358; found: 930.9338. [(**1c**$^{2+}$·**2**)⊂**CB[8]**]$^{2+}$ calculated for $C_{99}H_{102}N_{38}O_{23}^{2+}$: 1095.9001; found: 1095.8941.

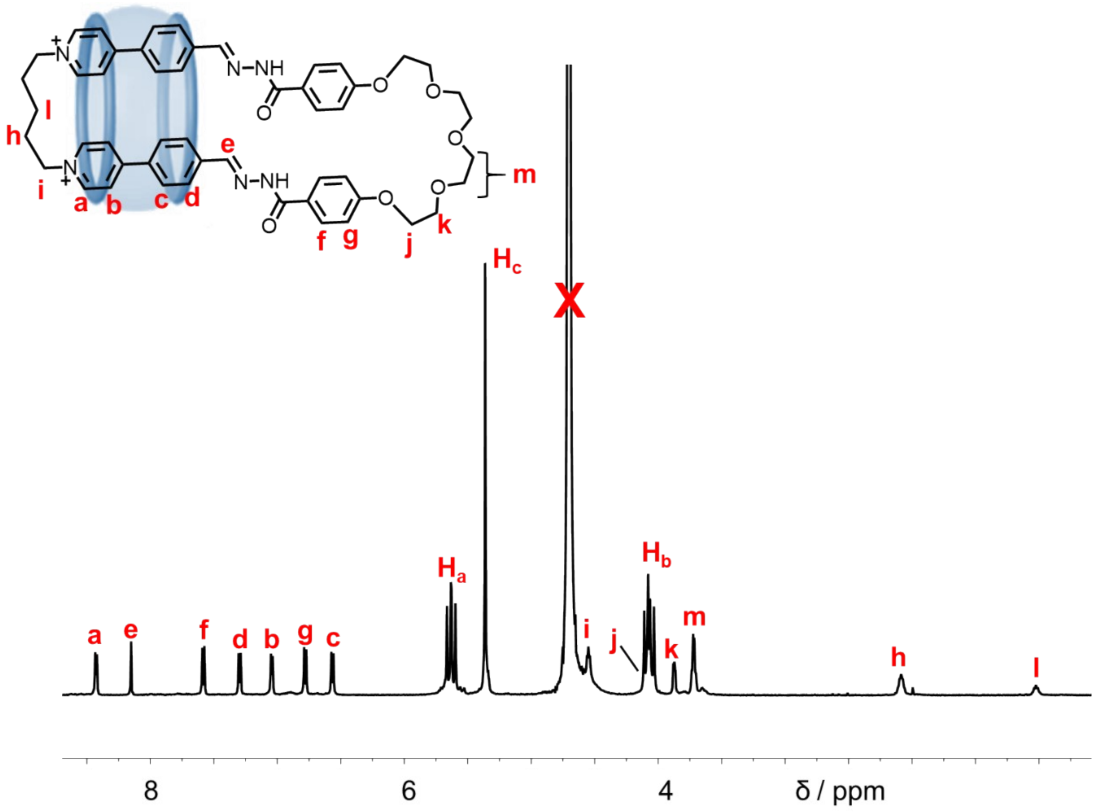


**Figure S26.** Partial $^1H$ NMR spectrum of (**1c**$^{2+}$·**2**)⊂**CB[8]** (500 MHz, $D_2O$, 298 K). Counteranions could be either $Cl^-$ or $Br^-$.

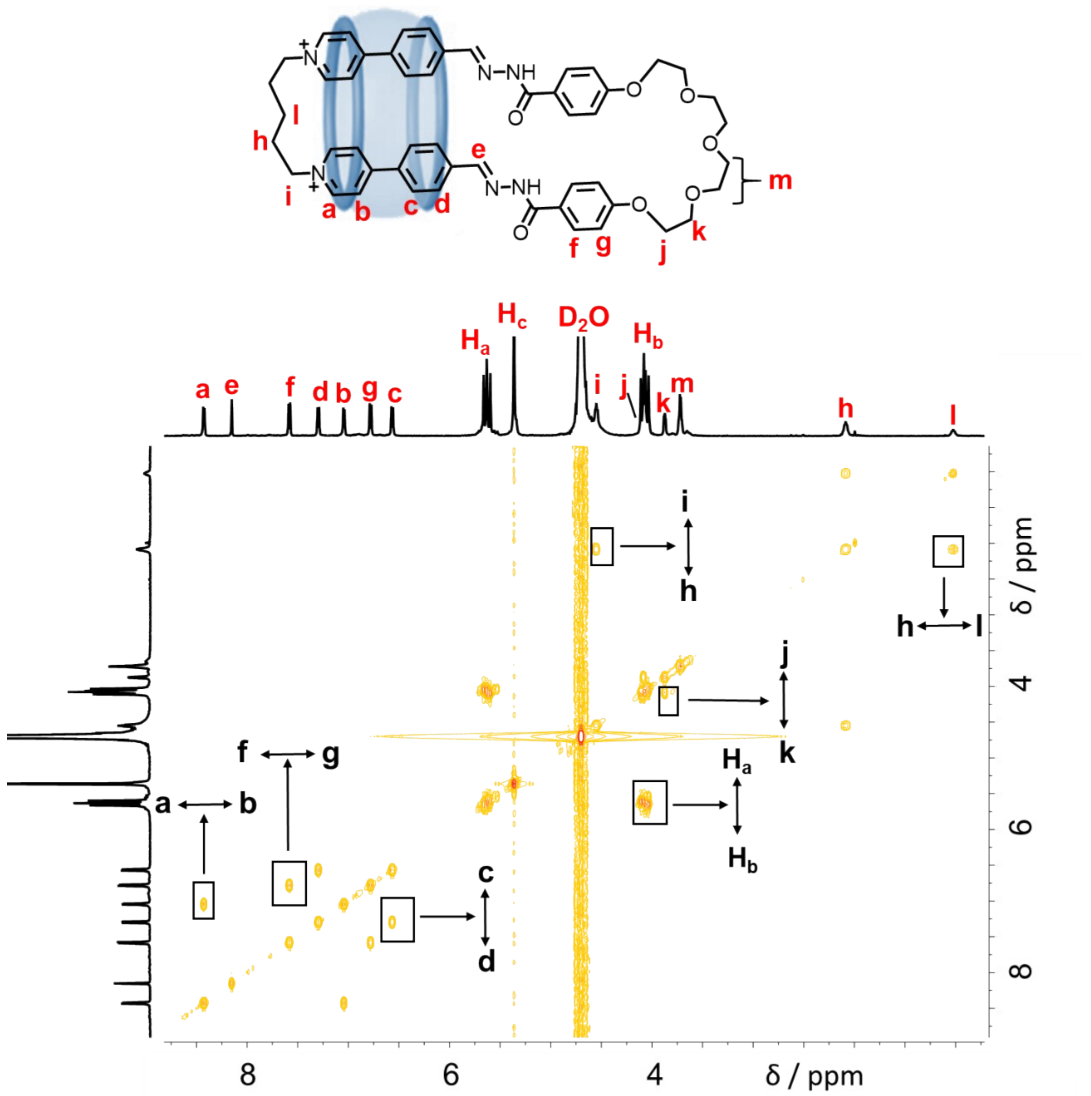


**Figure S27.** $^1$H–$^1$H COSY spectrum (500 MHz, $D_2O$, 298 K) of (**1c**$^{2+}$·**2**)⊂**CB[8]**. Counteranions could be either $Cl^-$ or $Br^-$. Key correlation peaks are labeled in the spectrum.

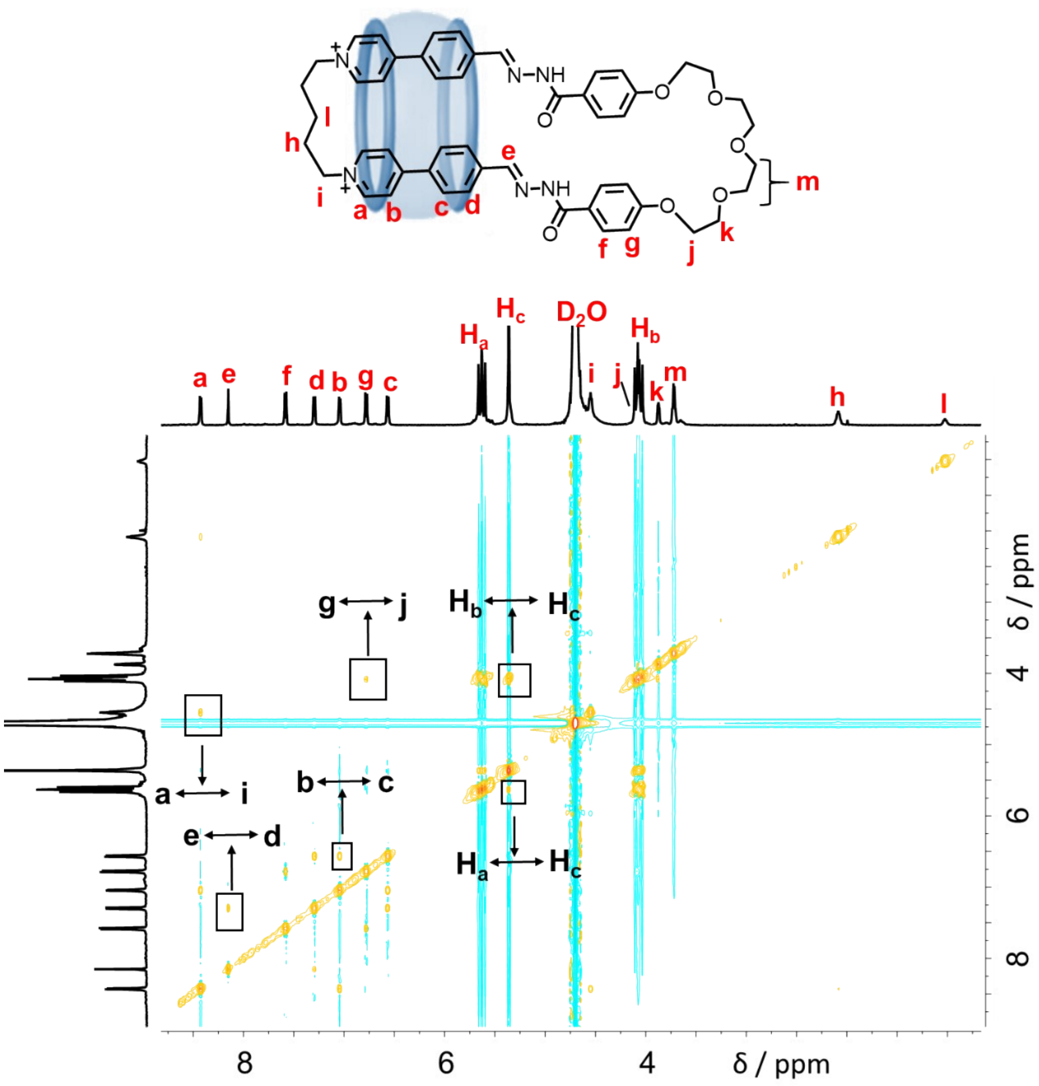


**Figure S28.** $^1$H−$^1$H NOESY spectrum (500 MHz, $D_2O$, 298 K) of (**1c**$^{2+}$·**2**)⊂**CB**[**8**]. Counteranions could be either $Cl^-$ or $Br^-$. Key correlation peaks are labeled in the spectrum.

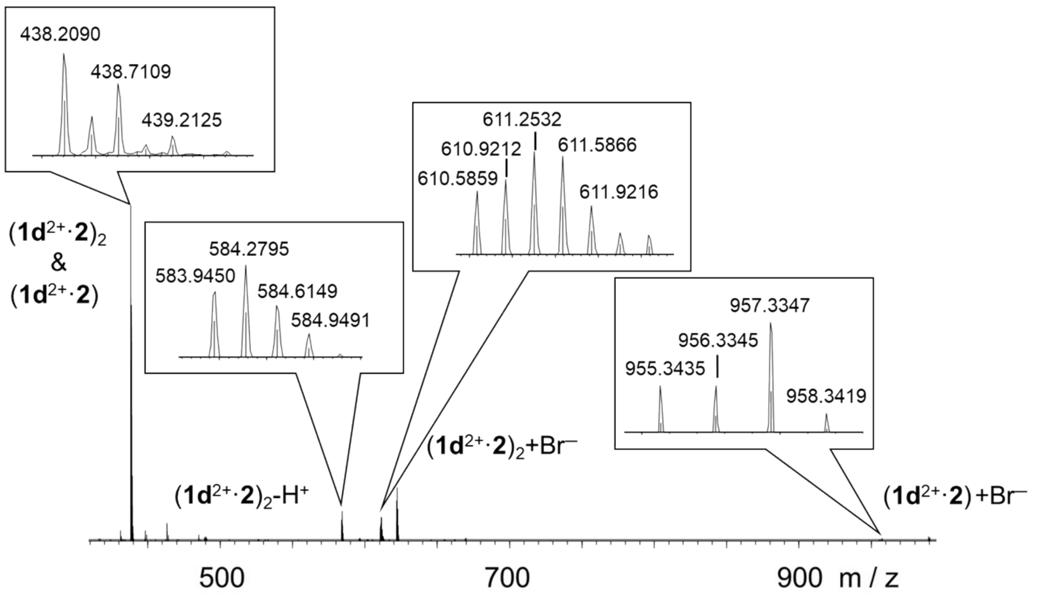


**Figure S29.** High-resolution LCMS-IT-TOF of the reaction mixture by condensing a 1:1 mixture of **1d**$^{2+}$2Br$^{-}$ (2.5 mM) and **2** (2.5 mM) in water. Counteranions could be either Cl$^{-}$ or Br$^{-}$.The signals labeled in the spectrum correspond to molecular cations that contain four, three, two and one positive charges, respectively. *m/z* [(**1d**$^{2+}$·**2**)$_2$ − H$^{+}$]$^{3+}$ calculated for $C_{104}H_{111}N_{12}O_{14}{}^{3+}$: 584.2787; found: 584.2795. [(**1d**$^{2+}$·**2**)$_2$ + Br$^{-}$]$^{3+}$ calculated for $C_{104}H_{112}BrN_{12}O_{14}{}^{4+}$: 611.5867; found: 611.5866. [(**1d**$^{2+}$·**2**) + Br$^{-}$]$^{+}$ calculated for $C_{104}H_{112}BrN_{12}O_{14}{}^{4+}$: 955.3388; found: 955.3435.

In mass spectrum, peaks corresponding to both the [2]catenane (**1d**$^{2+}$·**2**)$_2$ and the macrocycle (**1d**$^{2+}$·**2**) (Figure S29) were observed.

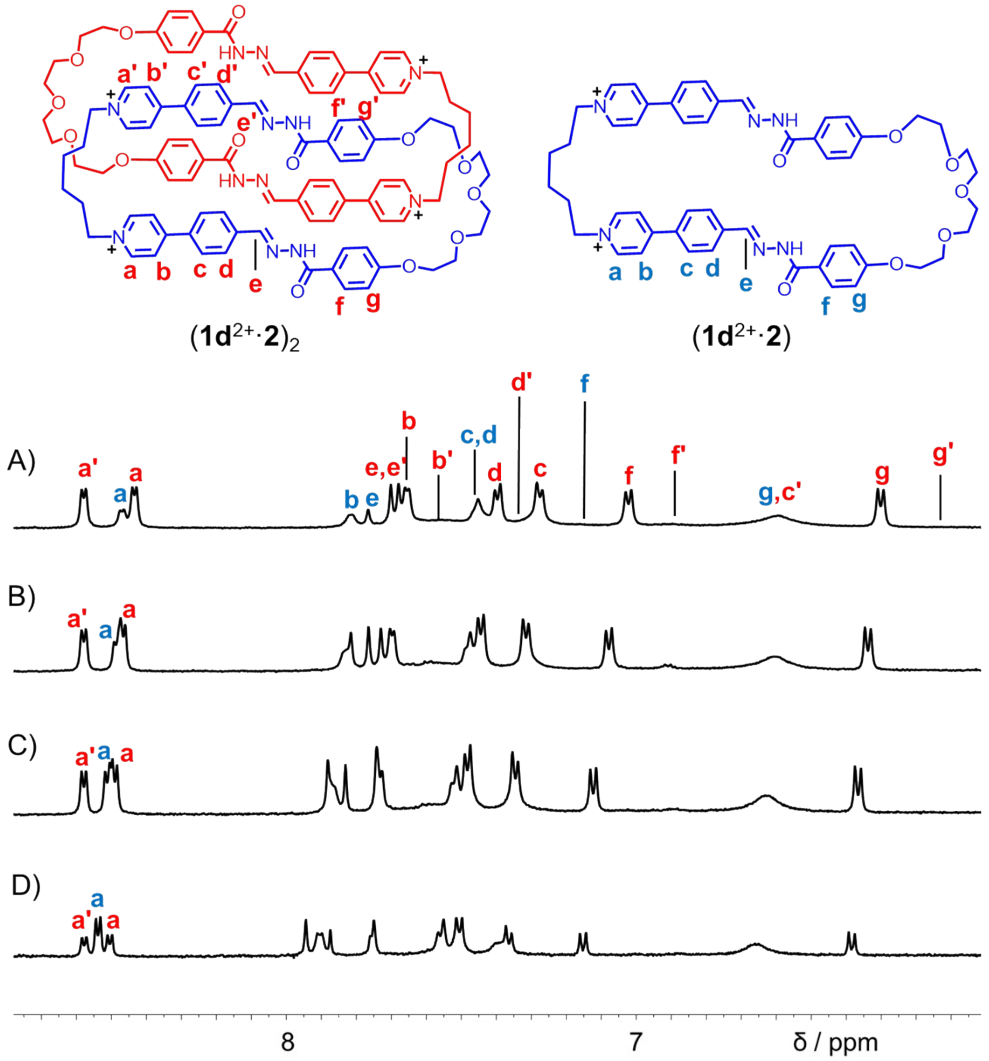


**Figure S30.** Partial $^1$H NMR spectrum of the reaction mixture by condensing a 1:1 mixture of **1d**$^{2+}$2Br⁻ and **2** at different concentrations (500 MHz, $D_2O$, 298 K), including A) 4.5 mM, B) 2.5 mM, C) 1.2 mM, D) 0.6 mM . Counteranions could be either Cl⁻ or Br⁻.

The $^1$H NMR spectra indicated that the reaction products of condensing **1d**$^{2+}$2Br⁻ and **2** in water is concentration dependent. Generally, at lower reaction concentration (Figure S30 D), the ring (**1d**$^{2+}$·**2**) is more favored compared that observed at higher concentration (Figure S30 A).

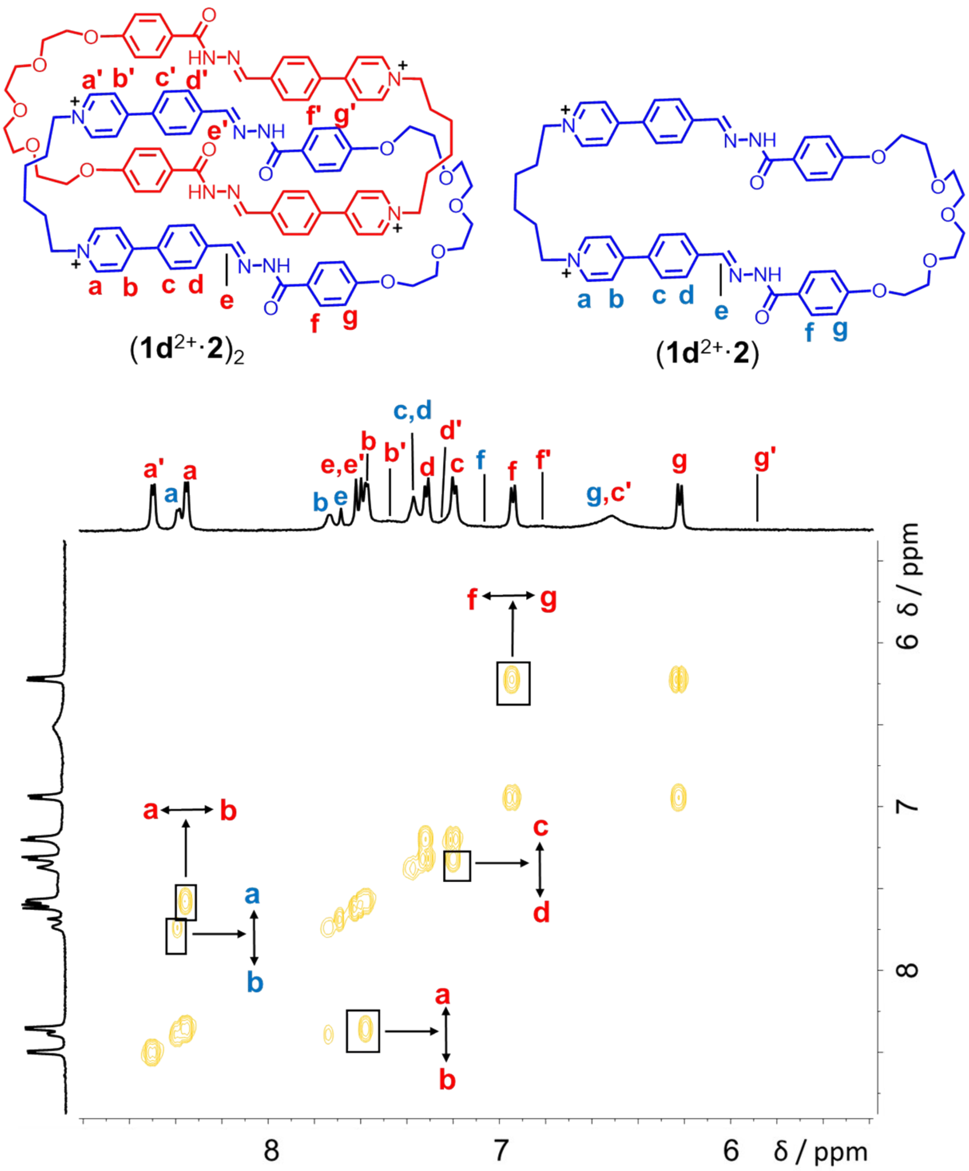


**Figure S31.** $^1$H−$^1$H COSY spectrum (500 MHz, $D_2O$, 298 K) of the reaction mixture by condensing a 1:1 mixture of **1d**$^{2+}$2Br$^-$ (4.5 mM) and **2** (4.5 mM). Counteranions could be either Cl$^-$ or Br$^-$. Key correlation peaks are labeled in the spectrum.

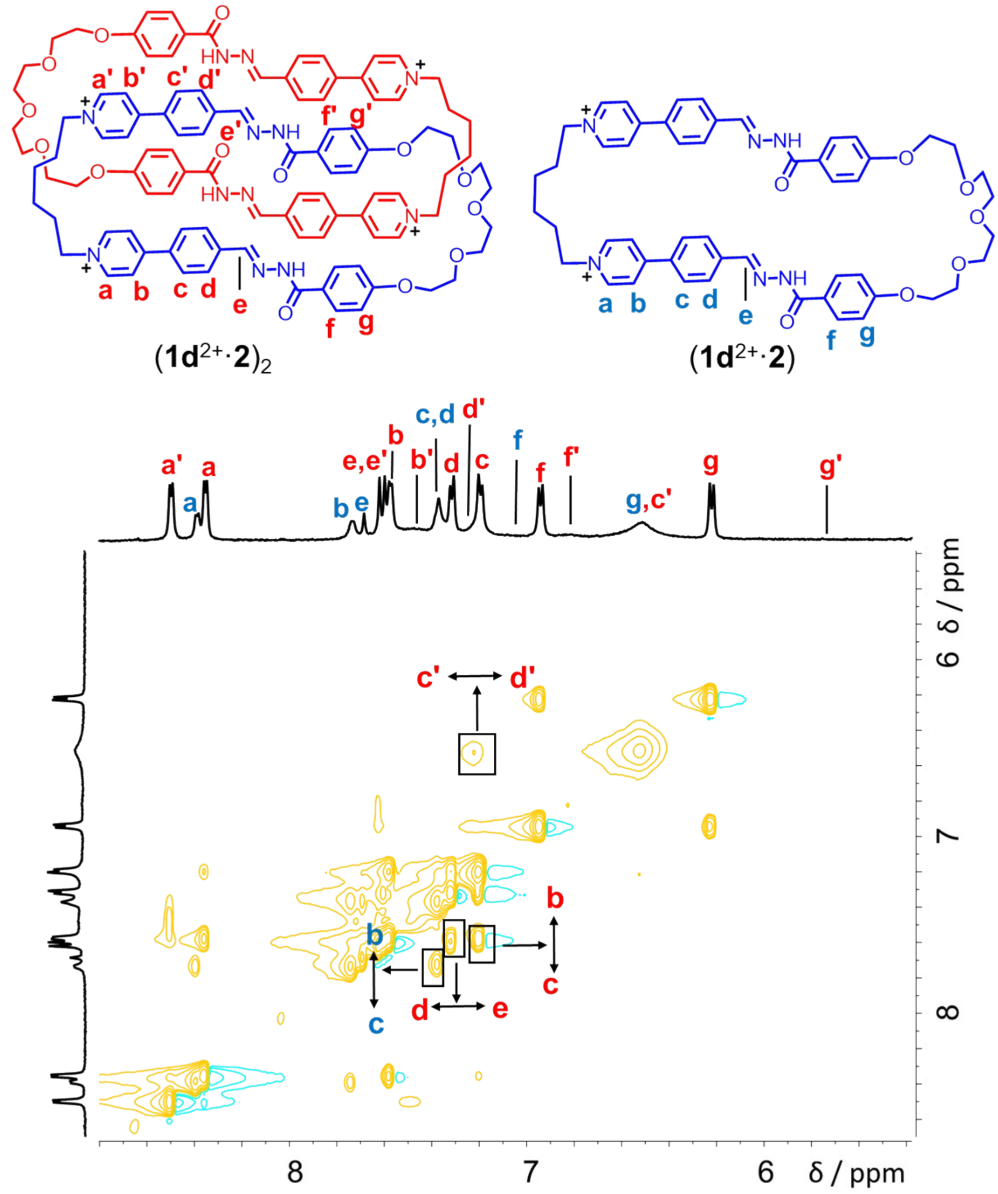

**Figure S32.** $^1H-^1H$ NOESY spectrum (500 MHz, $D_2O$, 298 K) of the reaction mixture by condensing a 1:1 mixture of $\mathbf{1d}^{2+}2Br^-$ (4.5 mM) and **2** (4.5 mM). Counteranions could be either $Cl^-$ or $Br^-$. Key correlation peaks are labeled in the spectrum.

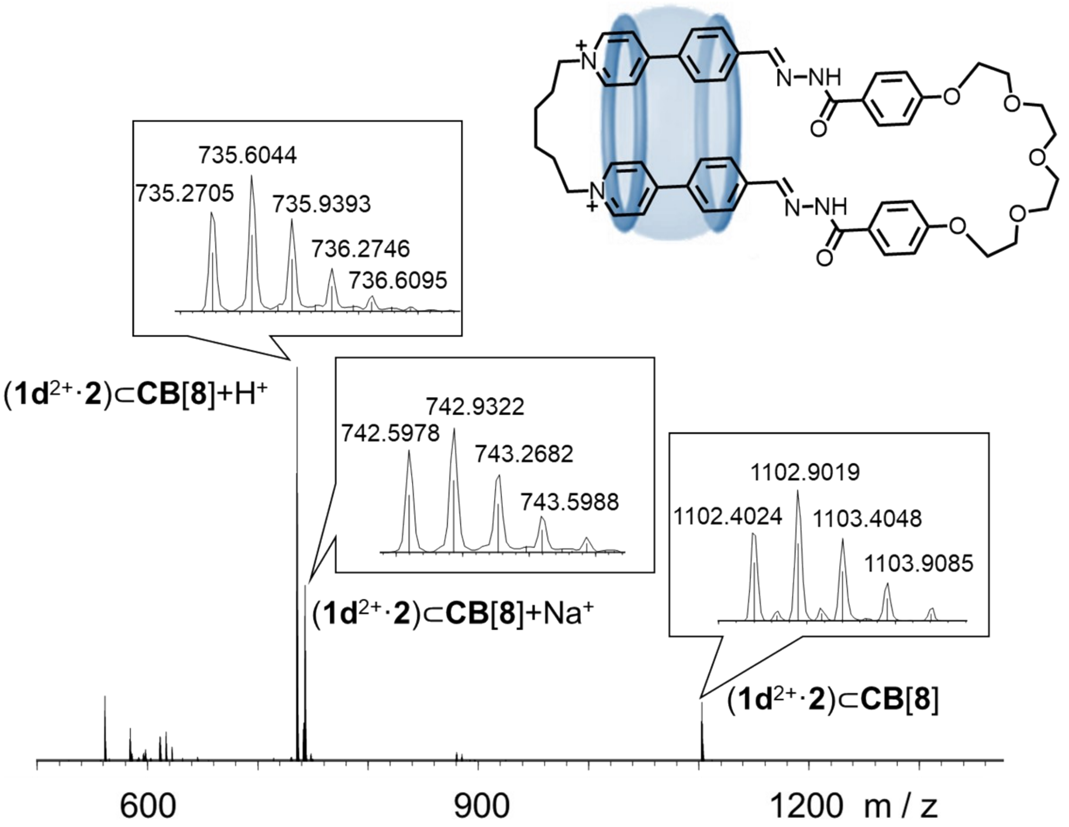


**Figure S33.** High-resolution LCMS-IT-TOF of (**1d**$^{2+}$·**2**)⊂**CB**[**8**]. Counteranions could be either $Cl^-$ or $Br^-$. The signals labeled in the spectrum correspond to molecular cations that contain three and two positive charges, respectively. *m/z* [(**1d**$^{2+}$·**2**)⊂**CB**[**8**] + $Na^+$]$^{3+}$ calculated for $C_{100}H_{104}N_{38}NaO_{23}{}^{3+}$: 742.9350; found: 742.9322. [(**1d**$^{2+}$·**2**)⊂**CB**[**8**] + $H^+$]$^{3+}$ calculated for $C_{100}H_{105}N_{38}O_{23}{}^{3+}$: 735.6077; found: 735.6044. [(**1d**$^{2+}$·**2**)⊂**CB**[**8**]]$^{2+}$ calculated for $C_{100}H_{104}N_{38}O_{23}{}^{2+}$: 1102.9080; found: 1102.9019.

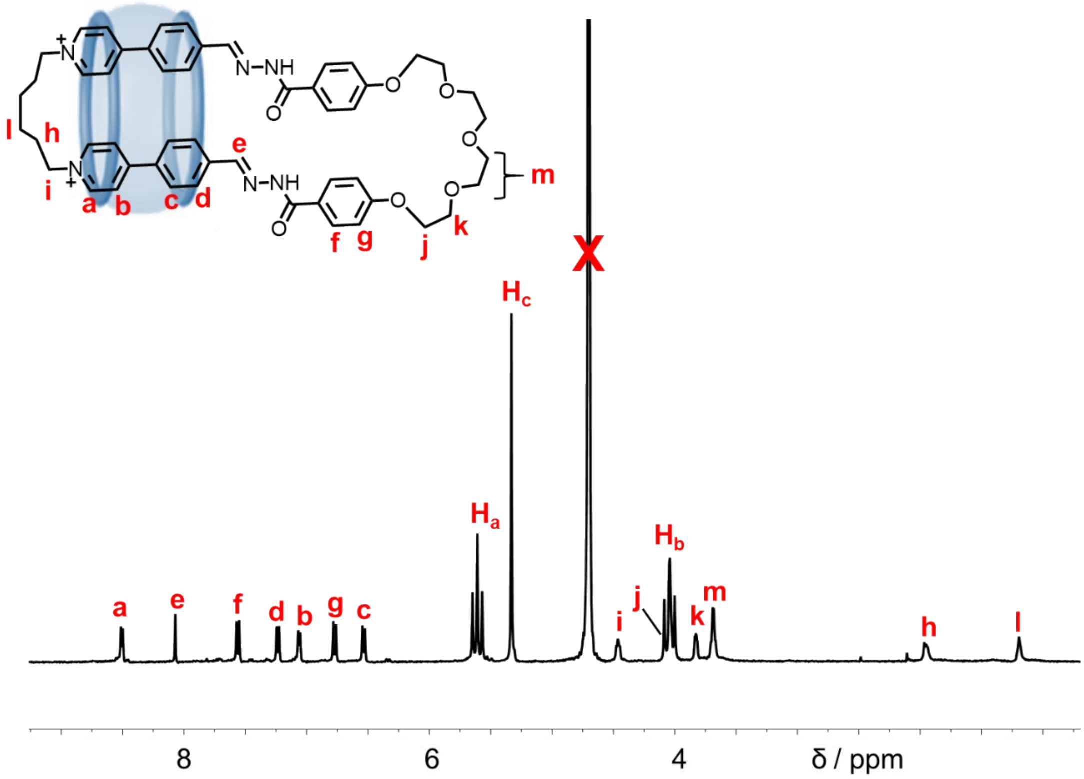


**Figure S34.** Partial $^{1}$H NMR spectrum of (**1d**$^{2+}$·**2**)⊂**CB[8]** (500 MHz, $D_2O$, 298 K). Counteranions could be either $Cl^-$ or $Br^-$.

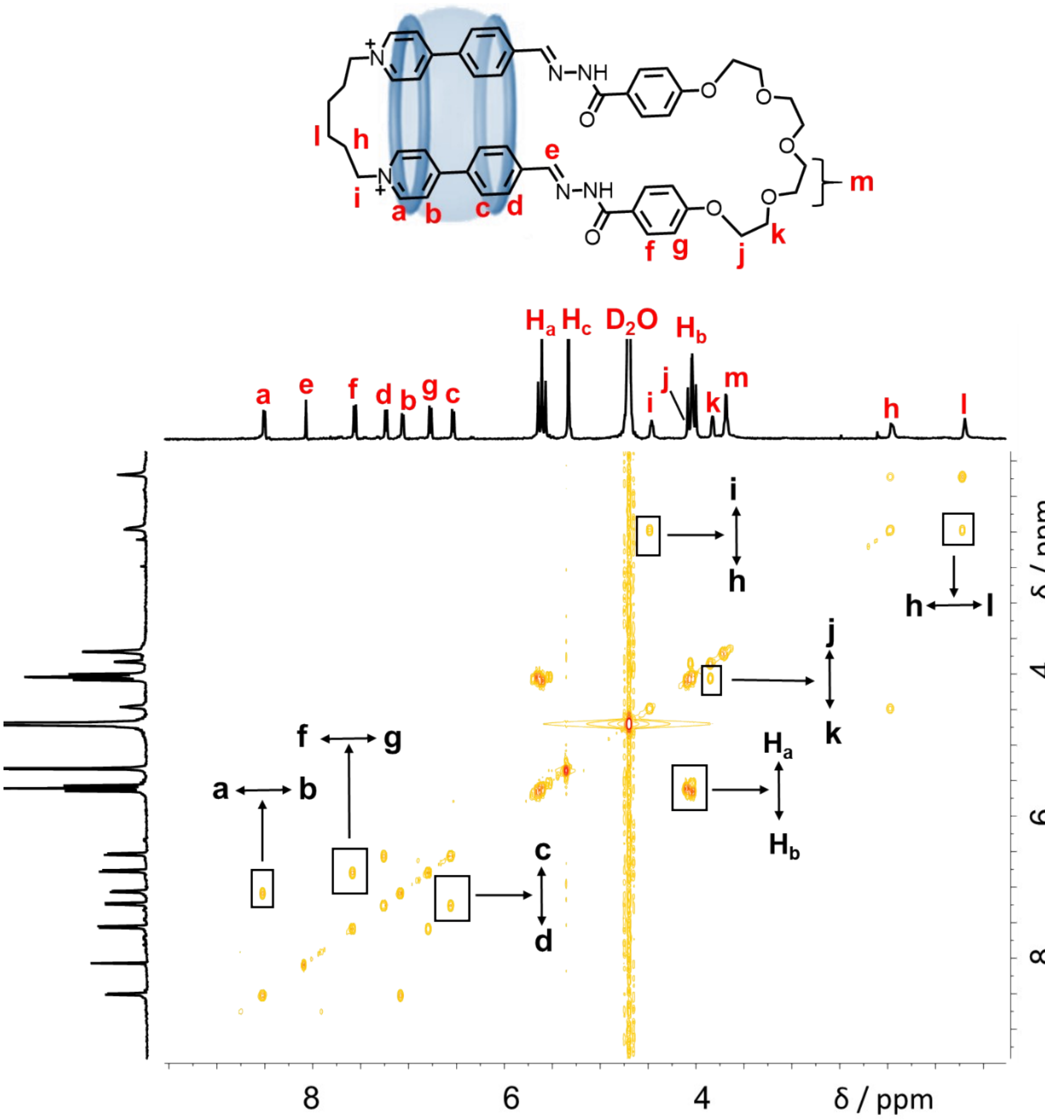


**Figure S35.** $^{1}H-^{1}H$ COSY spectrum (500 MHz, $D_2O$, 298 K) of (**1d**$^{2+}$·**2**)⊂**CB**[**8**]. Counteranions could be either $Cl^-$ or $Br^-$. Key correlation peaks are labeled in the spectrum.

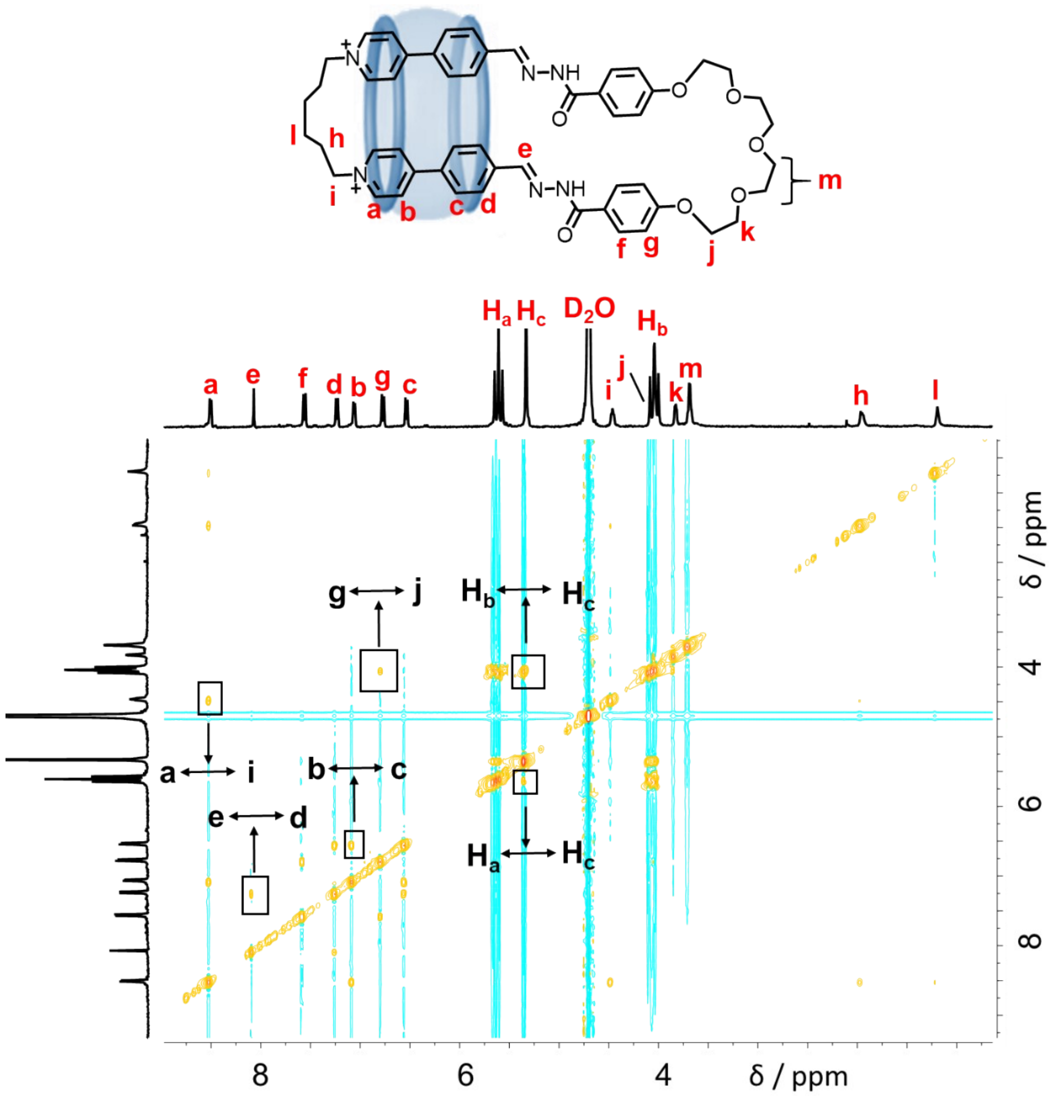


**Figure S36.** $^1H-^1H$ NOESY spectrum (500 MHz, $D_2O$, 298 K) of (**1d**$^{2+}$·**2**)⊂**CB[8]**. Counteranions could be either $Cl^-$ or $Br^-$. Key correlation peaks are labeled in the spectrum.

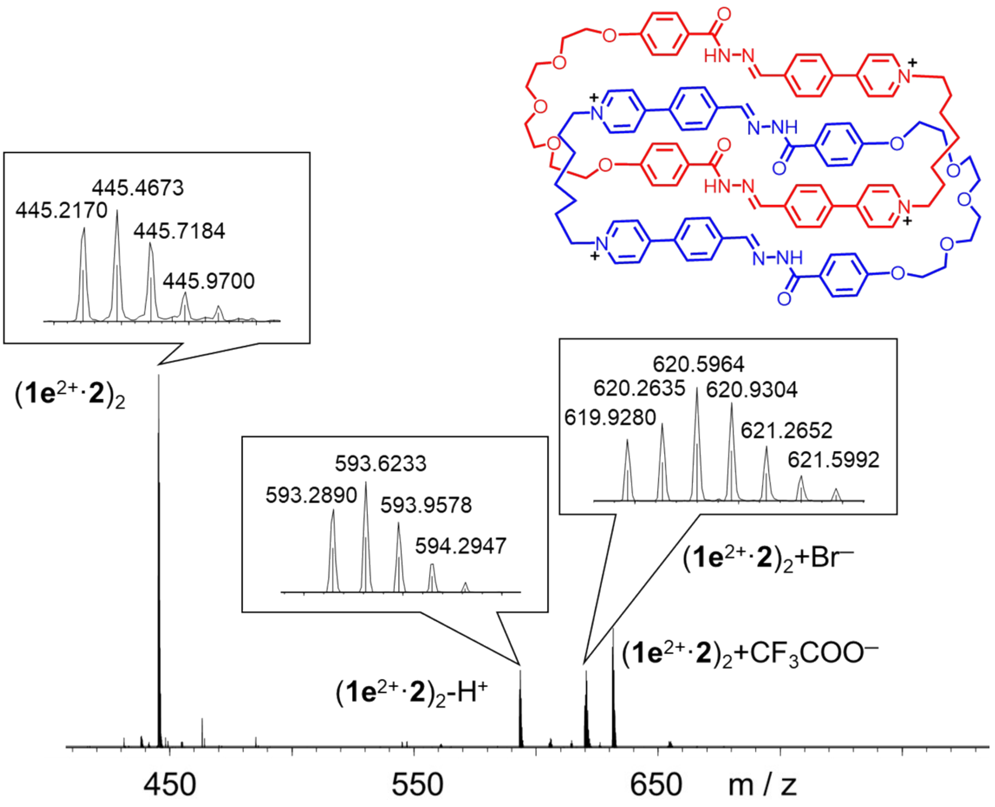


**Figure S37.** High-resolution LCMS-IT-TOF of [2]catenane ($\mathbf{1e}^{2+}\cdot\mathbf{2}$)$_2$. Counteranions could be either $Cl^-$ or $Br^-$. The signals labeled in the spectrum correspond to molecular cations that contain four and three positive charges, respectively. *m/z* $[(\mathbf{1e}^{2+}\cdot\mathbf{2})_2]^{4+}$ calculated for $C_{106}H_{116}N_{12}O_{14}^{3+}$: 445.4686; found: 445.4673. $[(\mathbf{1e}^{2+}\cdot\mathbf{2})_2 - H^+]^{3+}$ calculated for $C_{106}H_{115}N_{12}O_{14}^{3+}$: 593.6224; found: 593.6233. $[(\mathbf{1e}^{2+}\cdot\mathbf{2})_2 + Br^-]^{3+}$ calculated for $C_{106}H_{116}BrN_{12}O_{14}^{4+}$: 620.2645; found: 620.2635.

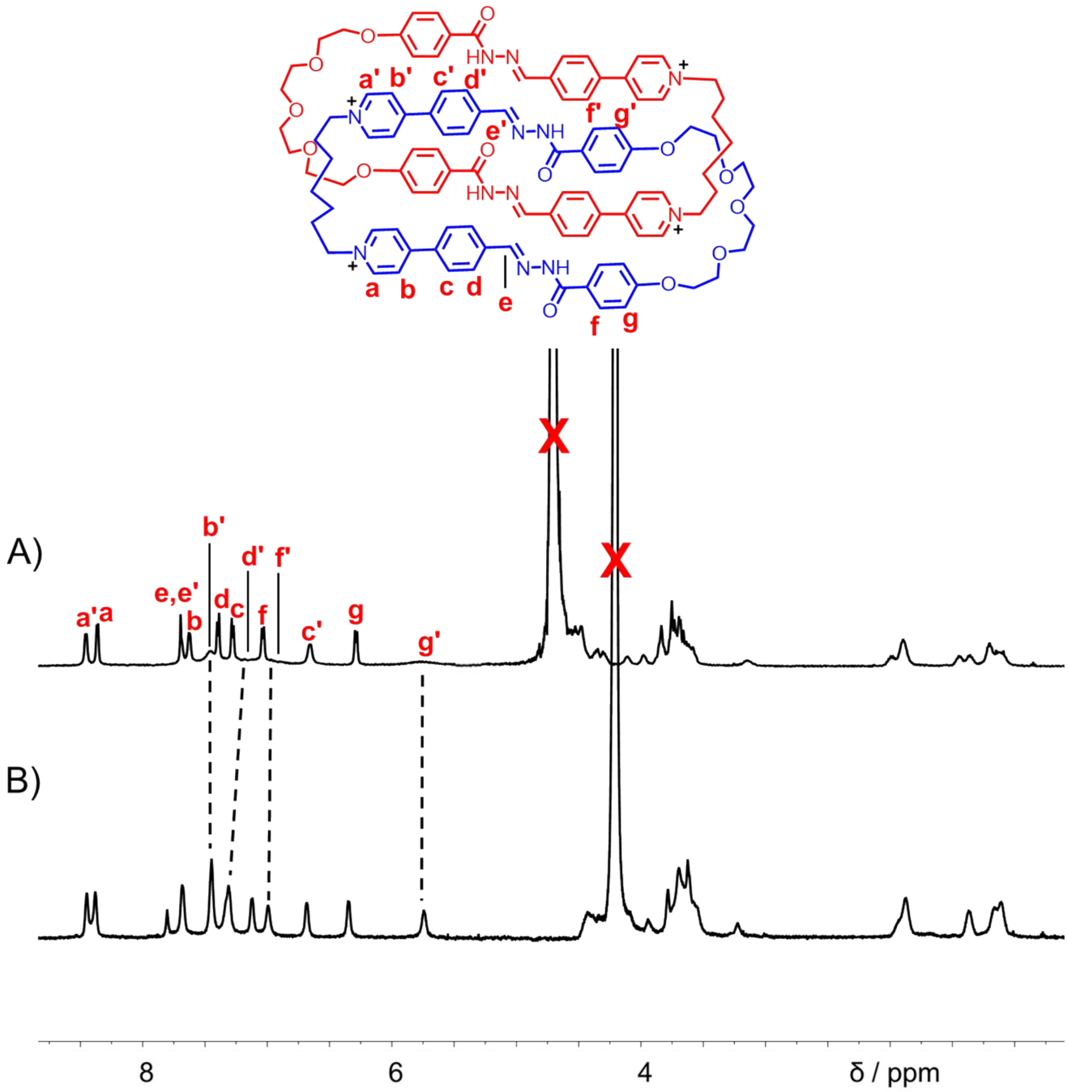


**Figure S38.** Partial $^{1}H$ NMR spectra of A) ($\mathbf{1e}^{2+}\cdot\mathbf{2}$)$_2$ (600 MHz, $D_2O$, 298 K), B) ($\mathbf{1e}^{2+}\cdot\mathbf{2}$)$_2$ (600 MHz, $D_2O$, 333 K). Counteranions could be either $Cl^{-}$ or $Br^{-}$.

The $^{1}H$ NMR spectra of ($\mathbf{1e}^{2+}\cdot\mathbf{2}$)$_2\cdot4Cl^{-}$ are temperature dependent in $D_2O$. At room temperature (25 °C), the resonances of the protons b', d', c', f' and g' (see Figure S38) are relatively broad. This observation could be explained by the fact that, within the cavity of one of the two interlocked rings, the circumvolution movement corresponding parts is significantly slowed down. At elevated temperature such as 60 °C, these peaks become sharper, indicating that the circumvolution motion is "speeded up" at higher temperature.

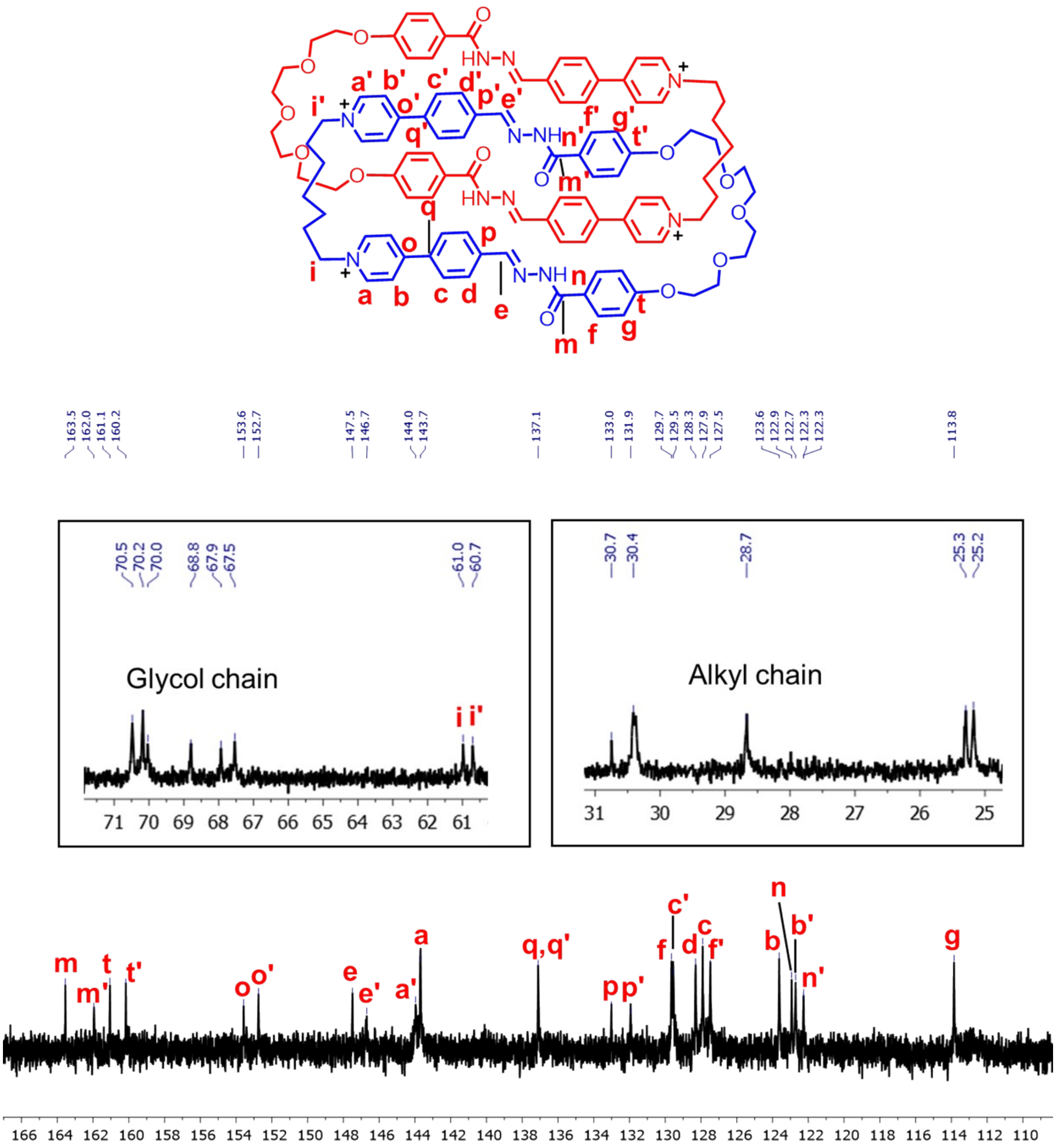

**Figure S39.** $^{13}$C NMR spectrum (150 M Hz, 298 K, $D_2O$:DMSO = 4:3) of (**1e**$^{2+}$·**2**)$_2$. Counteranions could be either $Cl^-$ or $Br^-$.

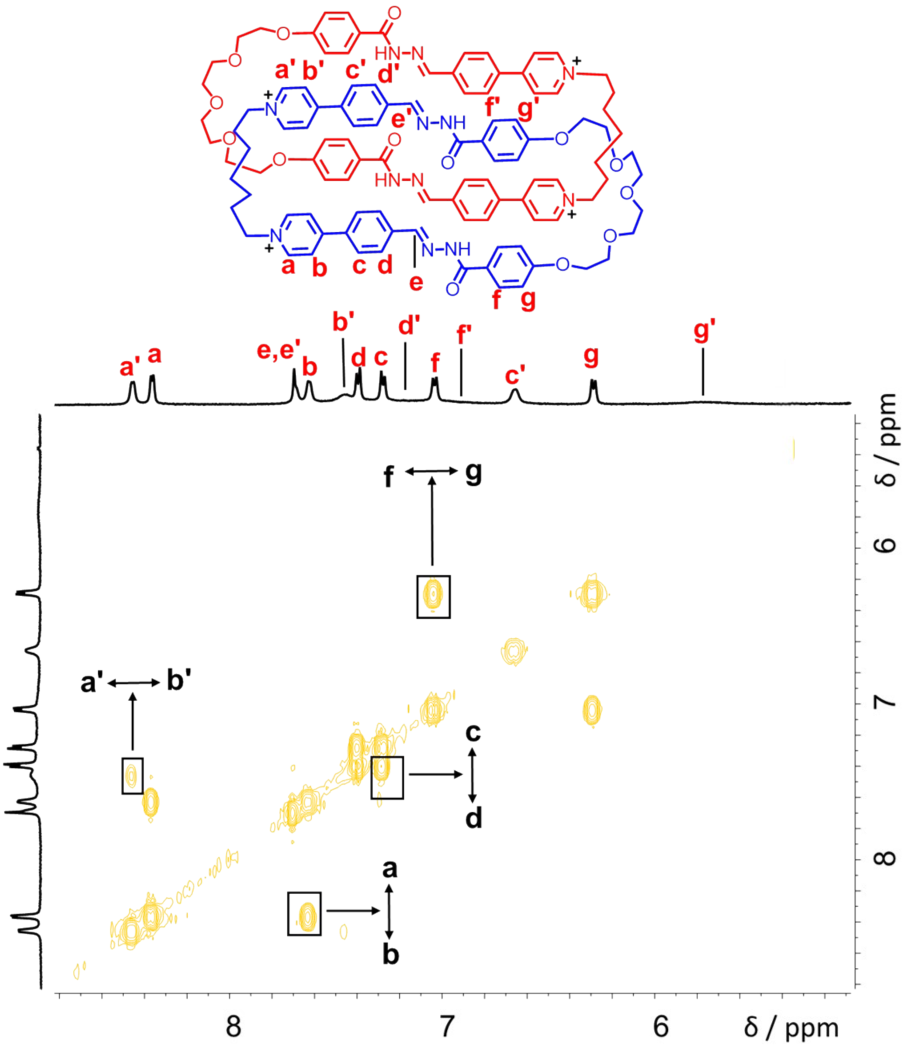


**Figure S40.** $^{1}H-^{1}H$ COSY spectrum (500 MHz, $D_2O$, 298 K) of [2]catenane $(\mathbf{1e}^{2+}\cdot\mathbf{2})_2$. Counteranions could be either $Cl^-$ or $Br^-$. Key correlation peaks are labeled in the spectrum.

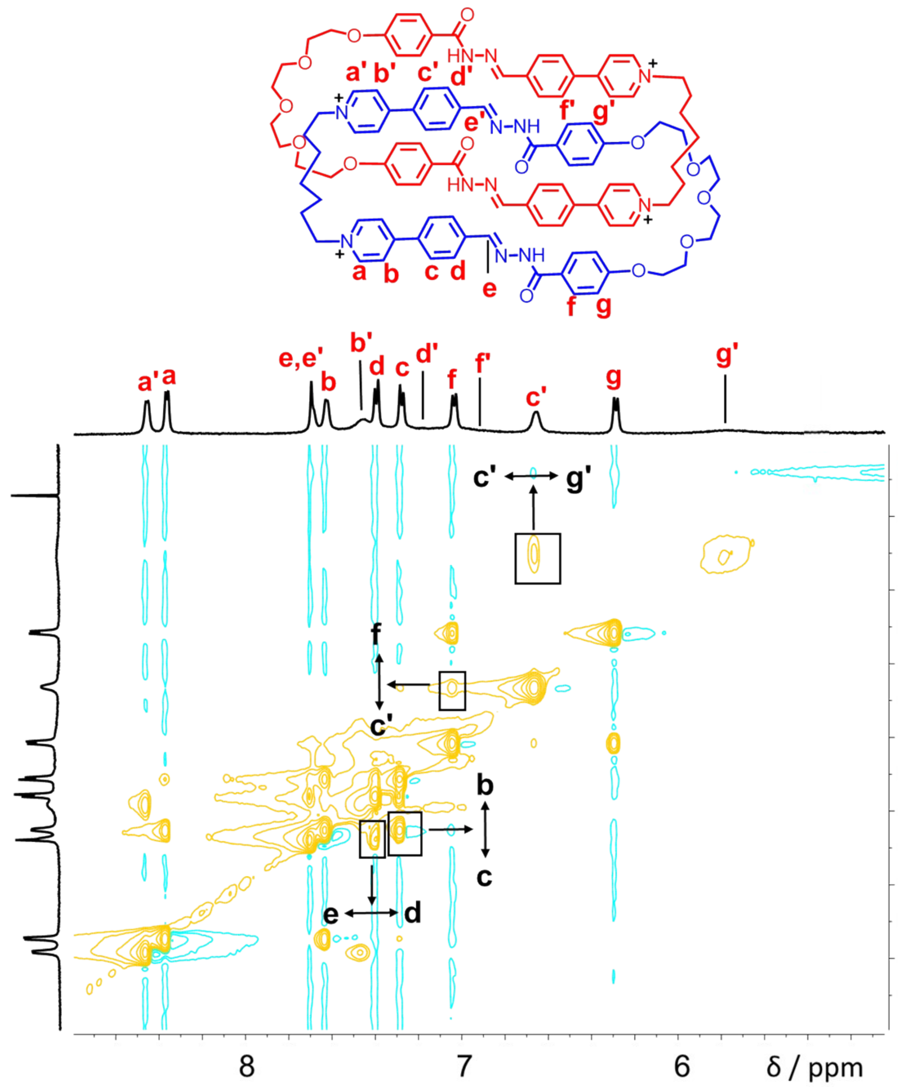

**Figure S41.** ${}^{1}H-{}^{1}H$ NOESY spectrum (500 MHz, $D_2O$, 298 K) of [2]catenane ($\mathbf{1e}^{2+}\cdot\mathbf{2}$)$_2$. Counteranions could be either $Cl^-$ or $Br^-$. Key correlation peaks are labeled in the spectrum.

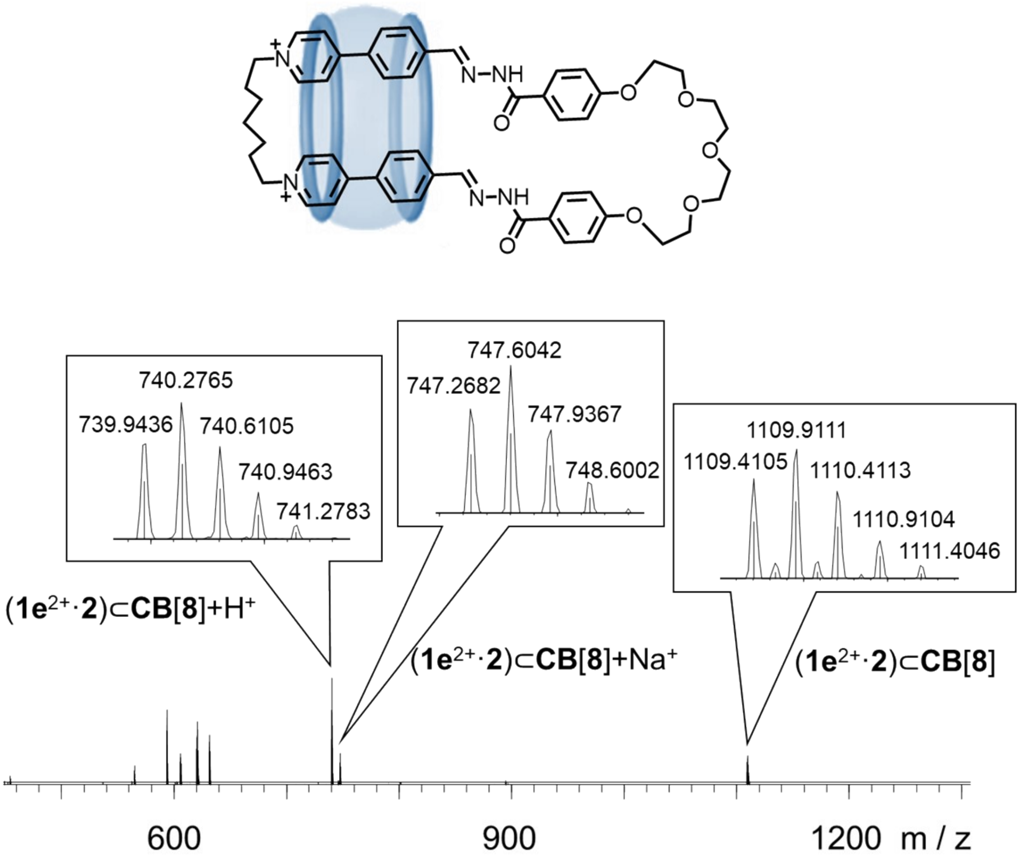


**Figure S42.** High-resolution LCMS-IT-TOF of (**1e**$^{2+}$·**2**)⊂**CB**[**8**]. Counteranions could be either $Cl^-$ or $Br^-$.The signals labeled in the spectrum correspond to molecular cations that contain four, three and two positive charges, respectively. *m/z* [(**1e**$^{2+}$·**2**)⊂**CB[8]** + $Na^+$]$^{3+}$ calculated for $C_{101}H_{106}N_{38}NaO_{23}{}^{3+}$: 747.6069; found: 747.6042. [(**1e**$^{2+}$·**2**)⊂**CB[8]** + $H^+$]$^{3+}$ calculated for $C_{101}H_{107}N_{38}O_{23}{}^{3+}$: 740.2796; found: 740.2765. [(**1e**$^{2+}$·**2**)⊂**CB[8]**]$^{2+}$ calculated for $C_{101}H_{106}N_{38}O_{23}{}^{2+}$: 1109.9158; found: 1109.9111.

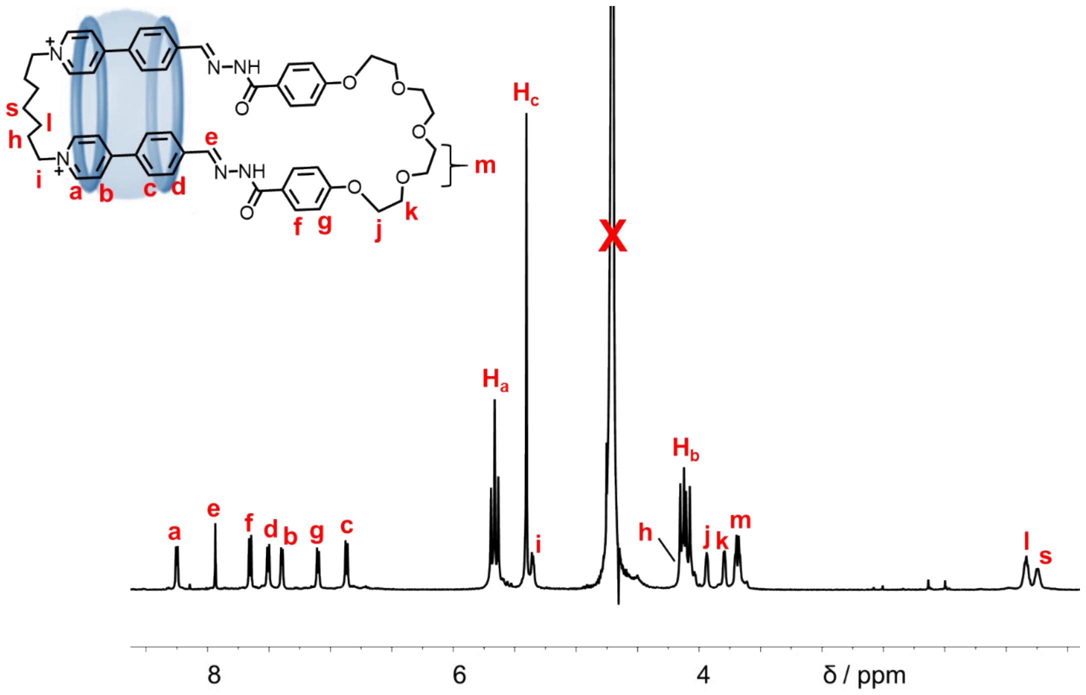


**Figure S43.** Partial $^1$H NMR spectrum of (**1e**$^{2+}$·**2**)⊂**CB[8]** (500 MHz, $D_2O$, 298 K). Counteranions could be either $Cl^-$ or $Br^-$.

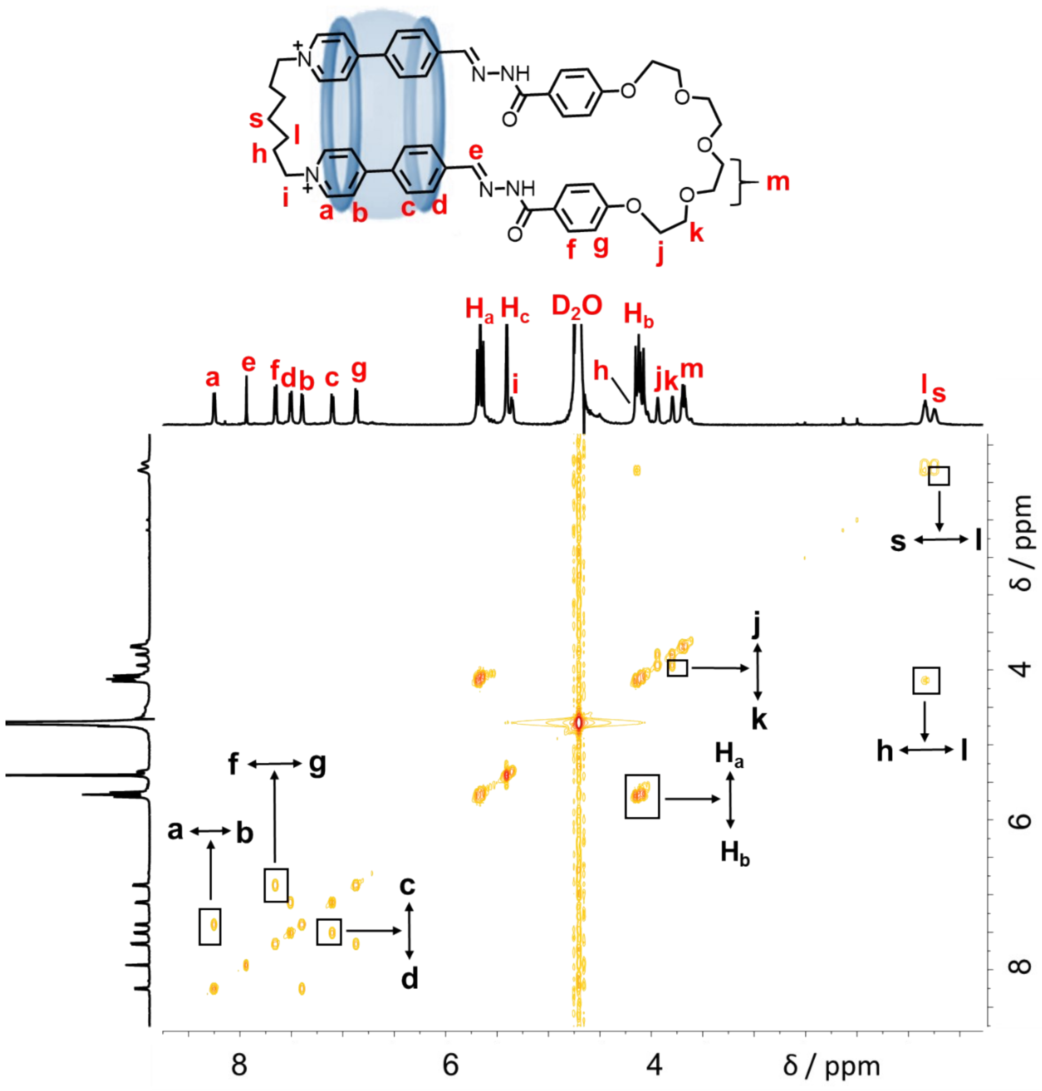


**Figure S44.** $^1$H–$^1$H COSY spectrum (500 MHz, $D_2O$, 298 K) of (**1e**$^{2+}$·**2**)⊂**CB[8]**. Counteranions could be either $Cl^-$ or $Br^-$. Key correlation peaks are labeled in the spectrum.

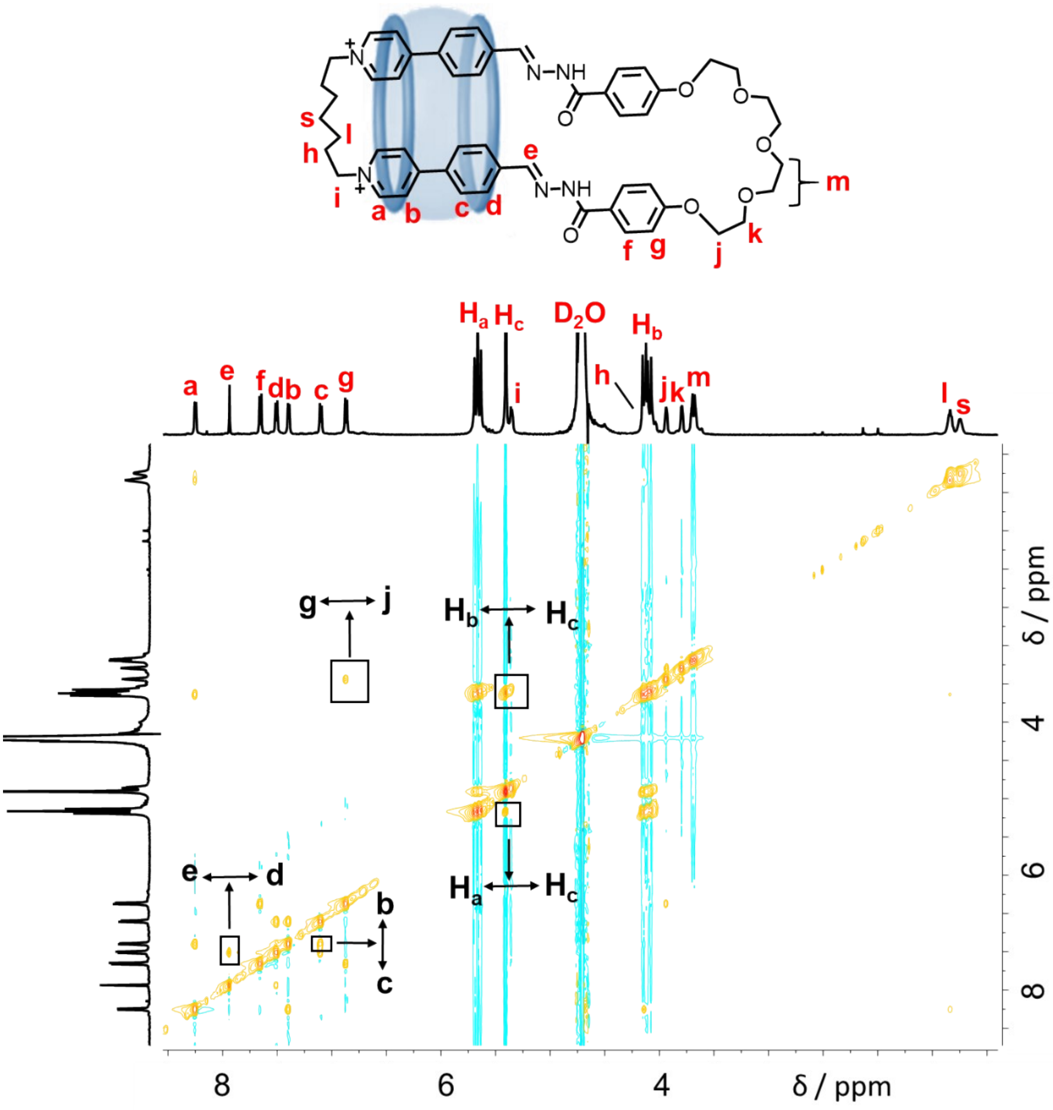



**Figure S45.** $^1H-^1H$ NOESY spectrum (500 MHz, $D_2O$, 298 K) of (**1e**$^{2+}$·**2**)⊂**CB[8]**. Counteranions could be either $Cl^-$ or $Br^-$. Key correlation peaks are labeled in the spectrum.

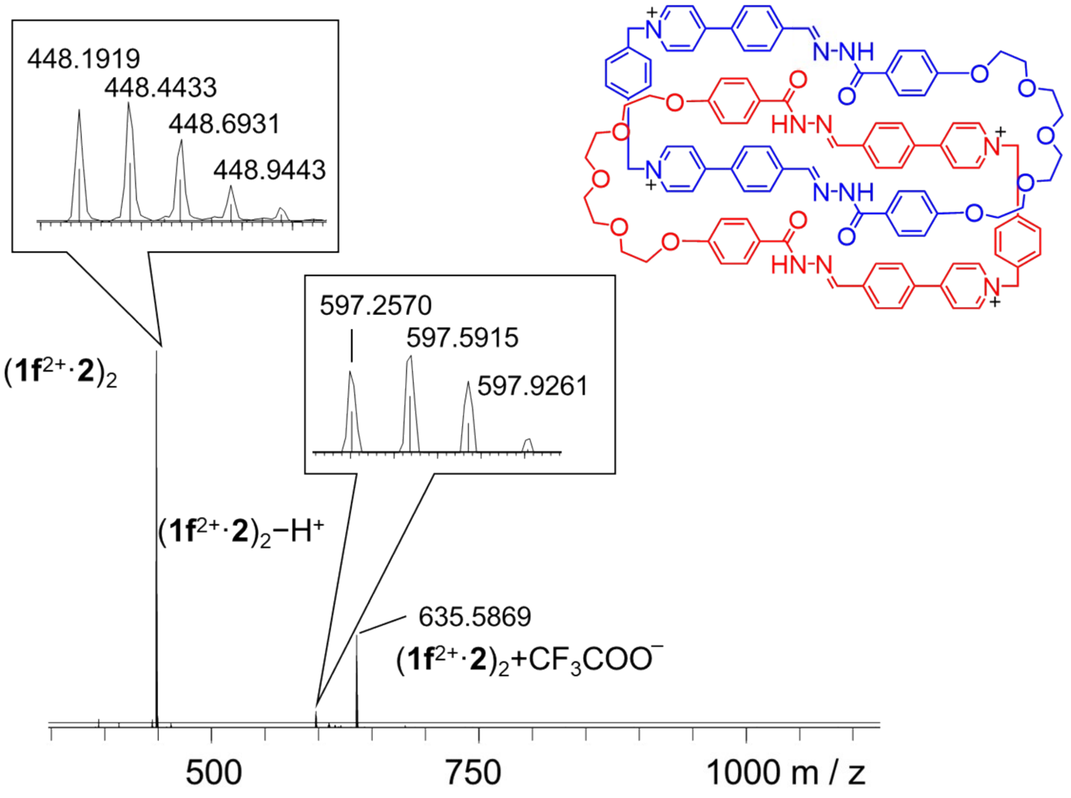


**Figure S46.** High-resolution LCMS-IT-TOF of ($\mathbf{1f}^{2+}\cdot\mathbf{2})_2\cdot 4Cl^-$. The signals labeled in the spectrum correspond to molecular cations that contain four and three positive charges, respectively. *m/z* $[(\mathbf{1f}^{2+}\cdot\mathbf{2})_2]^{4+}$ calculated for $C_{108}H_{104}N_{12}O_{14}{}^{4+}$: 448.4452; found: 448.4433. $[(\mathbf{1f}^{2+}\cdot\mathbf{2})_2 - H^+]^{3+}$ calculated for $C_{108}H_{103}N_{12}O_{14}{}^{3+}$: 597.5911; found: 597.5915.

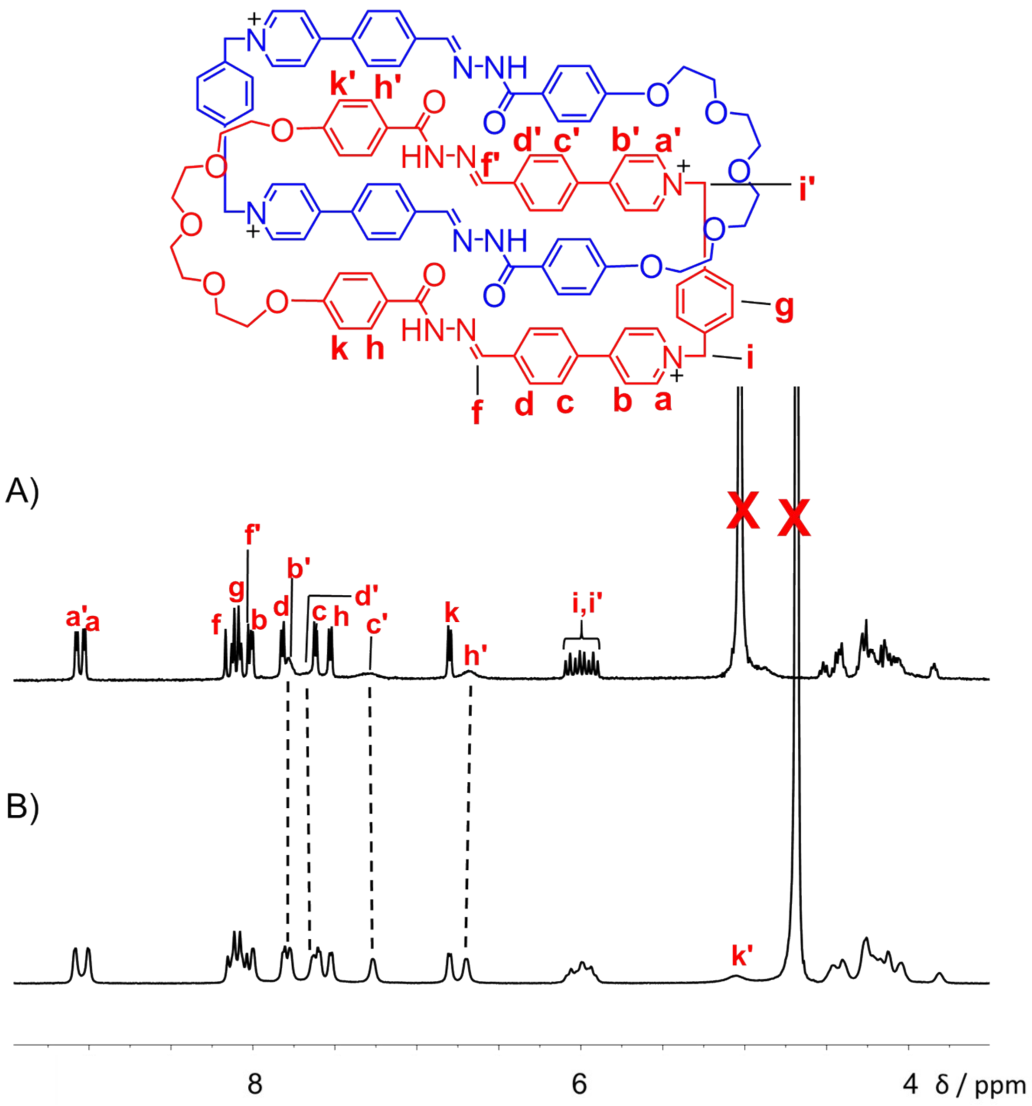


**Figure S47.** Partial $^1$H NMR spectra of A) $(\mathbf{1f}^{2+}\cdot\mathbf{2})_2\cdot 4Cl^-$ (600 MHz, $D_2O$, 298 K), B) $(\mathbf{1f}^{2+}\cdot\mathbf{2})_2\cdot 4Cl^-$ (600 MHz, $D_2O$, 333 K).

The $^1$H NMR spectra of $(\mathbf{1f}^{2+}\cdot\mathbf{2})_2\cdot 4Cl^-$ are temperature dependent in $D_2O$. At room temperature (25 $^oC$), the resonances of the protons b', d', c', h' and k' (see Figure S47) are relatively broad. This observation could be explained by the fact that, within the cavity of one of the two interlocked rings, the circumvolution movement corresponding parts is significantly slowed down. At elevated temperature such as 60 $^oC$, these peaks become sharper, indicating that the circumvolution motion is "speeded up" at higher temperature.

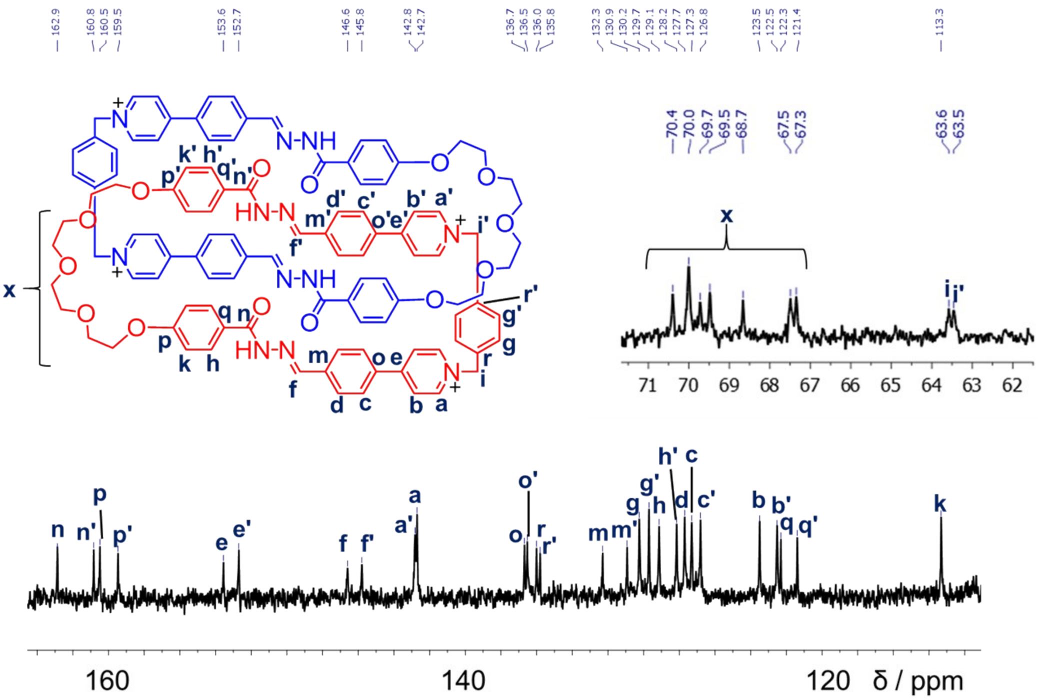

**Figure S48.** $^{13}C$ NMR spectrum (125 M Hz, 298 K, $D_2O$:DMSO = 4:3) of ($\mathbf{1f}^{2+}\cdot\mathbf{2}$)$_2\cdot$4Cl$^-$.

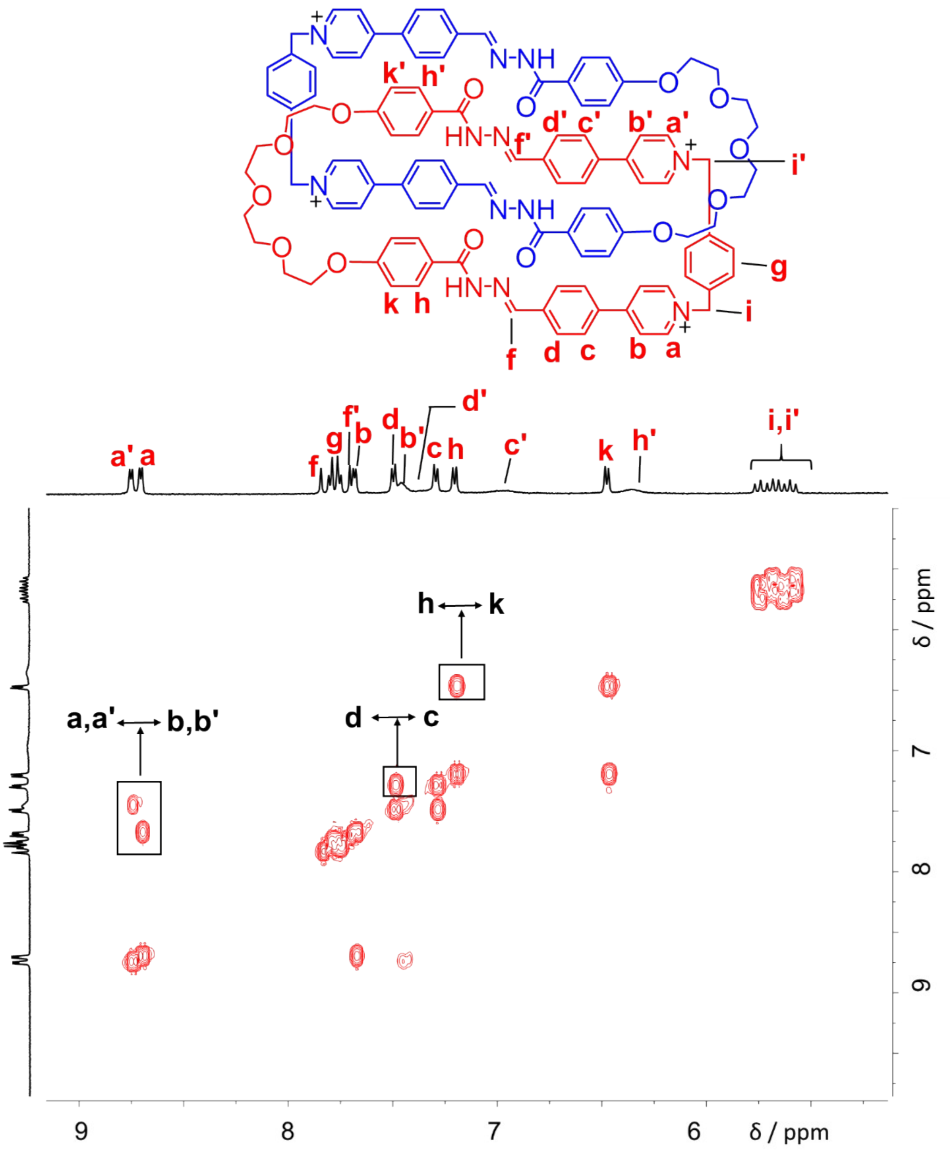


**Figure S49.** ¹H−¹H COSY spectrum (500 MHz, $D_2O$, 298 K) of (**1f**$^{2+}$·**2**)$_2$·4Cl$^-$. Key correlation peaks are labeled in the spectrum.

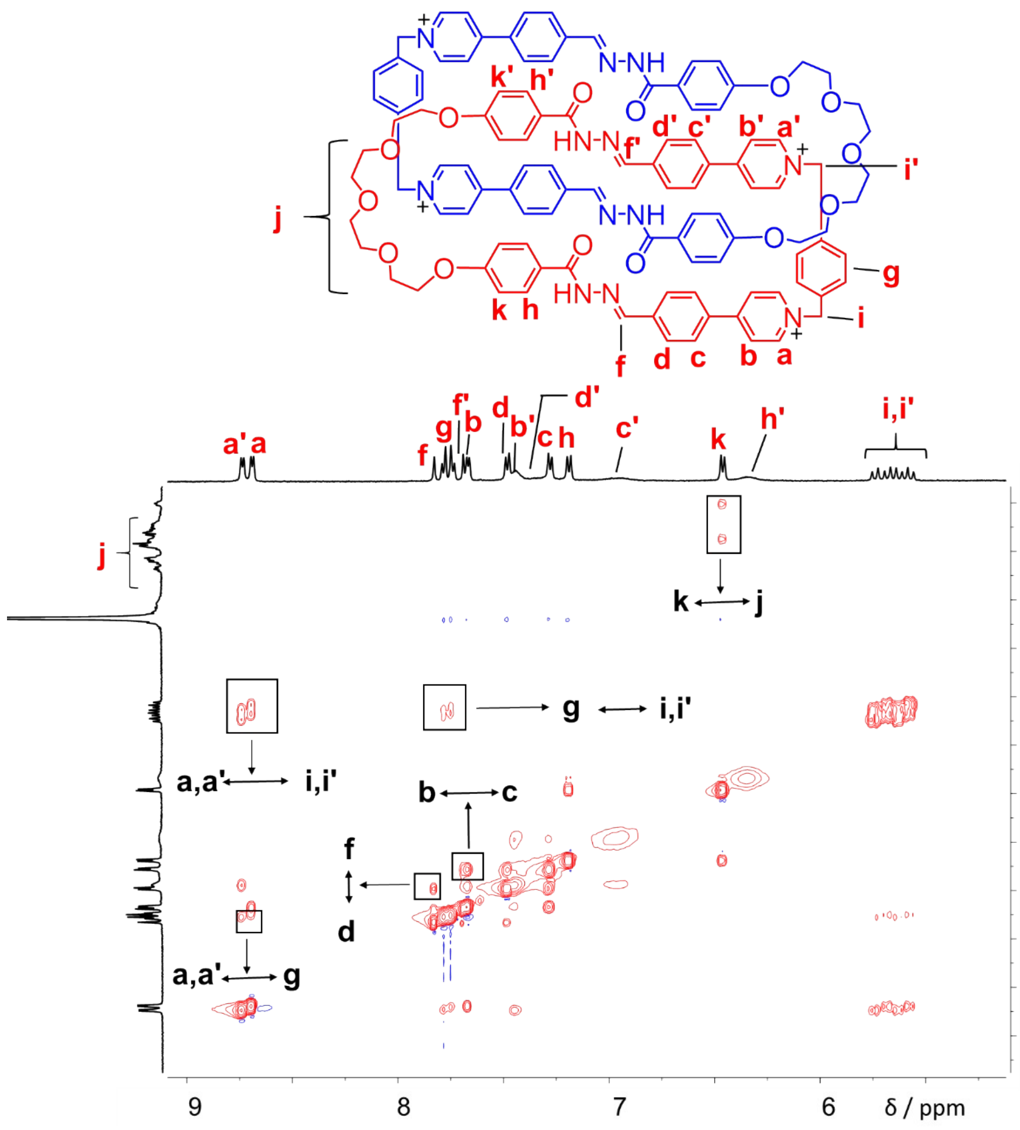


**Figure S50.** $^1H-^1H$ NOESY spectrum (500 MHz, $D_2O$, 298 K) of $(\mathbf{1f}^{2+}\cdot\mathbf{2})_2\cdot4Cl^-$. Key correlation peaks are labeled in the spectrum.

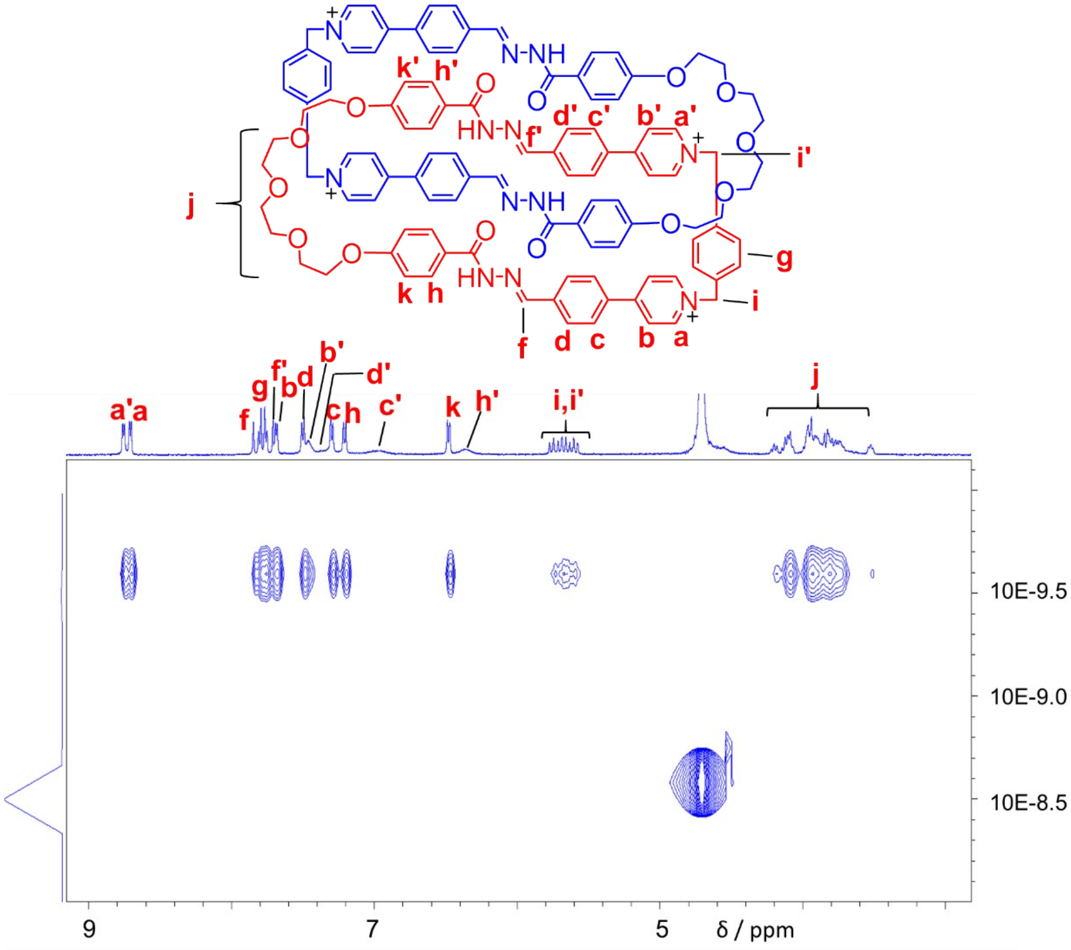


**Figure S51.** DOSY spectrum (500 MHz, $D_2O$, 298 K) of ($\mathbf{1f}^{2+}\cdot\mathbf{2}$)$_2\cdot 4Cl^-$.

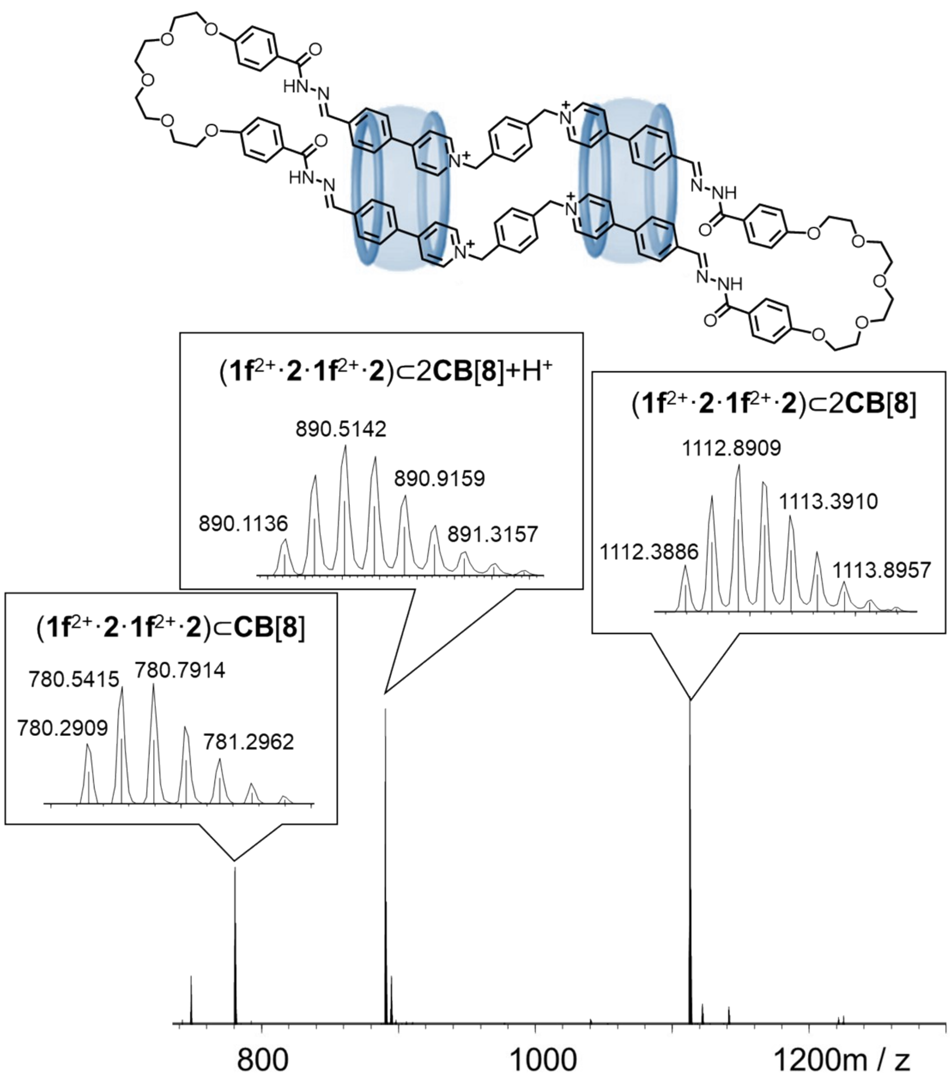


**Figure S52.** High-resolution LCMS-IT-TOF of (**1f**$^{2+}$·**2**·**1f**$^{2+}$·**2**)⊂2**CB**[8]. Charges are balanced by chloride counteranions. The signals labeled in the spectrum correspond to molecular cations that contain five and four positive charges, respectively. *m/z* [(**1f**$^{2+}$·**2**·**1f**$^{2+}$·**2**)⊂**CB**[8]]$^{4+}$ calculated for $C_{156}H_{152}N_{44}O_{30}{}^{4+}$: 780.5433; found: 780.5415. [(**1f**$^{2+}$·**2**·**1f**$^{2+}$·**2**)⊂2**CB**[8]]$^{4+}$ calculated for $C_{204}H_{200}N_{76}O_{46}{}^{4+}$: 1112.8923; found: 1112.8909. [(**1f**$^{2+}$·**2**·**1f**$^{2+}$·**2**)⊂2**CB**[8]+H$^{+}$]$^{5+}$ calculated for $C_{204}H_{201}N_{76}O_{46}{}^{5+}$: 890.5153; found: 890.5142.

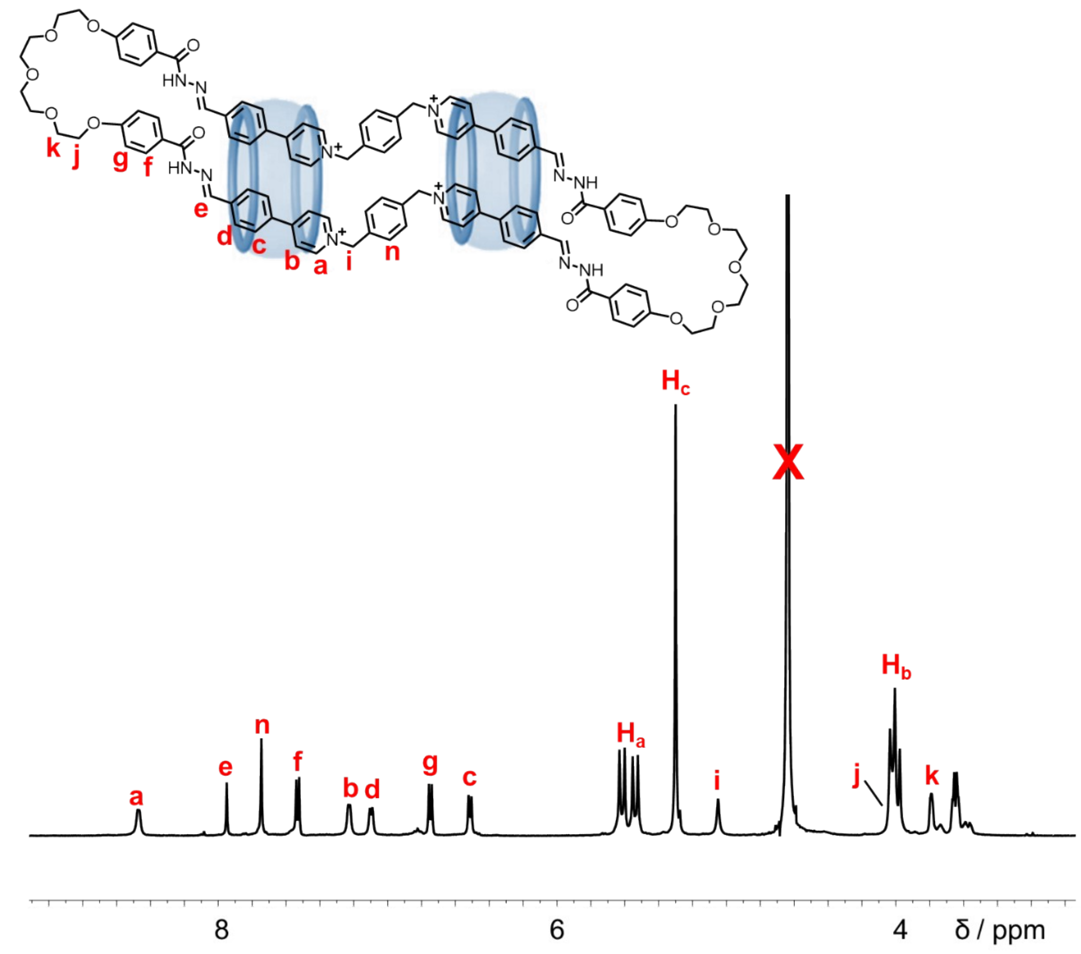


**Figure S53.** Partial $^1$H NMR spectra of (**1f**$^{2+}$·**2**·**1f**$^{2+}$·**2**)⊂2**CB[8]** (500 MHz, $D_2O$, 298 K). Charges are balanced by chloride counteranions.

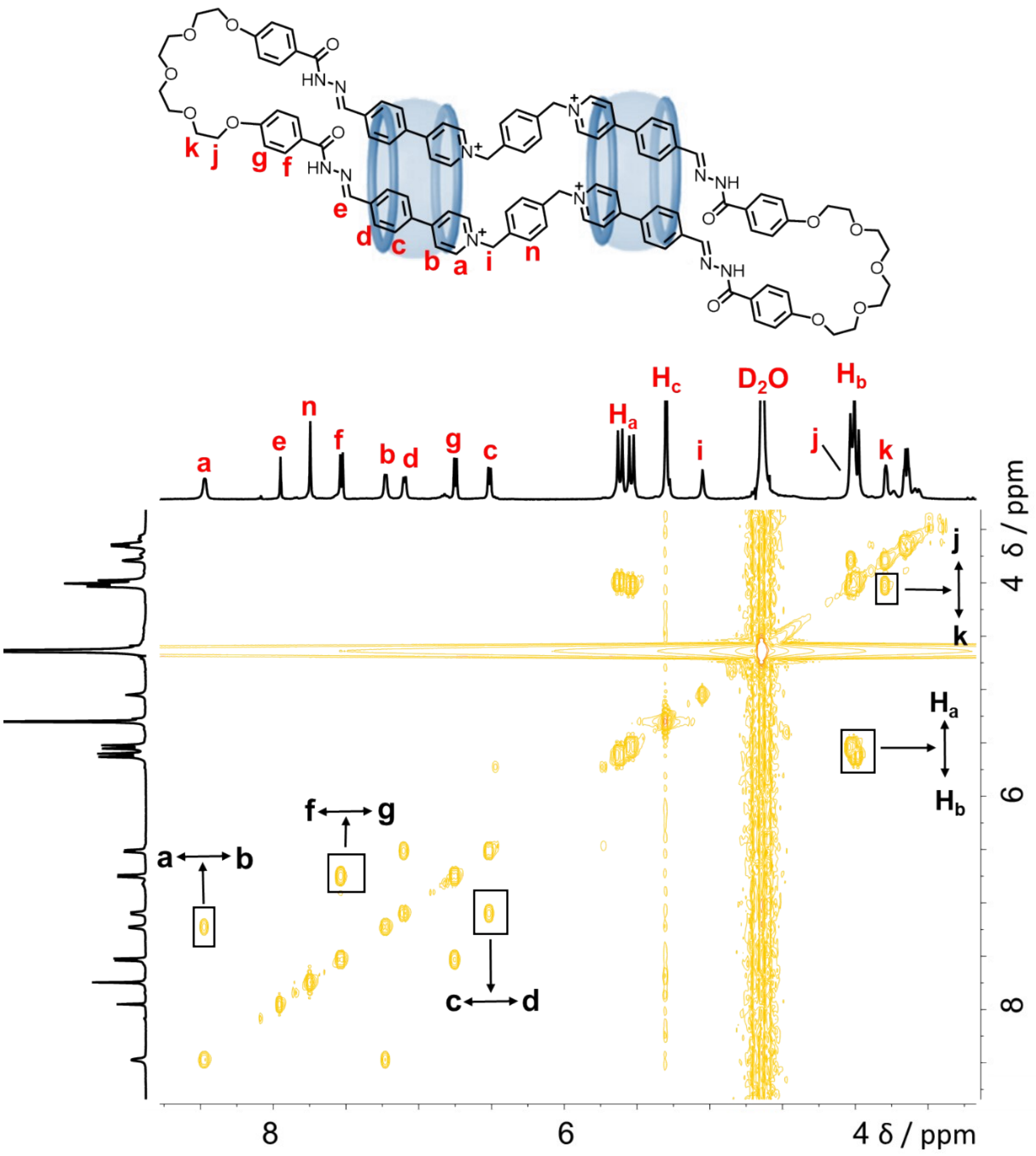


**Figure S54.** $^1$H−$^1$H COSY spectrum (500 MHz, $D_2O$, 298 K) of (**1f**$^{2+}$·**2**·**1f**$^{2+}$·**2**)⊂2**CB[8]**. Charges are balanced by chloride counteranions. Key correlation peaks are labeled in the spectrum.

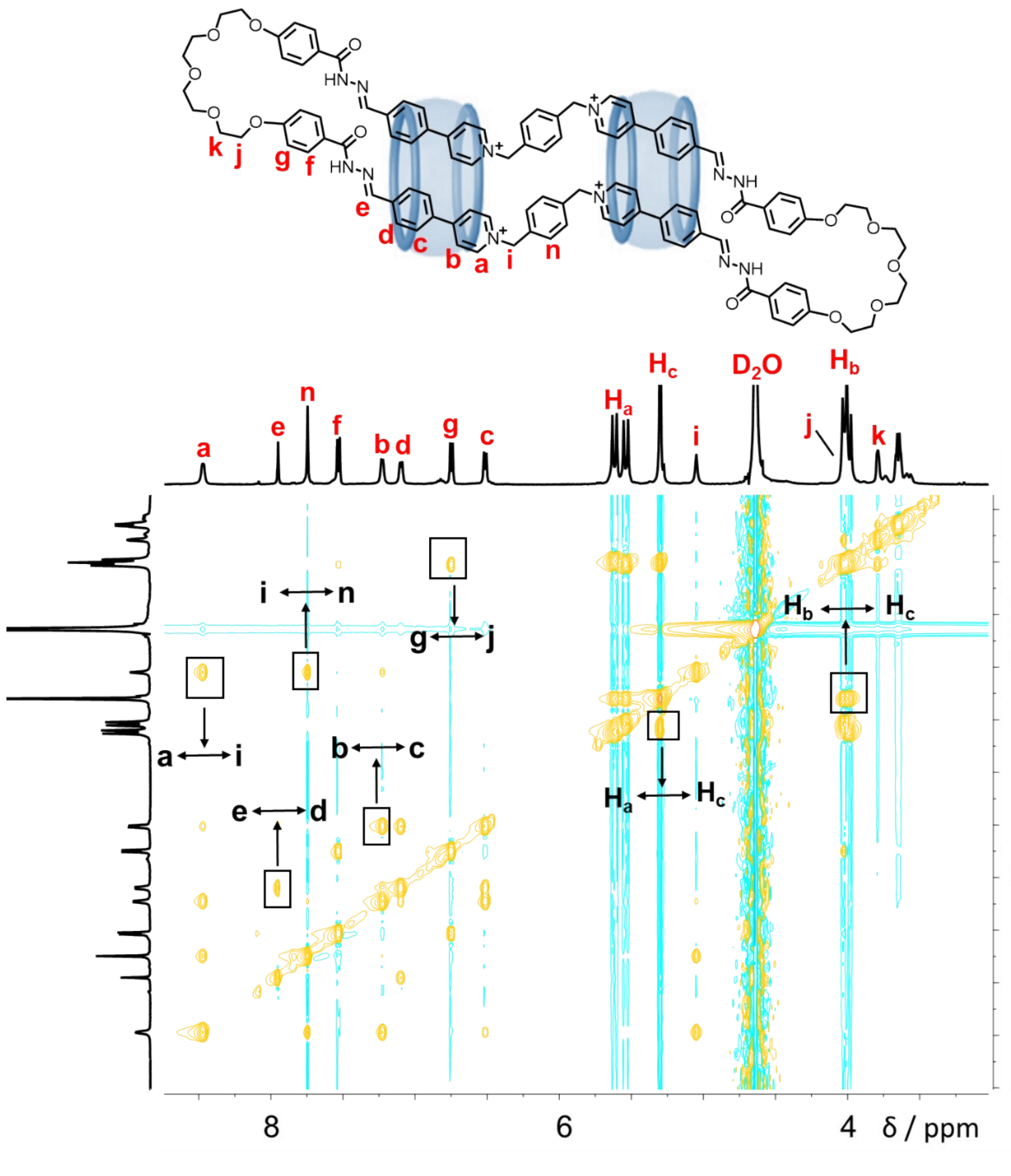


**Figure S55.** $^{1}H-^{1}H$ NOESY spectrum (500 MHz, $D_2O$, 298 K) of (**1f**$^{2+}$·**2**·**1f**$^{2+}$·**2**)⊂2**CB[8]**. Charges are balanced by chloride counteranions. Key correlation peaks are labeled in the spectrum.

## 4. Interconversion between (**1f**$^{2+}$·**2**)$_2$ and (**1f**$^{2+}$·**2**·**1f**$^{2+}$·**2**)⊂2**CB[8]**

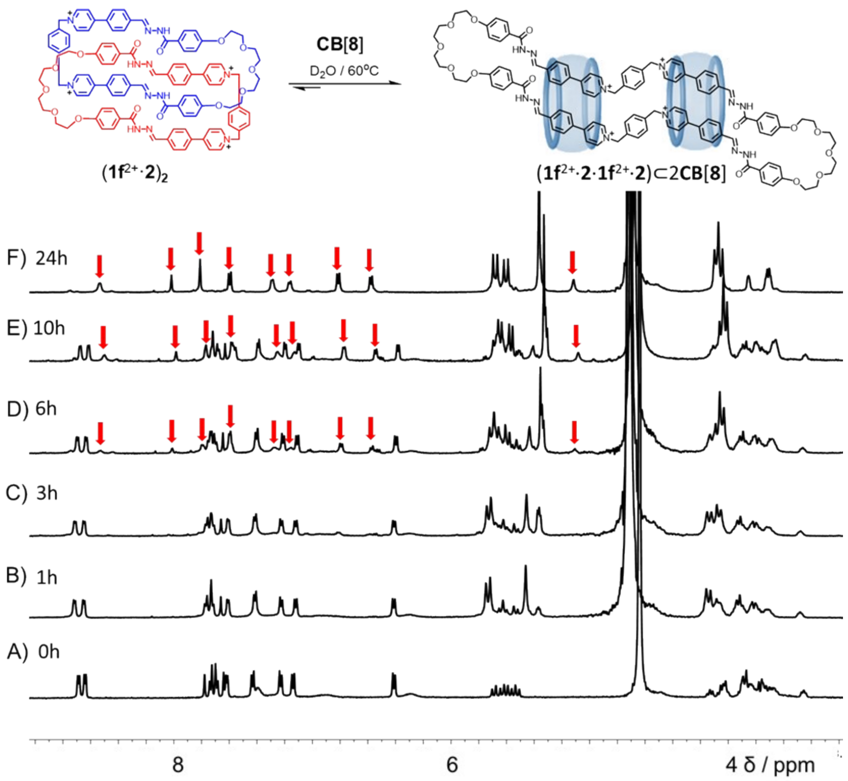


**Figure S56.** The $^1$H NMR spectra (500 MHz, D$_2$O, 298 K) of the [2]catenane (**1f**$^{2+}$·**2**)$_2$, after adding two equiv. of CB[8] for certain amount of time. The resonances corresponding to the [3] pseudorotaxane (**1f**$^{2+}$·**2**·**1f**$^{2+}$·**2**)⊂2**CB**[8] gradually appeared, which are marked with red arrows.

We added two equivalents of **CB**[8] to a D$_2$O solution of [2]catenane (**1f**$^{2+}$·**2**)$_2$ at 60 $^o$C. The reaction progress was monitored by recording its $^1$H NMR spectra in D$_2$O (Figure S56) during the reaction course. $^1$H NMR spectra indicated that, after heating and sonicating the reaction mixture for about 6 h, a new set of sharp resonances was observed in the $^1$H NMR spectrum (Figure S56 D), indicating the formation of a new thermodynamically stable product, namely (**1f**$^{2+}$·**2**·**1f**$^{2+}$·**2**)⊂2**CB**[8]. After approximate 24 h, the resonances corresponding to the [2]catenane (**1f**$^{2+}$·**2**)$_2$ almost completely disappeared, accompanied with the appearance of a set of resonances corresponding to (**1f**$^{2+}$·**2**·**1f**$^{2+}$·**2**)⊂2**CB**[8]. It is noteworthy that during the reaction course, no other reaction intermediates were observed, such as the [2]pseudorotaxane (**1f**$^{2+}$·**2**·**1f**$^{2+}$·**2**)⊂**CB**[8].

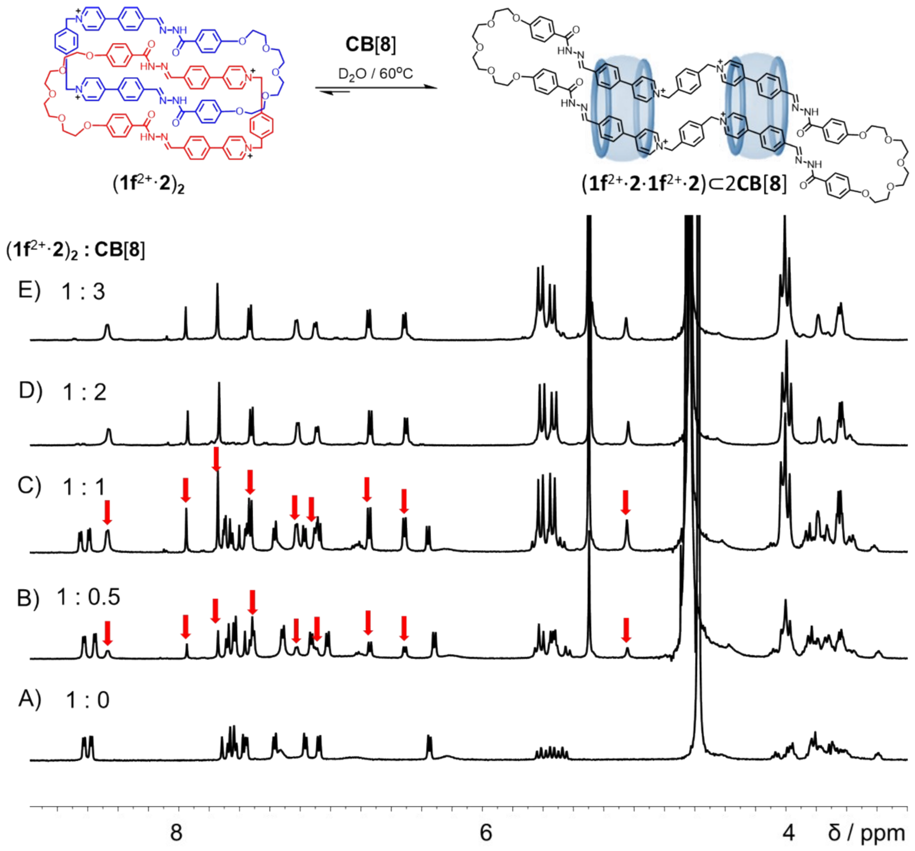


**Figure S57.** The $^{1}H$ NMR spectrum (500 MHz, $D_2O$, 298 K) of the [2]catenane (**1f**$^{2+}$·**2**)$_2$ after adding different amount of **CB**[8]. All spectra were recorded after the systems reached their equilibria. The ratio of (**1f**$^{2+}$·**2**)$_2$ and **CB**[8] is A) 1:0, B) 1:0.5, C) 1:1, D) 1:2 and E) 1:3. The resonances corresponding to (**1f**$^{2+}$·**2**·**1f**$^{2+}$·**2**)⊂2**CB**[8] are marked with red arrows.

We added different equivalents of **CB**[8] into [2]catenane (**1f**$^{2+}$·**2**)$_2$ at 60 °C for 24 h in $D_2O$. $^{1}H$ NMR spectra indicate that, when the **CB**[8]/catenane ratio is less than 2:1, both (**1f**$^{2+}$·**2**)$_2$ and (**1f**$^{2+}$·**2**·**1f**$^{2+}$·**2**)⊂2**CB**[8] were observed (Figure S57 B and C). No other products such as the [2]pseudorotaxane (**1f**$^{2+}$·**2**·**1f**$^{2+}$·**2**)⊂**CB**[8] was observed, indicating that the formation of the [3]pseudorotaxane (**1f**$^{2+}$·**2**·**1f**$^{2+}$·**2**)⊂2**CB**[8] is positively cooperative. After the **CB**[8]/catenane ratio reached 2:1, further addition of **CB**[8] did not lead to any changes in the $^{1}H$ NMR spectrum (Figure S57 E).

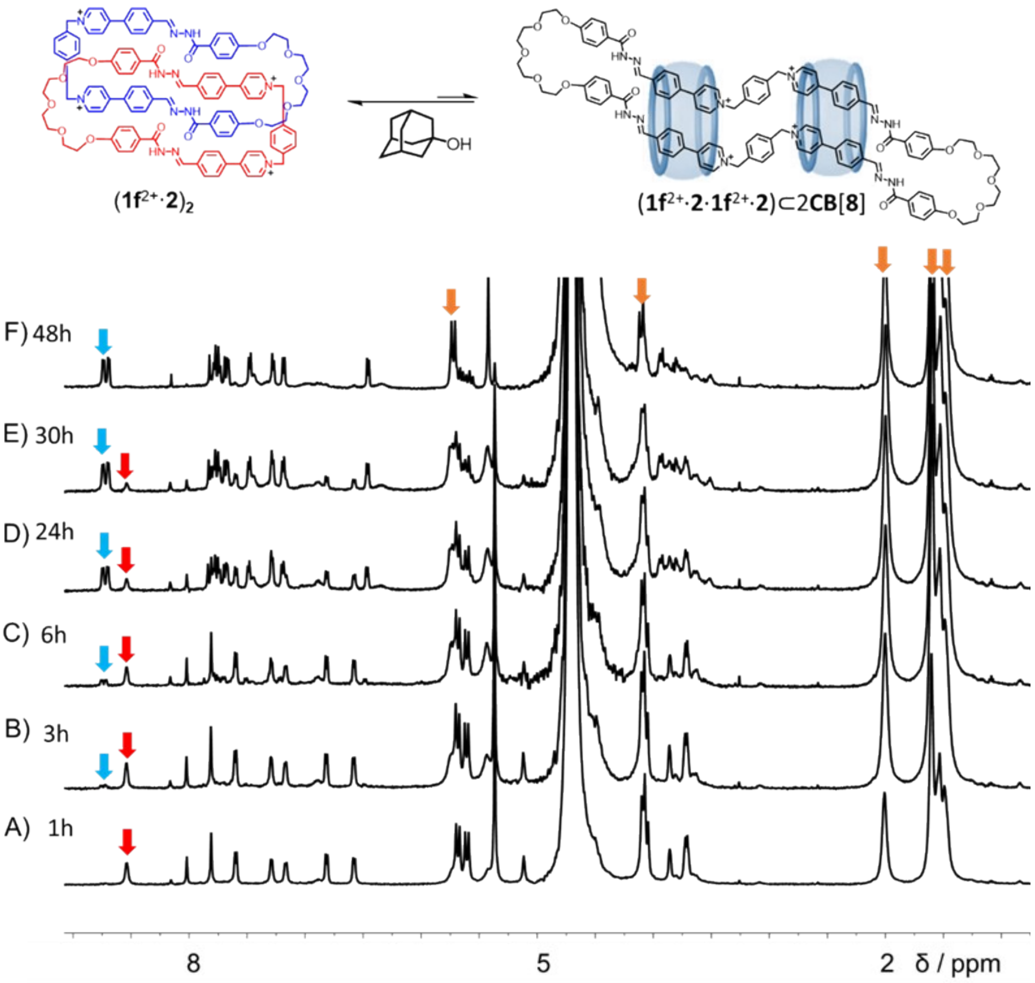


**Figure S58.** $^1$H NMR spectra (500 MHz, $D_2O$, 298 K) of (**1f**$^{2+}$·**2**·**1f**$^{2+}$·**2**)⊂2**CB**[**8**] after removing the **CB[8]** ring by adding excess amount of adamantan-1-ol for certain amount of time. The resonances of (**1f**$^{2+}$**·2**)$_2$, (**1f**$^{2+}$·**2**·**1f**$^{2+}$·**2**)⊂2**CB**[**8**] and adamantan-1-ol⊂**CB**[**8**] are marked with blue arrows, red arrows and orange arrows, respectively., indicating that after removing the **CB[8]** ring, the [2]catenane was gradually recovered within 48 h.

## 5. The process of generating (**1f**$^{2+}$·**2**·**1f**$^{2+}$·**2**)⊂2**CB[8]** by means of one-pot procedure

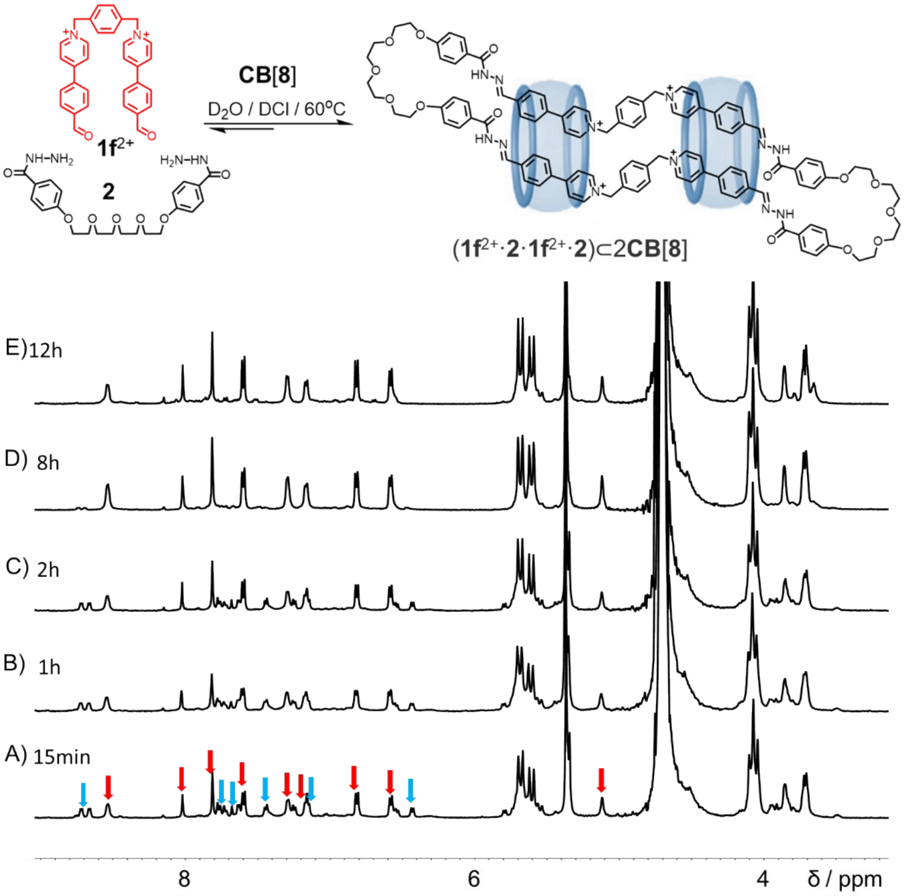


**Figure S59.** The $^1$H NMR spectra (500 MHz, $D_2O$, 298 K) of a 1:1:1 mixture of **1f**$^{2+}$·2Cl$^-$, **2** and **CB**[8], after mixing the reactants for certain amount of time. The resonances of (**1f**$^{2+}$·**2**)$_2$ and (**1f**$^{2+}$·**2**·**1f**$^{2+}$·**2**)⊂2**CB**[8] are marked with blue arrows and red arrows, respectively.

# 6. Yields of the condensation of **2** and a series of biscationic dialdehydes

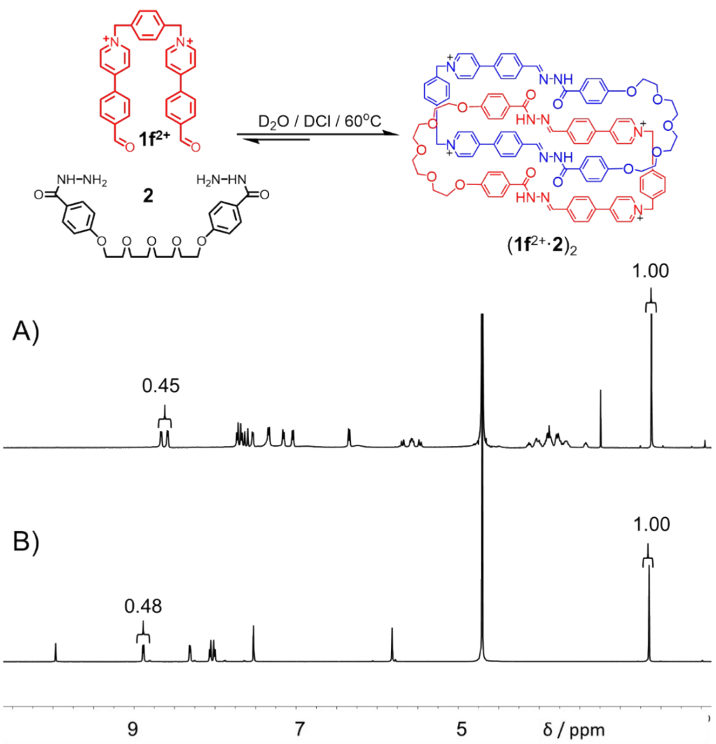

**Figure S60**. $^{1}H$ NMR spectra (500 MHz, $D_2O$, 298 K) of A) (**1f**$^{2+}$·**2**)$_2$ and B) **1f**$^{2+}$·2Cl$^{-}$.

We used $^{1}H$ NMR spectra to calculate the self-assembly yields by using DMSO with a fixed concentration as the internal standard. The integrated intensity of the resonance of internal standard was normalized to 1, allowing the concentration ratios of the self-assembled products relative to their corresponding aldehyde precursors to be calculated. For example, in the $^{1}H$ NMR spectra (Figure S60) of **1f**$^{2+}$·2Cl$^{-}$ and (**1f**$^{2+}$·**2**)$_2$·4Cl$^{-}$, whose spectra were recorded before and after adding **2**, the resonances of the hydrogens in pyridine *α* position were integrated as 0.48 and 0.45, respectively, relative to the standard; the yield of (**1f**$^{2+}$·**2**)$_2$·4Cl$^{-}$ was thus calculated to be 94% (0.45/0.48). The yields mentioned below were all calculated according to this method.

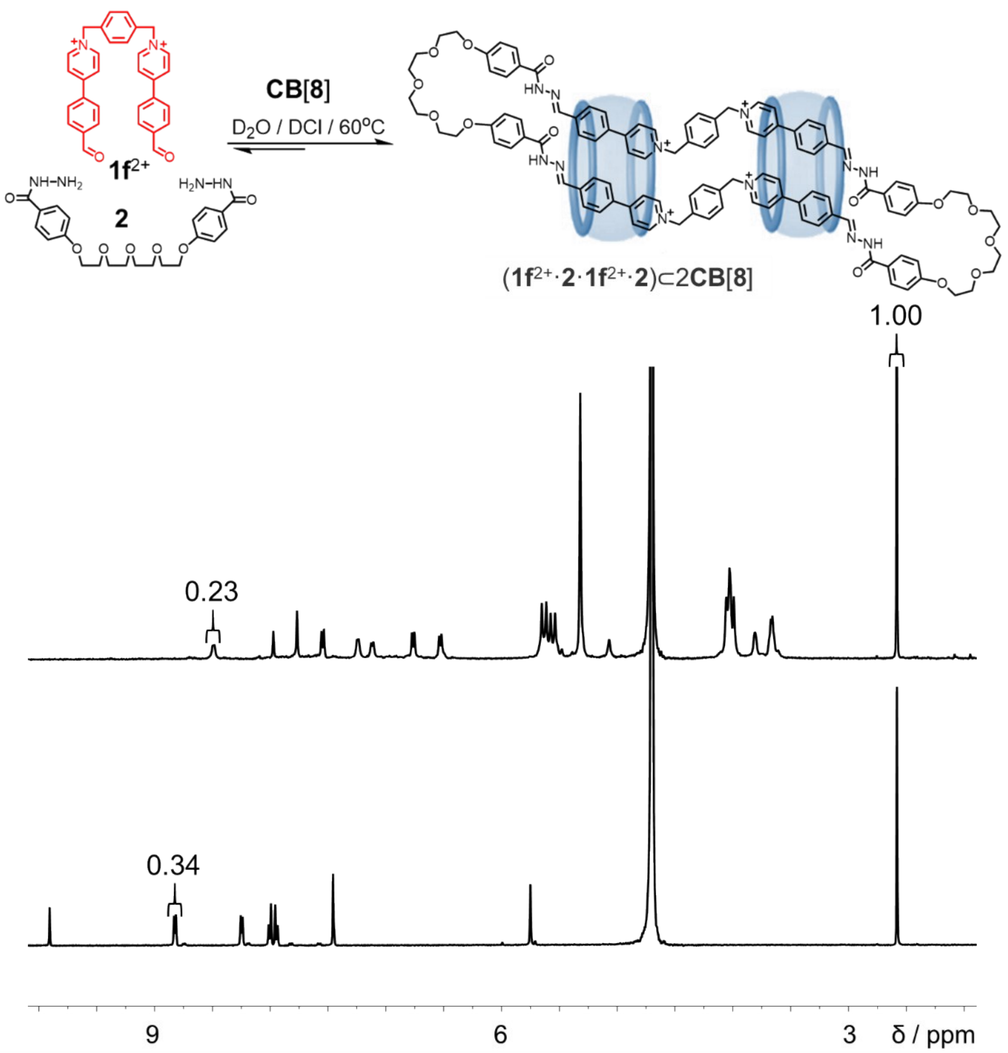


**Figure S61**. $^{1}$H NMR spectra (400 MHz, $D_2O$, 298 K) of A) (**1f**$^{2+}$·**2**·**1f**$^{2+}$·**2**)⊂2**CB**[**8**], B) **1f**$^{2+}$·2Cl$^-$. The NMR yield of (**1f**$^{2+}$·**2**·**1f**$^{2+}$·**2**)⊂2**CB**[**8**] is 68%.

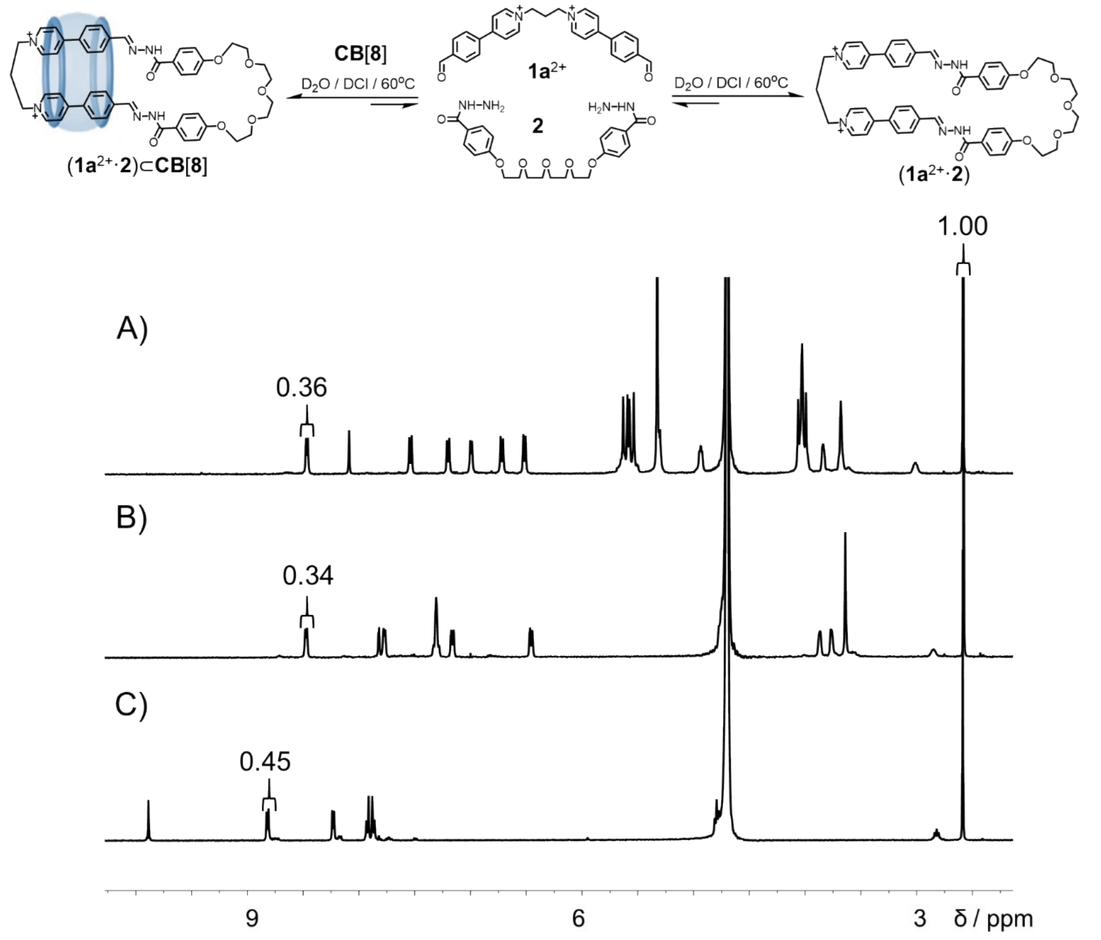


**Figure S62**. $^1$H NMR spectra (400 MHz, $D_2O$, 298 K) of A) (**1a**$^{2+}$·**2**)⊂**CB**[**8**], B) (**1a**$^{2+}$·**2**) and C) **1a**$^{2+}$·2Br$^-$. The NMR yields of (**1a**$^{2+}$·**2**)⊂**CB**[**8**] and (**1a**$^{2+}$·**2**) are 80% and 76%, respectively.

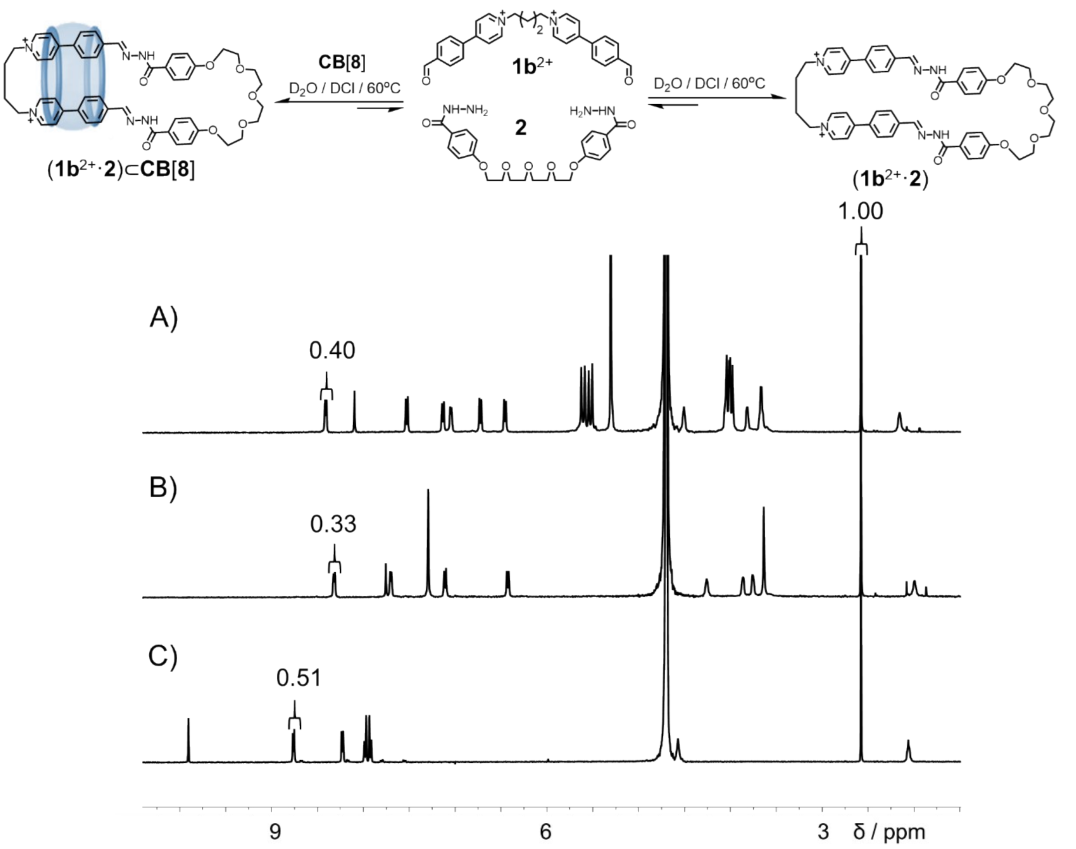


**Figure S63**. $^{1}$H NMR spectra (400 MHz, $D_2O$, 298 K) of A) (**1b**$^{2+}$·**2**)⊂**CB**[**8**], B) (**1b**$^{2+}$·**2**) and C) **1b**$^{2+}$·2Br$^{-}$. The NMR yields of (**1b**$^{2+}$·**2**)⊂**CB**[**8**] and (**1b**$^{2+}$·**2**) are 74% and 65%, respectively.

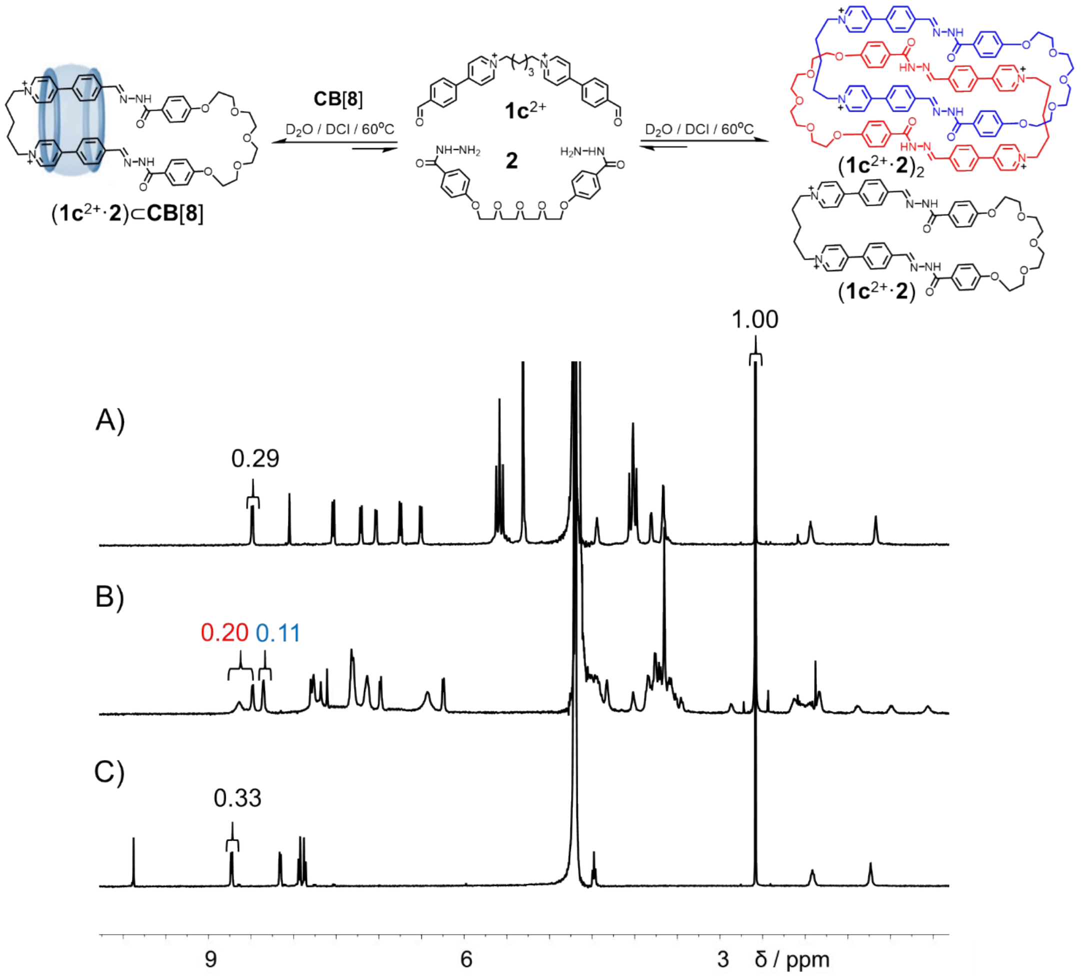


**Figure S64**. $^{1}$H NMR spectra (400 MHz, $D_2O$, 298 K) of A) (**1c**$^{2+}$·**2**)⊂**CB**[**8**], B) the reaction mixture by condensing a 1:1 mixture of **1c**$^{2+}$2Br⁻ (2.5 mM) and **2** (2.5 mM) and C) **1c**$^{2+}$·2Br⁻. The NMR yields of (**1c**$^{2+}$·**2**)⊂**CB**[**8**], (**1c**$^{2+}$·**2**)$_2$ (marked with red colors) and (**1c**$^{2+}$·**2**) (marked with blue colors) are 88%, 61% and 33%, respectively.

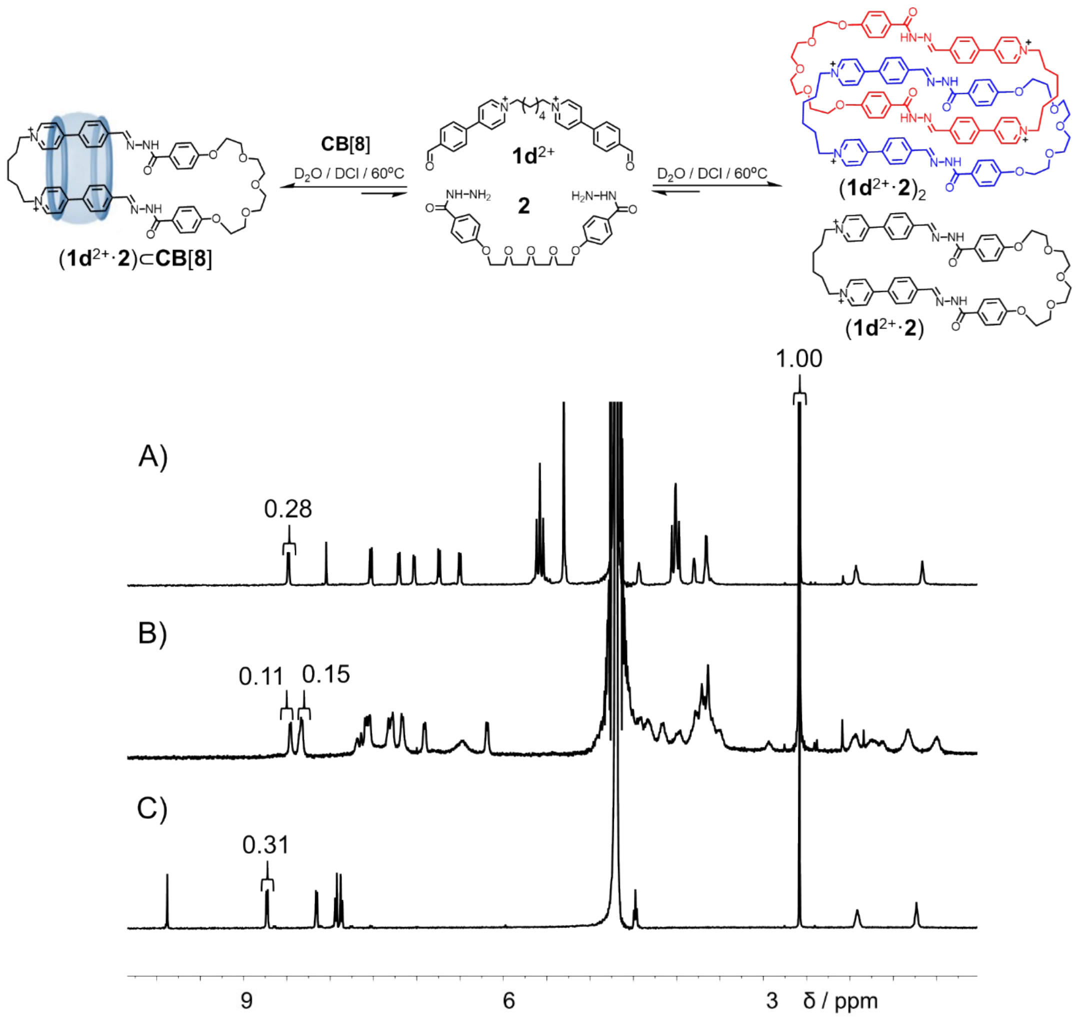


**Figure S65**. $^1$H NMR spectra (400 MHz, $D_2O$, 298 K) of A) (**1d**$^{2+}$·**2**)⊂**CB[8]**, B) the reaction mixture by condensing a 1:1 mixture of **1d**$^{2+}$2Br$^-$ (2.5 mM) and **2** (2.5 mM) and C) **1d**$^{2+}$·2Br$^-$. The NMR yield of (**1d**$^{2+}$·**2**)⊂**CB[8]** is 90%. However, the peaks of the [2]catenane (**1d**$^{2+}$·**2**)$_2$ and the macrocycle (**1d**$^{2+}$·**2**) are overlapped in the spectrum B), so that the NMR yields of them can only be roughly estimated: 71% for (**1d**$^{2+}$·**2**)$_2$ (0.11x2/0.31), 13% for (**1d**$^{2+}$·**2**) {(0.15 — 0.11)/0.31}.

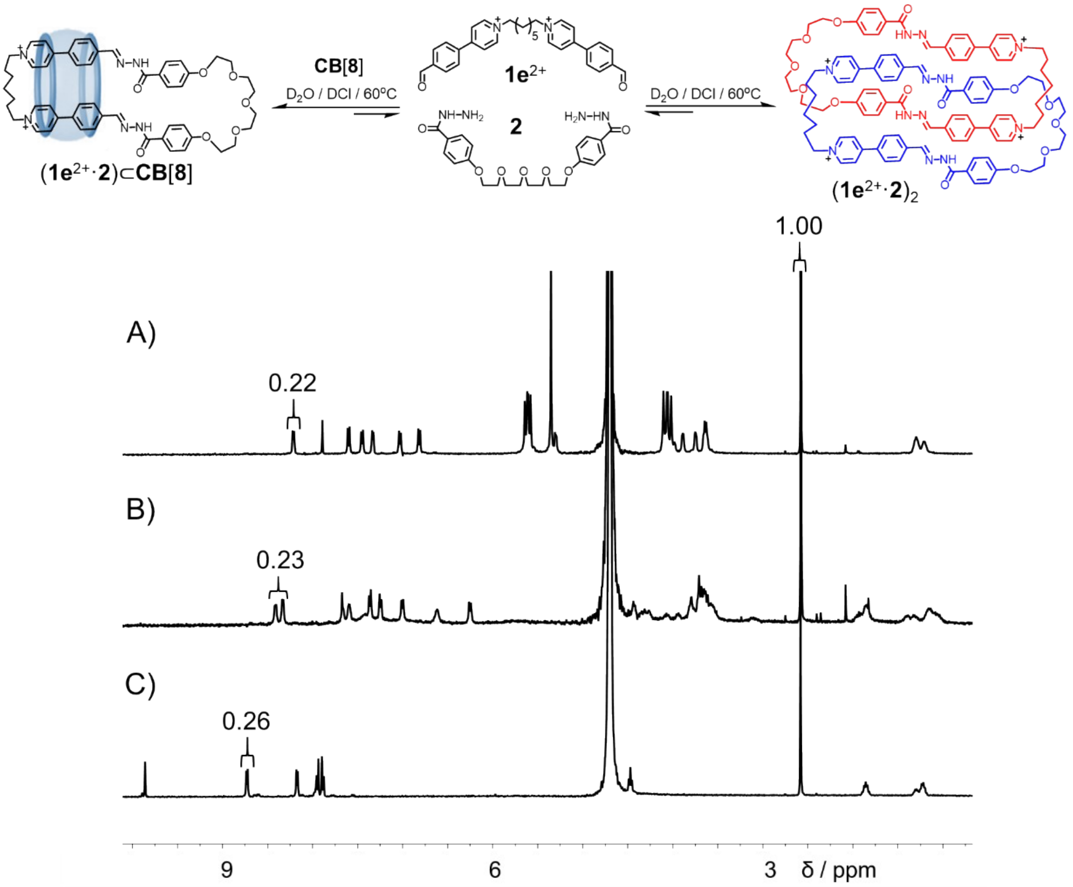


**Figure S66**. $^{1}$H NMR spectra (400 MHz, $D_2O$, 298 K) of A) (**1e**$^{2+}$·**2**)⊂**CB[8]**, B) (**1e**$^{2+}$·**2**)$_2$ and C) **1e**$^{2+}$·2Br$^-$. The NMR yields of (**1e**$^{2+}$·**2**)⊂**CB[8]** and (**1e**$^{2+}$·**2**)$_2$ are 85% and 88%, respectively.

# 7. Competitive experiments

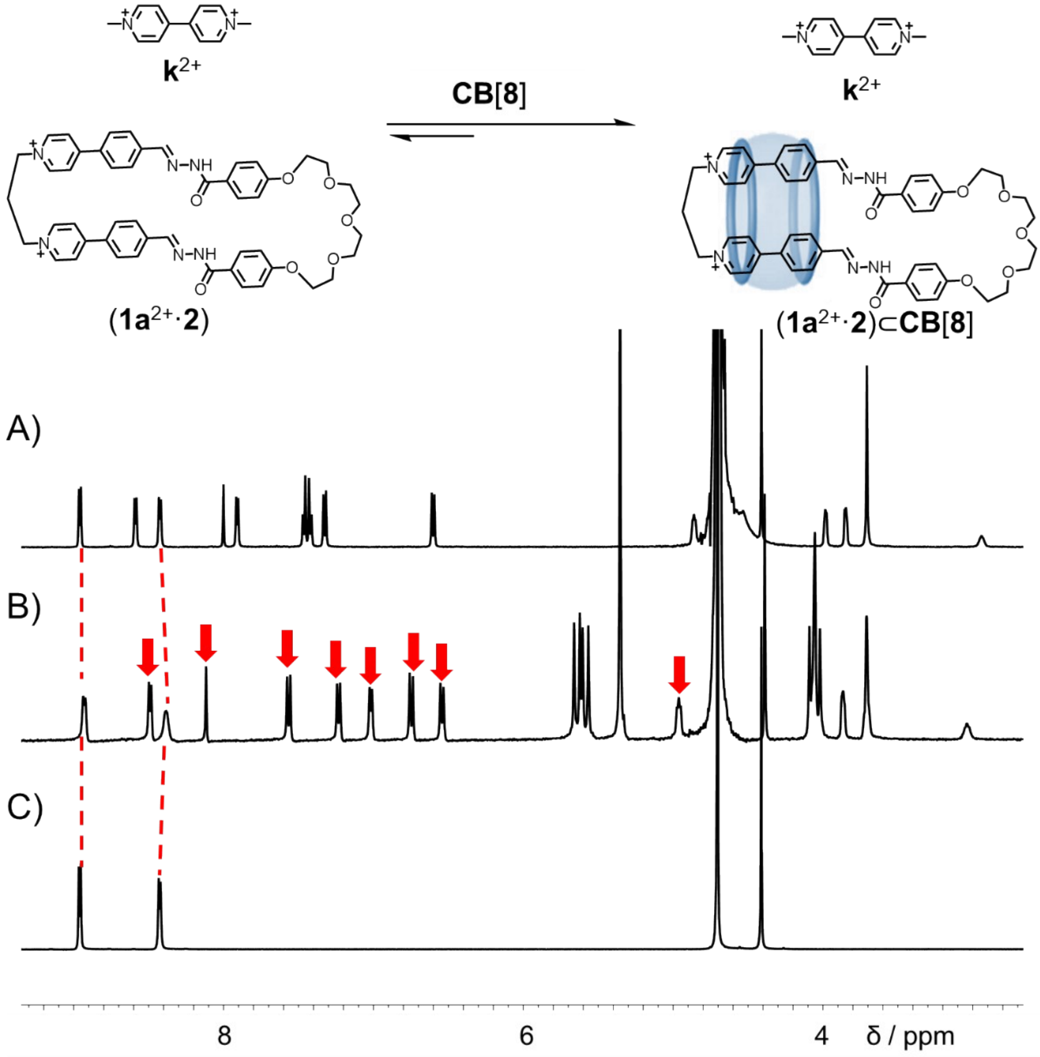


**Figure S67.** The $^1$H NMR spectra (500 MHz, $D_2O$, 298 K) of the 1:1 mixture of the macrocycle (**1a**$^{2+}$·**2**) and paraquat **k**$^{2+}$, A) before and B) after adding 1 equiv. of **CB**[**8**]. C) The $^1$H NMR spectrum (500 MHz, $D_2O$, 298 K) of paraquat **k**$^{2+}$. The resonances of (**1a**$^{2+}$·**2**)⊂**CB**[**8**] are marked with red arrows in B).

To a 1:1 mixture of the macrocycle (**1a**$^{2+}$·**2**) and paraquat **k**$^{2+}$, 1 equiv (relative to (**1a**$^{2+}$·**2**) ) of **CB**[**8**] was added. After the system reached the equilibrium, the macrocycle (**1a**$^{2+}$·**2**) was recognized within the cavity of **CB[8]**, forming a [2]pseudorotaxane (**1a**$^{2+}$·**2**)⊂**CB**[**8**]. In contrast, the resonances corresponding to paraquat **k**$^{2+}$ barely shifted (Figure S67 B). This experiment indicates that **CB**[**8**] has a stronger binding affinity to recognize (**1a**$^{2+}$·**2**) than **k**$^{2+}$.

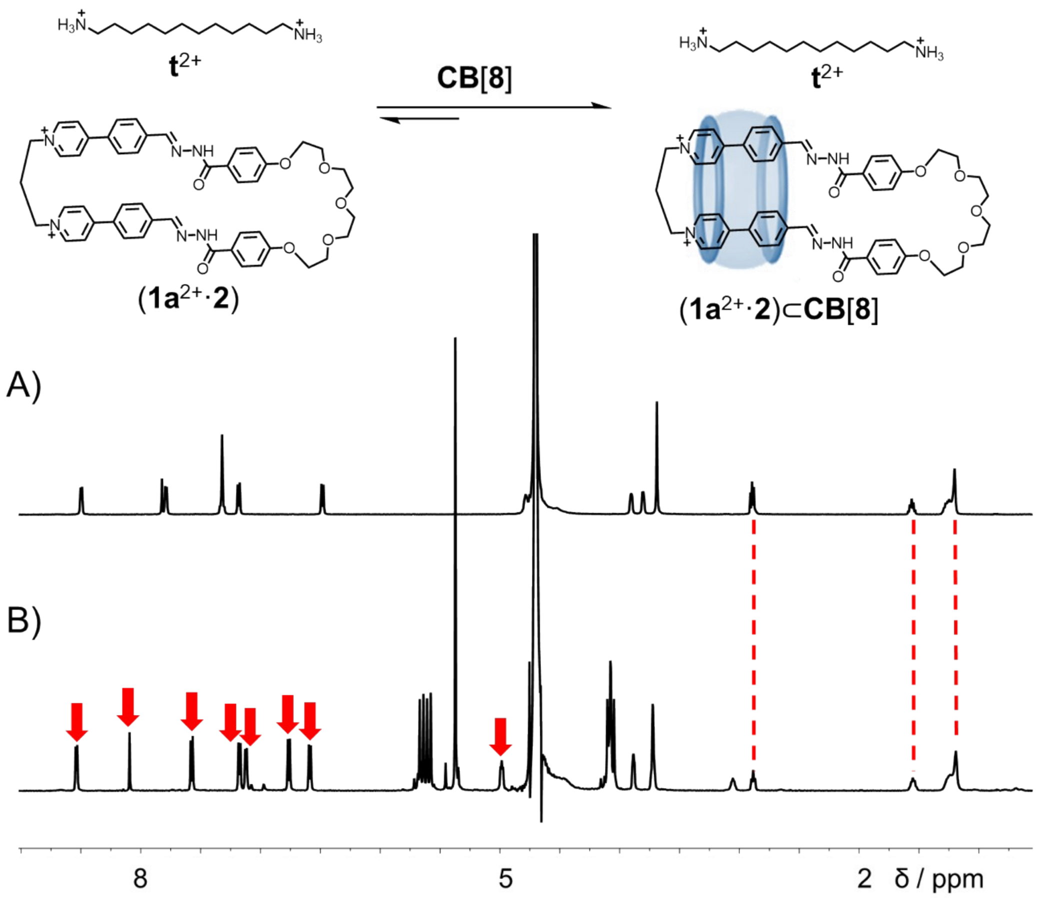


**Figure S68.** The $^{1}$H NMR spectra (500 MHz, $D_2O$, 298 K) of the 1:1 mixture of the macrocycle (**1a**$^{2+}$·**2**) and **t**$^{2+}$, A) before and B) after adding 1 equiv. of **CB**[**8**]. The resonances of (**1a**$^{2+}$·**2**)⊂**CB**[**8**] are marked with red arrows in B).

To a 1:1 mixture of the macrocycle (**1a**$^{2+}$**·2**) and **t**$^{2+}$, 1 equiv (relative to (**1a**$^{2+}$·**2**)) of **CB[8]** was added. After the system reached the equilibrium, the macrocycle (**1a**$^{2+}$·**2**) was recognized within the cavity of **CB[8]**, forming a [2]pseudorotaxane (**1a**$^{2+}$·**2**)⊂**CB**[**8**]. In contrast, the resonances corresponding to **t**$^{2+}$·2Cl$^{-}$ barely shifted (Figure S68 B). This experiment indicates that **CB**[**8**] has a stronger binding affinity to recognize (**1a**$^{2+}$·**2**) than **t**$^{2+}$.

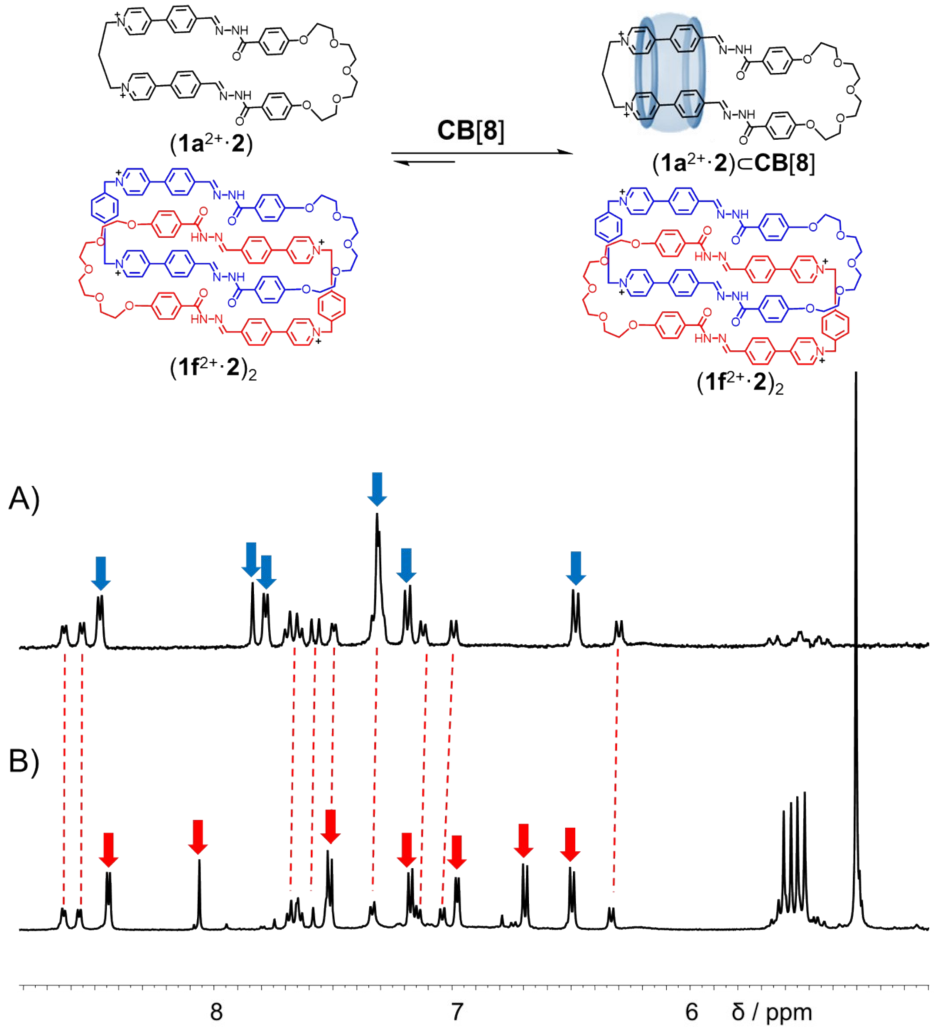


**Figure S69.** The $^{1}H$ NMR spectra (500 MHz, $D_2O$, 298 K) of the 1:0.5 mixture of the macrocycle (**1a**$^{2+}$·**2**) and (**1f**$^{2+}$·**2**)$_2$, A) before and B) after adding 1 equiv. of **CB**[**8**]. The spectrum in B) were recorded after the system reached its equilibrium. The resonances of (**1a**$^{2+}$·**2**) are marked with blue arrows in A) and the resonances of (**1a**$^{2+}$·**2**)⊂**CB**[**8**] are marked with red arrows in B).

To a 1:0.5 mixture of the macrocycle (**1a**$^{2+}$**·2**) and (**1f**$^{2+}$·**2**)$_2$, 1 equiv (relative to (**1a**$^{2+}$·**2**) ) of **CB**[**8**] was added. After the system reached the equilibrium, the macrocycle (**1a**$^{2+}$·**2**) was recognized within the cavity of **CB[8]**, forming a [2]pseudorotaxane (**1a**$^{2+}$·**2**)⊂**CB[8]**. In contrast, the resonances corresponding to (**1f**$^{2+}$·**2**)$_2$ barely shifted (Figure S69 B). This experiment indicates that **CB**[**8**] has a stronger binding affinity to recognize (**1a**$^{2+}$·**2**) than (**1f**$^{2+}$·**2**)$_2$.

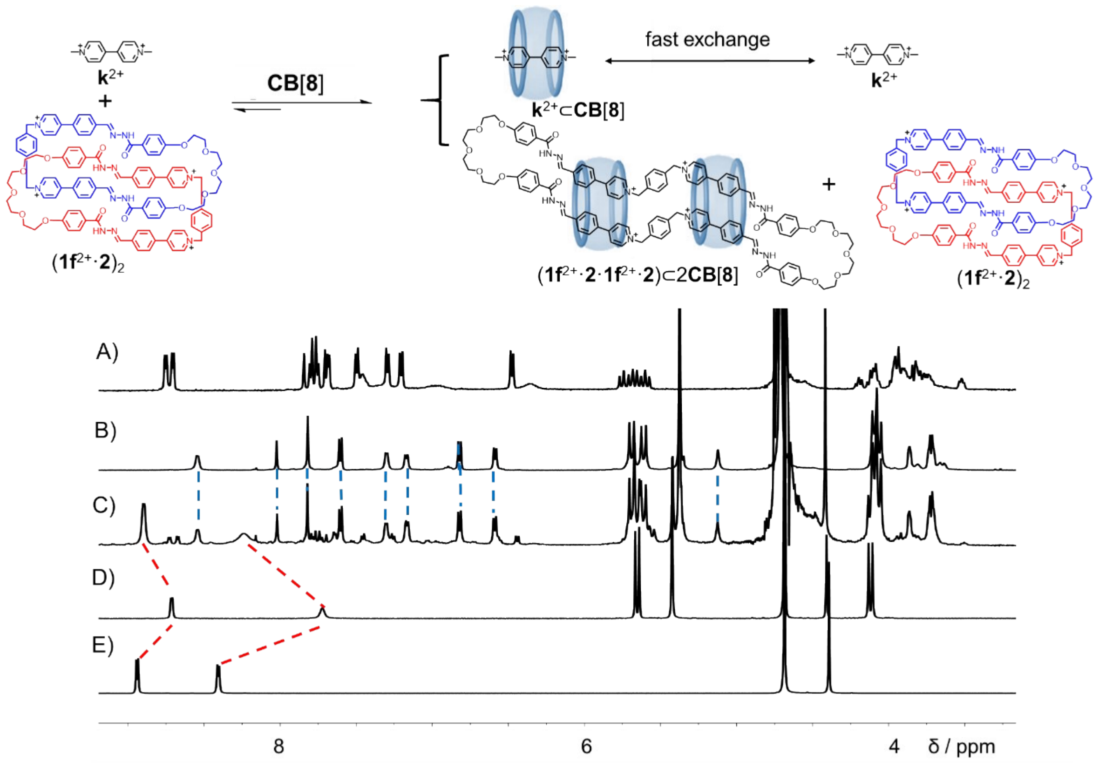


**Figure S70.** The $^{1}$H NMR spectra (500 MHz, $D_2O$, 298 K) of A) (**1f**$^{2+}$·**2**)$_2$, B) (**1f**$^{2+}$·**2**·**1f**$^{2+}$·**2**)⊂2**CB**[**8**], C) the reaction mixture of (**1f**$^{2+}$·**2**)$_2$, paraquat **k**$^{2+}$ and **CB**[**8**] at the ratio of 0.5:1:1 , D) the reaction mixture of paraquat **k**$^{2+}$ and **CB**[**8**] at the ratio of 1:1 and E) paraquat **k**$^{2+}$. All the spectra were recorded after the equilibria were established.

A 0.5:1:1 mixture of the [2]catenane (**1f**$^{2+}$·**2**)$_2$, paraquat **k**$^{2+}$, and **CB**[**8**] were combined in $D_2O$. After the system reached the equilibrium, we observed that only small amount of **CB**[**8**] recognized **k**$^{2+}$ to form **k**$^{2+}$⊂**CB**[**8**] (Figure S70 C), as inferred from the observation that the resonances corresponding to **k**$^{2+}$ underwent minor upfield shifts, compared to those of the spectrum D). (**1f**$^{2+}$·**2**·**1f**$^{2+}$·**2**)⊂2**CB**[**8**] (Figure S70 C) were self-assembled as one of the major product, accompanied with the unreacted [2]catenane. This experiment indicates that the binding constants of (**1f**$^{2+}$·**2**·**1f**$^{2+}$·**2**)⊂2**CB**[**8**] and **k**$^{2+}$⊂**CB**[**8**] might be comparable, the former of which might be slightly larger in a qualitative analysis.

# 8. UV-Vis Spectroscopic Analysis

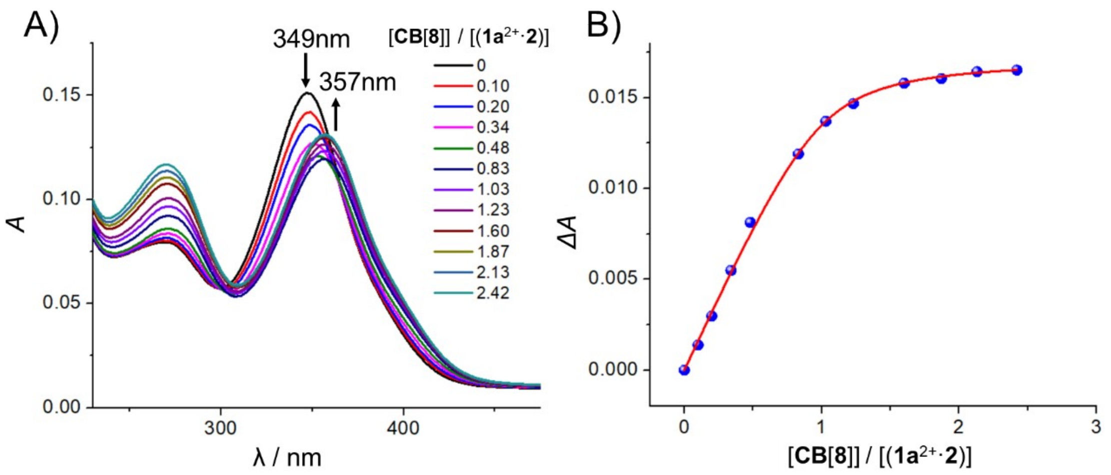


**Figure S71**. A) UV/Vis absorption spectra of (**1a**$^{2+}$·**2**)·2Cl$^{-}$ after adding different amount of **CB**[8] in $H_2O$ at 298 K. B) Plot of the absorbance intensity at λ=400 nm verus [**CB**[**8**]] / [(**1a**$^{2+}$·**2**)]. The concentration of the guest was kept constant for all spectra, namely [(**1a**$^{2+}$·**2**)] = 3.0×10$^{-6}$ M.

UV-Vis spectroscopic studies were performed to investigate the binding affinity of the complex (**1a**$^{2+}$·**2**)·2Cl$^{-}$⊂**CB**[8]. The association constant of complex was determined by probing the charge-transfer band of the complex (Figure S71A) and fitting the change of absorbance with a 1:1 binding curve by using the Benesie-Hildebrand method. The binding constant $K_a$ of (**1a**$^{2+}$·**2**)·2Cl$^{-}$ and **CB**[8] was calculated to be around 5.6(±1.1)×10$^{6}$ M$^{-1}$ in $D_2O$ (Figure S71B).

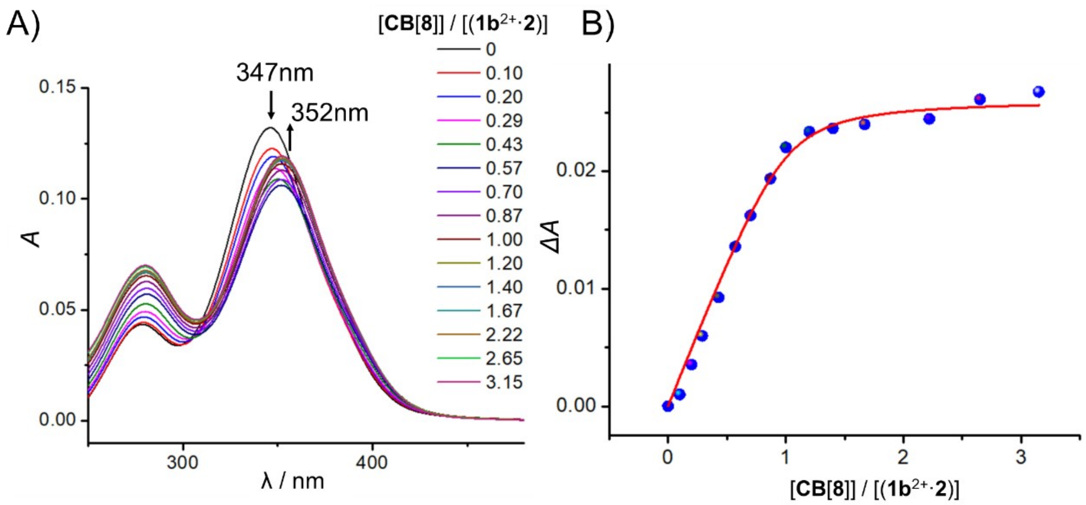


**Figure S72**. A) UV/Vis absorption spectra of (**1b**$^{2+}$·**2**)·2Cl$^{-}$ after adding different amount of **CB**[8] in $H_2O$ at 298 K. B) Plot of the absorbance intensity at λ=400 nm verus [**CB**[8]] / [(**1b**$^{2+}$·**2**)]. The concentration of the guest was kept constant for all spectra, namely [(**1b**$^{2+}$·**2**)] = 3.0×10$^{-6}$ M.

UV-Vis spectroscopic studies were performed to investigate the binding affinity of the complex (**1b**$^{2+}$·**2**)·2Cl$^{-}$⊂**CB**[8]. The association constant of complex was determined by probing the charge-transfer band of the complex (Figure S72A) and fitting the change of absorbance with a 1:1 binding curve by using the Benesie-Hildebrand method. The binding constant $K_a$ of (**1b**$^{2+}$·**2**)·2Cl$^{-}$ and **CB**[8] was calculated to be around 7.3(±2.9)×10$^{6}$ M$^{-1}$ in $D_2O$ (Figure S72B).

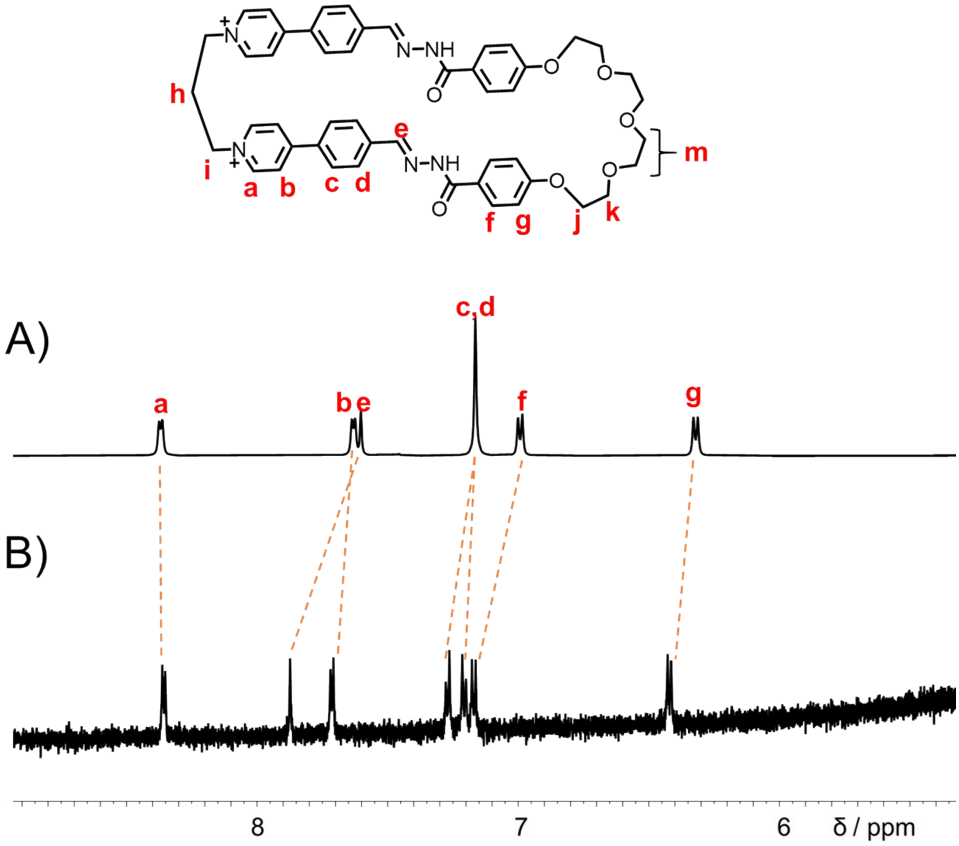


**Figure S73.** Partial $^{1}H$ NMR spectrum of ($\mathbf{1a}^{2+}\cdot\mathbf{2}$)·2Cl⁻ at different concentrations (600 MHz, $D_2O$, 298 K), including A) 2.5 mM and B) $3\times10^{-3}$ mM.

# 9. X-ray Crystallography

1) ($\mathbf{1f}^{2+}\cdot\mathbf{2}$)$_2\cdot$4Cl$^-$

1.1) Methods

Single crystals of the [2]catenane ($\mathbf{1f}^{2+}\cdot\mathbf{2}$)$_2\cdot$4Cl$^-$ was obtained by slow vapor diffusion of THF into the water solution under room temperature. A suitable crystal was selected on a Bruker D8 Venture diffractometer. The crystal was kept at 170.0 K during data collection. Using Olex2 [1], the structure was solved with the ShelXT [2] structure solution program using Intrinsic Phasing and refined with the ShelXL [3] refinement package using Least Squares minimisation.

1.2) Crystal data

[$C_{108}H_{128}Cl_4N_{12}O_{26}$] (*M* =2152.02 g/mol): monoclinic, space group $P2_{1/c}$ (no. 14), *a* = 13.6939(9) Å, *b* = 19.0018(12) Å, *c* = 50.945(3) Å, $\alpha$ = 90°, $\beta$ = 90.412(4)°, $\gamma$ = 90°, *V* = 13256.0(15) Å$^{3,}$ *Z* = 4, *T* = 170.0 K, *μ*(GaKα) = 0.876 mm$^{-1}$, *Dcalc* = 1.078 g/cm$^3$, 122205 reflections measured (5.614° ≤ 2Θ ≤ 96.354°), 18873 unique ($R_{int}$ = 0.1553, $R_{sigma}$ = 0.1168) which were used in all calculations. The final $R_1$ was 0.1224 (I > 2σ(I)) and $wR_2$ was 0.3812 (all data). CCDC number: 2014868.

**Table S1 Crystal data and structure refinement for** ($\mathbf{1f}^{2+}\cdot\mathbf{2}$)$_2\cdot$4Cl$^-$

| | |
|---|---|
| Empirical formula | $C_{108}H_{128}Cl_4N_{12}O_{26}$ |
| Formula weight | 2152.02 |
| Temperature/K | 170.0 |
| Crystal system | monoclinic |
| Space group | $P2_1/c$ |
| a/Å | 13.6939(9) |
| b/Å | 19.0018(12) |
| c/Å | 50.945(3) |
| α/° | 90 |
| β/° | 90.412(4) |

| | |
|---|---|
| γ/° | 90 |
| Volume/Å$^3$ | 13256.0(15) |
| Z | 4 |
| $\rho_{calc}$g/cm$^3$ | 1.078 |
| μ/mm$^{-1}$ | 0.876 |
| F(000) | 4544.0 |
| Crystal size/mm$^3$ | 0.05 × 0.02 × 0.015 |
| Radiation | GaKα (λ = 1.34139) |
| 2Θ range for data collection/° | 5.614 to 96.354 |
| Index ranges | -15 ≤ h ≤ 15, -21 ≤ k ≤ 21, -56 ≤ l ≤ 49 |
| Reflections collected | 122205 |
| Independent reflections | 18873 [$R_{int}$ = 0.1553, $R_{sigma}$ = 0.1168] |
| Data/restraints/parameters | 18873/96/1414 |
| Goodness-of-fit on F$^2$ | 1.086 |
| Final R indexes [I>=2σ (I)] | $R_1$ = 0.1224, w$R_2$ = 0.3055 |
| Final R indexes [all data] | $R_1$ = 0.2392, w$R_2$ = 0.3812 |
| Largest diff. peak/hole / e Å$^{-3}$ | 1.47/-0.61 |

1.3) Solid-state structure

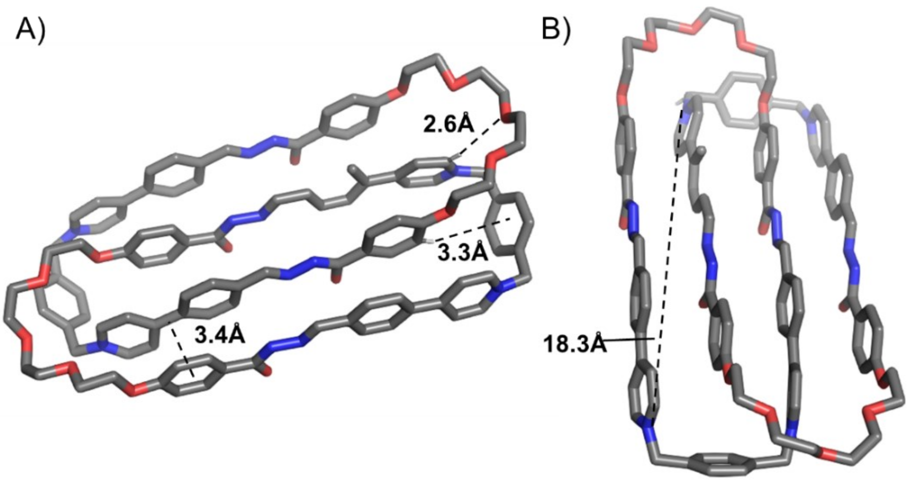


**Figure S74**. Different views of the solid-state structure of (**1f**$^{2+}$·**2**)$_2$·4Cl$^-$. Counterions and solvent molecules are omitted for the sake of clarity.

2) (**1f**$^{2+}$·**2**·**1f**$^{2+}$·**2**)⊂2**CB**[**8**]

2.1) Methods

Single crystals of the ring in ring complex (**1f**$^{2+}$·**2**·**1f**$^{2+}$·**2**)⊂2**CB**[**8**] was obtained by slow evaporation of the solution of (**1f**$^{2+}$·**2**·**1f**$^{2+}$·**2**)⊂2**CB**[**8**] in water under room temperature. Charges are balanced by chloride counteranions. A suitable crystal was selected on a Bruker D8 Venture diffractometer. The crystal was kept at 170.0 K during data collection. Using Olex2 [1], the structure was solved with the ShelXT [2] structure solution program using Intrinsic Phasing and refined with the ShelXL [3] refinement package using Least Squares minimisation.

2.2) Crystal data

[$C_{102}H_{102}Cl_2N_{38}O_{25}$] (*M* =2331.11 g/mol): Triclinic, space group P-1, *a* = 14.5032(6) Å, *b* = 23.2785(10) Å, *c* = 23.8851(11) Å, $\alpha$ = 107.441(3)°, $\beta$ = 93.266(3)°, $\gamma$ = 104.627(3)°, *V* = 7366.6(6) Å$^3$, *Z* = 2, *T* = 170.02 K, $\mu$(GaKα) = 0.629 mm$^{-1}$, *Dcalc* =1.051 Mg/m$^3$, Full-matrix least-squares on $F^2$ reflections measured (2.845° ≤ 2Θ ≤ 55.148°), 27956 unique ($R_{int}$ = 0.0745) which were used in all calculations. The final $R_1$ was 0.1686 (I > 2σ(I)) and $wR_2$ was 0.3908 (all data). CCDC number: 2095808.

**Table S2 Crystal data and structure refinement for** (**1f**$^{2+}$·**2**·**1f**$^{2+}$·**2**)⊂2**CB**[**8**]

| | | |
|---|---|---|
| Empirical formula | $C_{102} H_{102} Cl_2 N_{38} O_{25}$ | |
| Formula weight | 2331.11 | |
| Temperature | 173.02 K | |
| Wavelength | 1.34139 Å | |
| Crystal system | Triclinic | |
| Space group | P-1 | |
| Unit cell dimensions | a = 14.5032(6) Å | a= 107.441(3)°. |
| | b = 23.2785(10) Å | b= 93.266(3)°. |
| | c = 23.8851(11) Å | g = 104.627(3)°. |
| Volume | 7366.6(6) Å$^3$ | |
| Z | 2 | |
| Density (calculated) | 1.051 Mg/m$^3$ | |
| Absorption coefficient | 0.629 mm$^{-1}$ | |

| | |
|---|---|
| F(000) | 2428 |
| Crystal size | 0.06 x 0.06 x 0.05 $mm^3$ |
| Theta range for data collection | 2.845 to 55.148°. |
| Index ranges | -17<=h<=15, -28<=k<=28, -29<=l<=29 |
| Reflections collected | 93616 |
| Independent reflections | 27956 [R(int) = 0.0745] |
| Completeness to theta = 53.594° | 99.6 % |
| Absorption correction | Semi-empirical from equivalents |
| Max. and min. transmission | 0.7508 and 0.5498 |
| Refinement method | Full-matrix least-squares on $F^2$ |
| Data / restraints / parameters | 27956 / 76 / 1498 |
| Goodness-of-fit on $F^2$ | 1.159 |
| Final R indices [I>2sigma(I)] | R1 = 0.1686, wR2 = 0.3688 |
| R indices (all data) | R1 = 0.2159, wR2 = 0.3908 |
| Extinction coefficient | n/a |
| Largest diff. peak and hole | 1.864 and -0.930 e.$Å^{-3}$ |

2.3) Solid-state structure

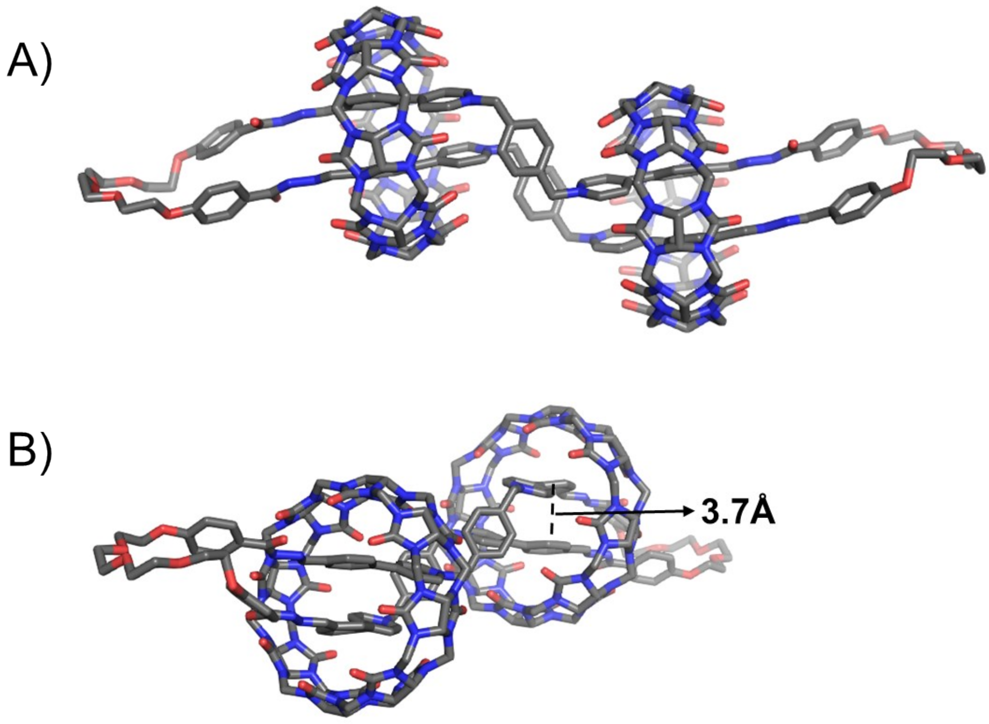

**Figure S75**. Different views of the solid-state structure of (**1f**$^{2+}$·**2**·**1f**$^{2+}$·**2**)⊂2**CB**[**8**]. Counterions and solvent molecules are omitted for the sake of clarity.

3) (**1a**$^{2+}$·**2**)·2Cl$^{-}$

3.1) Methods

Single crystals of the macrocycle (**1a**$^{2+}$·**2**)·2Cl$^{-}$ was obtained by slow vapor diffusion of acetone into the water solution under room temperature. A suitable crystal was selected on a Bruker D8 Venture diffractometer. The crystal was kept at 170.0 K during data collection. Using Olex2 [1], the structure was solved with the ShelXT [2] structure solution program using Intrinsic Phasing and refined with the ShelXL [3] refinement package using Least Squares minimisation.

3.2) Crystal data

[$C_{49}H_{51}Cl_2N_6O_{11.5}$] ($M$ =984.9 g/mol): Triclinic, space group P-1, $a$ = 10.3120(12) Å, $b$ = 20.687(2) Å, $c$ = 26.395(3) Å, $\alpha$ = 74.272(7)°, $\beta$ = 81.268(8)°, $\gamma$ = 78.693(8)°, $V$ = 5285.7(10) Å$^3$, $Z$ = 4, $T$ = 183.95 K, $\mu$(GaKα) = 1.054 mm$^{-1}$, $Dcalc$ =1.238 Mg/m$^3$, Full-matrix least-squares on $F^2$ reflections measured (3.182° ≤ 2Θ ≤ 55.148°), 19978 unique ($R_{int}$ = 0.1519) which were used in all calculations. The final $R_1$ was 0.1263 (I > 2σ(I)) and $wR_2$ was 0.4007 (all data). CCDC number: 2096625

**Table S3 Crystal data and structure refinement for** (**1a**$^{2+}$·**2**)·2Cl$^{-}$

| | | |
|---|---|---|
| Empirical formula | $C_{49}$ $H_{57}$ $Cl_2$ $N_6$ $O_{11.50}$ | |
| Formula weight | 984.90 | |
| Temperature | 183.95 K | |
| Wavelength | 1.34139 Å | |
| Crystal system | Triclinic | |
| Space group | P-1 | |
| Unit cell dimensions | a = 10.3120(12) Å | a= 74.272(7)°. |
| | b = 20.687(2) Å | b= 81.268(8)°. |
| | c = 26.395(3) Å | g = 78.693(8)°. |
| Volume | 5285.7(10) Å$^3$ | |
| Z | 4 | |
| Density (calculated) | 1.238 Mg/m$^3$ | |
| Absorption coefficient | 1.054 mm$^{-1}$ | |
| F(000) | 2076 | |
| Crystal size | 0.05 x 0.03 x 0.02 mm$^3$ | |
| Theta range for data collection | 3.182 to 55.148°. | |

| | |
|---|---|
| Index ranges | -12<=h<=11, -25<=k<=25, -32<=l<=32 |
| Reflections collected | 66904 |
| Independent reflections | 19978 [R(int) = 0.1519] |
| Completeness to theta = 53.594° | 99.7 % |
| Absorption correction | Semi-empirical from equivalents |
| Max. and min. transmission | 0.7508 and 0.3544 |
| Refinement method | Full-matrix-block least-squares on $F^2$ |
| Data / restraints / parameters | 19978 / 0 / 1267 |
| Goodness-of-fit on $F^2$ | 1.036 |
| Final R indices [I>2sigma(I)] | R1 = 0.1263, wR2 = 0.3271 |
| R indices (all data) | R1 = 0.2236, wR2 = 0.4007 |
| Extinction coefficient | n/a |
| Largest diff. peak and hole | 1.685 and -0.474 e.Å$^{-3}$ |

3.3) Solid-state structure

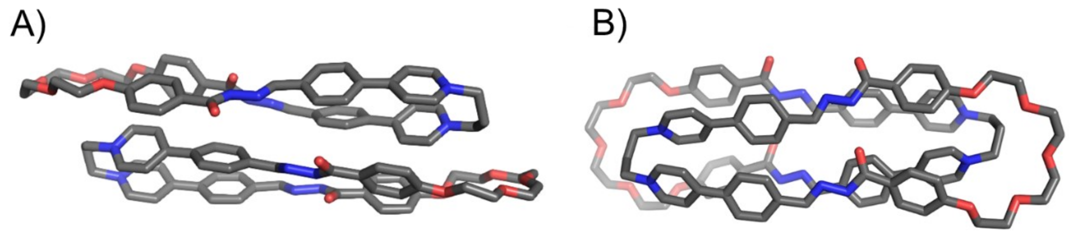


**Figure S76**. Different views of the solid-state structure of (**1a**$^{2+}$·**2**)·2Cl$^{-}$. Counterions and solvent molecules are omitted for the sake of clarity.

4) (**1b**$^{2+}$·**2**)·2PF$_6^{-}$

4.1) Methods

Single crystals of the [2]catenane (**1b**$^{2+}$·**2**)·2PF$_6^{-}$ was obtained by slow vapor diffusion of isopropyl ether into the acetonitrile solution under room temperature. A suitable crystal was selected on a Bruker D8 Venture diffractometer. The crystal was kept at 170.0 K during data collection. Using Olex2 [1], the structure was solved with the ShelXT [2] structure solution program using Intrinsic Phasing and refined with the ShelXL [3] refinement package using Least Squares minimisation.

4.2) Crystal data

[$C_{50}H_{52}F_{12}N_6O_7P_2$] ($M$ =1138.91 g/mol): Triclinic, space group P-1, $a$ = 9.22(2) Å, $b$ = 19.51(5) Å, $c$ = 20.82(5) Å, $\alpha$ = 62.99(5)°, $\beta$ = 86.96(6)°, $\gamma$ = 78.01(5)°, $V$ = 3260(14) Å$^3$, $Z$ = 2, $T$ = 174.82 K, $\mu$(GaKα) = 0.847 mm$^{-1}$, $Dcalc$ =1.160 Mg/m$^3$, Goodness-of-fit on $F^2$

reflections measured (3.698° ≤ 2Θ ≤ 49.650°), 10023 unique ($R_{int}$ = 0.2692) which were used in all calculations. The final $R_1$ was 0.1221 (I > 2σ(I)) and $wR_2$ was 0.4298 (all data).

CCDC number: 2096626

**Table S4 Crystal data and structure refinement for** (**1b**$^{2+}$·**2**)·2$PF_6^-$

| | | |
|---|---|---|
| Empirical formula | $C_{50} H_{52} F_{12} N_6 O_7 P_2$ | |
| Formula weight | 1138.91 | |
| Temperature | 174.82 K | |
| Wavelength | 1.34139 Å | |
| Crystal system | Triclinic | |
| Space group | P-1 | |
| Unit cell dimensions | a = 9.22(2) Å | a= 62.99(5)°. |
| | b = 19.51(5) Å | b= 86.96(6)°. |
| | c = 20.82(5) Å | g = 78.01(5)°. |
| Volume | 3260(14) Å$^3$ | |
| Z | 2 | |
| Density (calculated) | 1.160 Mg/m$^3$ | |
| Absorption coefficient | 0.847 mm$^{-1}$ | |
| F(000) | 1176 | |
| Crystal size | 0.08 x 0.02 x 0.01 mm$^3$ | |
| Theta range for data collection | 3.698 to 49.650°. | |
| Index ranges | -10<=h<=10, -22<=k<=22, -23<=l<=23 | |
| Reflections collected | 63457 | |
| Independent reflections | 10023 [R(int) = 0.2692] | |
| Completeness to theta = 49.650° | 100.0 % | |
| Absorption correction | Semi-empirical from equivalents | |
| Max. and min. transmission | 0.7508 and 0.5004 | |
| Refinement method | Full-matrix least-squares on F$^2$ | |
| Data / restraints / parameters | 10023 / 22 / 694 | |
| Goodness-of-fit on F$^2$ | 0.935 | |
| Final R indices [I>2sigma(I)] | R1 = 0.1221, wR2 = 0.3225 | |
| R indices (all data) | R1 = 0.2840, wR2 = 0.4298 | |
| Extinction coefficient | n/a | |
| Largest diff. peak and hole | 0.457 and -0.345 e.Å$^{-3}$ | |

4.3) Solid-state structure

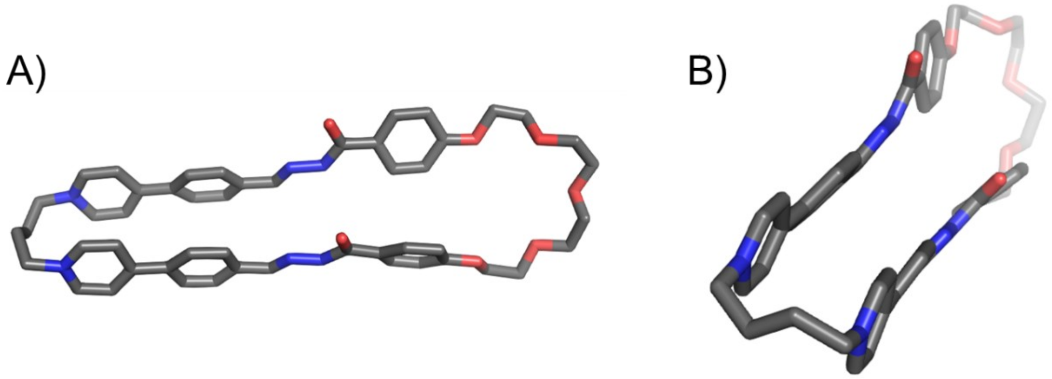


**Figure S77**. Different views of the solid-state structure of (**1b**$^{2+}$·**2**)·2$PF_6^-$. Counterions and solvent molecules are omitted for the sake of clarity.

## 10. References

1. D. Benito-Alifonso, B. Richichi, V. Baldoneschi, M. Berry, M. Fragai, G. Salerno, M. C. Galan, C. Nativi. *ACS Omega* 2018, **3**, 9822−9826.
